# Offshore solar-driven methane valorization to chloromethane with seawater

**DOI:** 10.1093/nsr/nwag440

**Published:** 2026-07-16

**Authors:** Zilong Li, Zili Ma, Pin Song, Junchi Xu, Zehua Liu, Yiming Zhang, Yuhe Zhu, Chuanjian Wang, Hao Yuan, Chao Gao, Federico Rosei, Yujie Xiong

**Affiliations:** Hefei National Research Center for Physical Sciences at the Microscale, School of Chemistry and Materials Science, University of Science and Technology of China, Hefei 230026, China; Hefei National Research Center for Physical Sciences at the Microscale, School of Chemistry and Materials Science, University of Science and Technology of China, Hefei 230026, China; Anhui Engineering Research Center of Carbon Neutrality, Key Laboratory of Functional Molecular Solids, Ministry of Education, College of Chemistry and Materials Science, Anhui Normal University, Wuhu 241000, China; Hefei National Research Center for Physical Sciences at the Microscale, School of Chemistry and Materials Science, University of Science and Technology of China, Hefei 230026, China; Hefei National Research Center for Physical Sciences at the Microscale, School of Chemistry and Materials Science, University of Science and Technology of China, Hefei 230026, China; Suzhou Institute for Advanced Research, University of Science and Technology of China, Suzhou 215123, China; Hefei National Research Center for Physical Sciences at the Microscale, School of Chemistry and Materials Science, University of Science and Technology of China, Hefei 230026, China; Hefei National Research Center for Physical Sciences at the Microscale, School of Chemistry and Materials Science, University of Science and Technology of China, Hefei 230026, China; Hefei National Research Center for Physical Sciences at the Microscale, School of Chemistry and Materials Science, University of Science and Technology of China, Hefei 230026, China; Anhui Engineering Research Center of Carbon Neutrality, Key Laboratory of Functional Molecular Solids, Ministry of Education, College of Chemistry and Materials Science, Anhui Normal University, Wuhu 241000, China; Hefei National Research Center for Physical Sciences at the Microscale, School of Chemistry and Materials Science, University of Science and Technology of China, Hefei 230026, China; Department of Chemical and Pharmaceutical Sciences, University of Trieste, Trieste 34127, Italy; Hefei National Research Center for Physical Sciences at the Microscale, School of Chemistry and Materials Science, University of Science and Technology of China, Hefei 230026, China; Anhui Engineering Research Center of Carbon Neutrality, Key Laboratory of Functional Molecular Solids, Ministry of Education, College of Chemistry and Materials Science, Anhui Normal University, Wuhu 241000, China; Suzhou Institute for Advanced Research, University of Science and Technology of China, Suzhou 215123, China

**Keywords:** photocatalysis, C−H activation, Lewis acid, chloromethane, flow tandem system

## Abstract

Methane (CH_4_), a key component of combustible ice, is a plentiful yet largely underutilized ocean resource. Its extreme inertness and the complexity of its storage and transportation have severely hindered its use. Here, we present a tandem flow system that produces freshwater through seawater desalination, while the resulting concentrated seawater is rich in chlorine ions and serves as a source for CH_4_ chlorination over a floatable photocatalyst with a three-phase interface. The key lies in tailoring the interfacial acid–base microenvironment and electronic structure of Pt–TiO_2_ photocatalyst to steer the radical processes toward selective methane chlorination. ·OH radicals mediate C–H activation to generate ·CH_3_ radicals, which subsequently couple efficiently with interfacial ·Cl radicals to yield CH_3_Cl. The introduction of trace Lewis acids (e.g. Al^3+^) further enhances selectivity by regulating the local proton balance, suppressing OH^−^ accumulation, and stabilizing key radical intermediates. Our system achieves 98.1% CH_3_Cl selectivity with production rates of 2.9 mmol g^−1^ h^−1^ in AlCl_3_ solution and 79.5 mmol m^−2^ h^−1^ under flow operation, demonstrating long-term stability over 15 days. This work establishes a general paradigm for controlling aqueous radical chemistry under mild conditions, and paves the way for the practical exploitation of offshore ocean resources.

## Teaser

By harnessing sunlight, seawater, and methane, a solar-driven tandem system sustainably co-produces freshwater and chloromethane, unlocking a green pathway for utilizing abundant offshore marine resources.

## INTRODUCTION

The sustainable exploitation of two abundant ocean resources, seawater and methane hydrates, represents a major opportunity to address critical global sustainability challenges. Methane (CH_4_), which is distributed across various reservoirs, including natural gas fields, shale gas, coal seam methane, biogas and combustible ice (methane hydrates), is a significant dual-use resource. It serves as a vital energy vector and a key chemical feedstock [1]. Methane hydrates, which are primarily found in marine sediments, are particularly important, as they are estimated to consist of 3–20 × 10^15^ m^3^ of CH_4_, thus surpassing the combined volume of all other fossil fuel reserves [2]. However, despite this colossal potential, the exploitation of these reserves is hindered by their remote locations and the transport challenges stemming from the low boiling point (111 K) of CH_4_, while industrial conversion of CH_4_ requires energy-intensive processes involving elevated temperatures and pressures to cleave robust C–H bonds (439 kJ mol^−1^) [3]. These constraints necessitate on-site processing approaches that enable direct conversion of methane at extraction sites under mild conditions [4].

**Figure 1.**
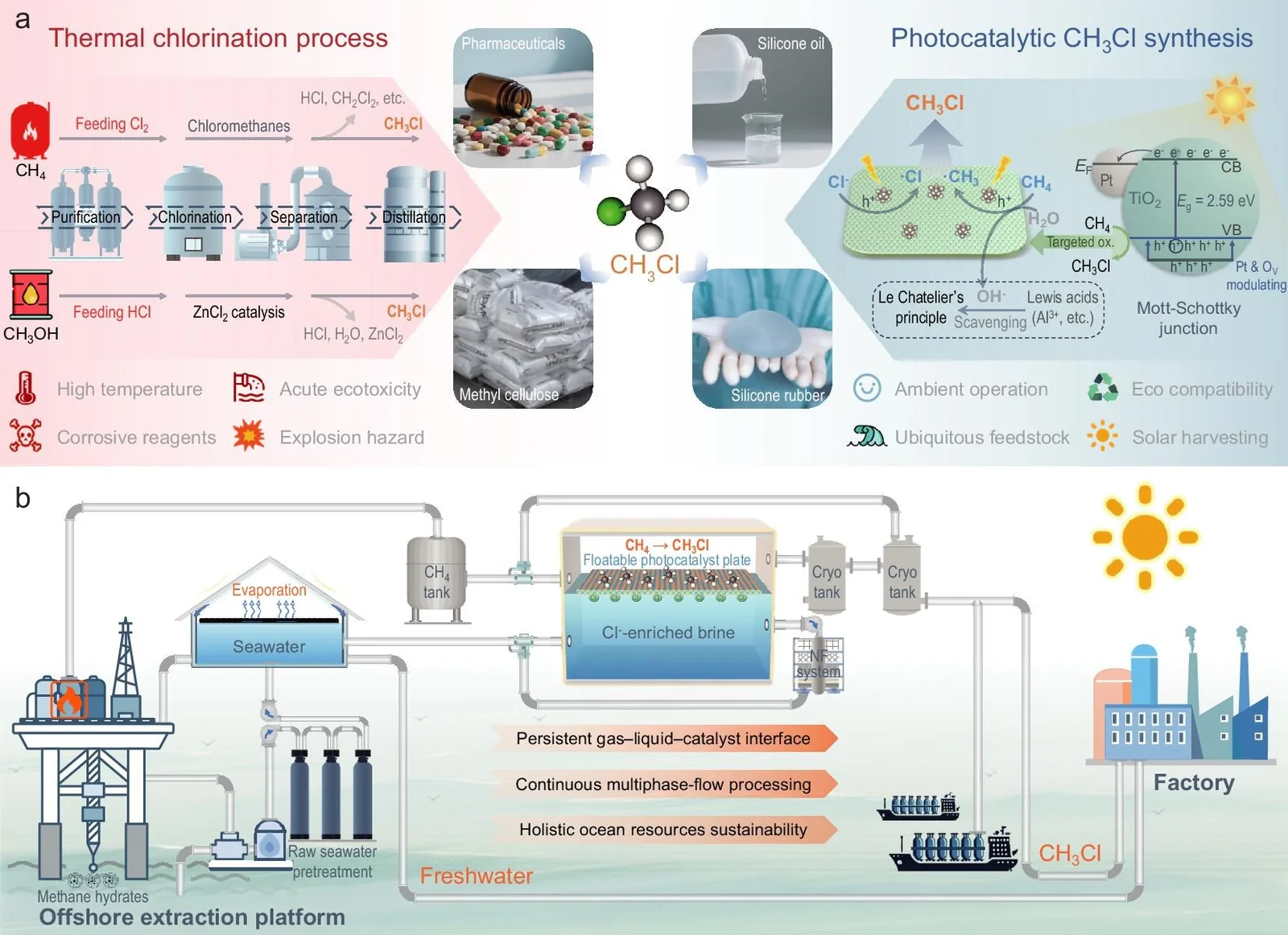
Conceptual design for methane chlorination in aqueous chloride media. (a) Comparison of the traditional industrial pathway and the proposed photocatalytic pathway for CH_4_ chlorination. (b) Schematic illustration of the tandem photothermal-photocatalytic system for photocatalytic CH_4_ chlorination at the three-phase catalytic interface by leveraging discarded brine from solar-driven desalination.

Chloromethane (CH_3_Cl) is a versatile industrial platform chemical that is widely used in the production of silicones, agrochemicals and pharmaceuticals. It can also be converted into higher-value products such as synthetic fuels, light olefins, and other key commodity chemicals [5–7]. In this context, converting CH_4_ to CH_3_Cl is a promising strategy for methane exploitation. This not only adds value, but also dramatically enhances process safety, a critical consideration for large-scale offshore operations. Compared to CH_4_, which is odorless, has a wide explosion range (5%‒15%) and requires hazardous cryogenic liquefaction, CH_3_Cl exhibits superior safety characteristics, including a higher ignition temperature (∼632 vs. ∼537°C), a narrower explosion limit (8.1%‒17.4%) and a distinct odor for immediate leak detection. Furthermore, CH_3_Cl’s higher boiling point (249 K) enables easier liquefaction under moderate pressure, substantially reducing the complexity and risks of infrastructure associated with storage and transport [8]. However, conventional industrial CH_4_ chlorination uses corrosive reagents (HCl or Cl_2_) and harsh conditions, thus underscoring the need for sustainable alternatives (Fig. 1a) [9].

Solar-driven photocatalysis can be used to drive efficient CH_4_ chlorination under ambient conditions, since energetic photogenerated charge carriers are capable of overcoming thermodynamic barriers [10]. Photocatalytic CH_4_ chlorination using chloride salts as a benign chlorinating agent shows significant promise. Further scaling of this technology requires abundant yet sustainable supplies of chlorine, a requirement that is uniquely fulfilled by seawater [11]. Considering the vast reserves of CH_4_ in oceanic methane hydrates, *on-site* photocatalytic conversion of CH_4_ using seawater as a chlorination medium is a viable approach (Fig. 1b). Despite notable advances, photocatalytic methane conversion remains constrained by modest yields (typically ≤366 μmol g^−1^ h^−1^; Table S1), largely due to poorly controlled radical evolution [5,6,12–15]. In aqueous media, coexisting hydroxyl (·OH) and chlorine (·Cl) radicals engage in competing oxidation and coupling pathways, often leading to unselective product mixtures or overoxidized byproducts instead of well-defined C–Cl coupling. Compounding this issue, the strongly polar hydrated surfaces prevalent under reaction conditions further destabilize key radical intermediates. As a result, achieving high selectivity remains a fundamental and persistent challenge in photocatalytic methane functionalization in aqueous media [16].

Here, we propose that rational control of the interfacial microenvironment can steer radical pathways toward selective chlorination [17]. Pt-decorated TiO_2_ creates a Mott‒Schottky junction [18] that enhances charge separation and tunes surface acidity to favor Cl^−^ activation and ·Cl generation. The introduction of trace Lewis acids (e.g. Al^3+^, Mg^2+^, Fe^3+^) further regulates the local proton balance, suppressing OH^−^ accumulation and stabilizing reactive intermediates. This dual modulation of acid–base and electronic structure confines ·CH_3_ and ·Cl radicals at the catalytic interface, establishing a surface-confined radical synergy wherein ·OH activates CH_4_ to ·CH_3_ and the latter rapidly couples with ·Cl to form CH_3_Cl. The engineered Pt(0.13)/TiO_2_ photocatalyst achieves a CH_3_Cl production rate of 2.9 mmol g^−1^ h^−1^ with 98.1% selectivity in AlCl_3_ solution, which is significantly superior to previous benchmarks (≤366 μmol g^−1^ h^−1^; Table S1). When integrated into a flow three-phase reactor using concentrated seawater derived from solar steam generation (SSG) as the chlorination source, the catalyst sustains a CH_3_Cl productivity of 79.5 mmol m^−2^ h^−1^ over 15 days of continuous operation, far surpassing previous reported systems (≤0.61 mmol m^−2^ h^−1^). This work establishes interfacial acid–base and electronic modulation as a general strategy for selective C–H functionalization in water under mild, sustainable conditions.

## RESULTS AND DISCUSSION

### Synthesis and characterization of the photocatalyst

To experimentally validate our premise that interfacial acid–base modulation and electronic tuning can steer aqueous radical pathways, we designed a model Pt–TiO_2_ photocatalyst for selective chlorination. Anatase TiO_2_ was selected as the foundational support material owing to its excellent physicochemical stability, strong photooxidative ability, and well-defined surface hydroxyl network that mediates radical formation [19]. We then incorporated ultrafine Pt nanoparticles (NPs) via impregnation to construct Pt(x)/TiO_2_ (x% refers to the weight percentage of loaded Pt) composites. The Pt cocatalyst offers multiple critical functions: it modulates acid–base properties, facilitates interfacial electron transfer, and stabilizes transient radical intermediates, thereby collectively optimizing the entire process of substrate adsorption, activation and transformation during CH_4_ chlorination [4,20].

**Figure 2.**
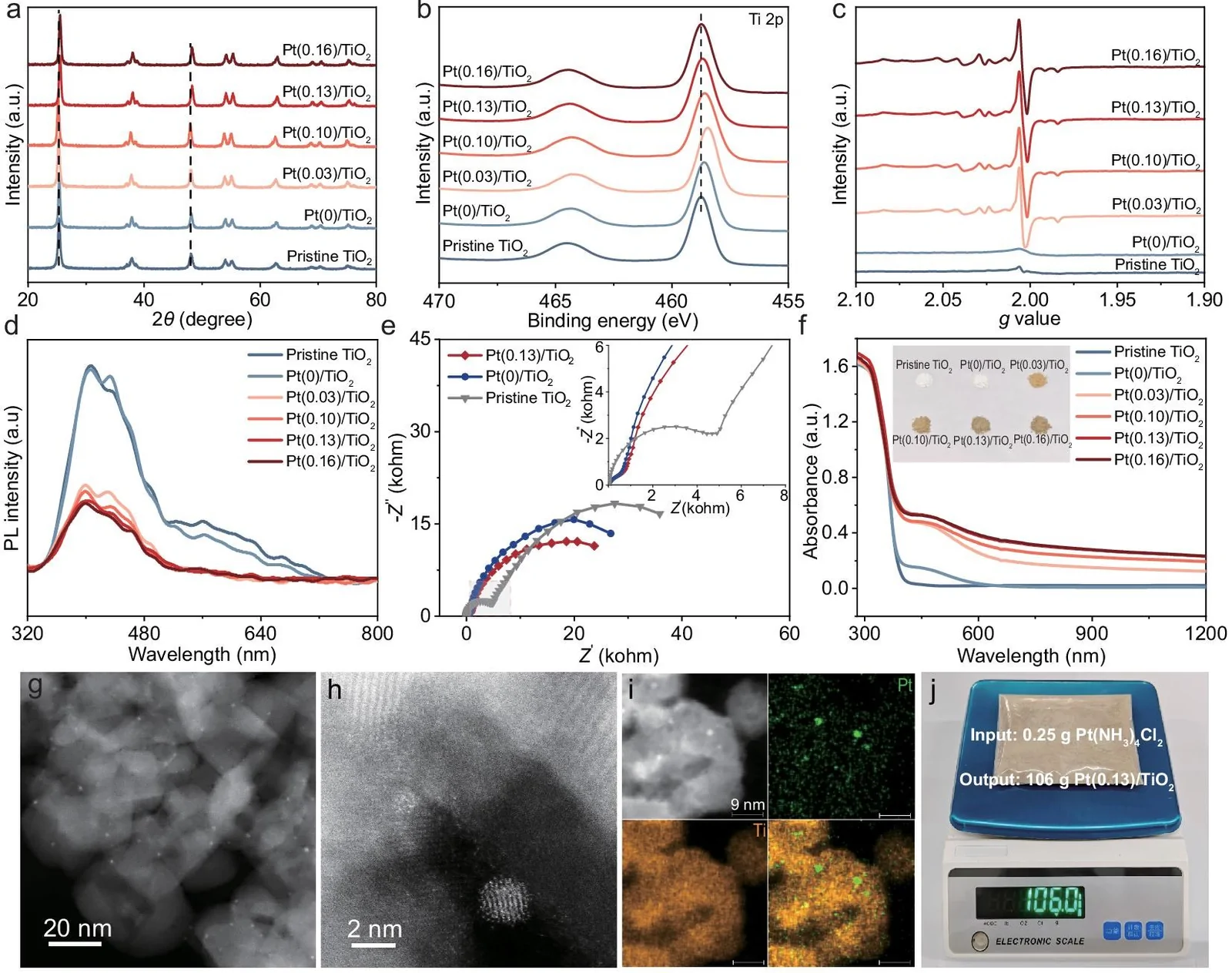
Structure and properties of the photocatalysts. (a) XRD, (b) high-resolution XPS spectra of Ti 2p and (c) EPR spectra of pristine TiO_2_ and Pt(x)/TiO_2_ (x% representing the nominal Pt weight loading percentage). (d) Steady-state photoluminescence emission spectra of pristine TiO_2_ and Pt(x)/TiO_2_. (e) Nyquist plots of pristine TiO_2_ and Pt(x)/TiO_2_ under AM 1.5G. (f) Ultraviolet‒visible diffuse reflectance spectra of pristine TiO_2_ and Pt(x)/TiO_2_. (g) HRTEM and (h) ACTEM dark-field images of Pt(0.13)/TiO_2_. (i) Elemental mapping images of Pt and Ti in Pt(0.13)/TiO_2_. (j) Photograph of Pt(0.13)/TiO_2_ photocatalyst obtained in the scale-up production. The raw material of one-pot synthesis includes 0.25 g of Pt(NH_3_)_4_Cl_2_·3H_2_O and appropriate TiO_2_ support.

Inductively coupled plasma atomic emission spectroscopy (ICP-AES) confirms that the actual Pt loading closely matches the nominal values, demonstrating the impregnation process’s effectiveness in achieving controlled Pt loadings (Table S2). Furthermore, the X-ray diffraction (XRD) pattern shows the absence of Pt-derived crystalline phases after Pt loading (Fig. 2a), indicating that Pt species are highly dispersed [21]. Concomitant shifts in the XRD peaks and broadening of the Raman peaks over Pt(0.13)/TiO_2_ (Fig. S1) suggest strong Pt–TiO_2_ interfacial interactions, which are critical for the formation of a Schottky barrier that mediates charge redistribution and surface acid–base equilibration [22].

X-ray photoelectron spectroscopy (XPS, Fig. S2) confirms the successful loading of Pt, with the coexistence of Pt^0^ and Pt^2+^ species. Additionally, H_2_ treatment induced the formation of Ti^3+^ and surface hydroxyl, as evidenced by the shift in the Ti 2p peak (Fig. 2b) and the X-ray absorption spectra (Fig. S3). An increase in the intensity of the Ti 2p peak at higher Pt loadings indicates a pronounced electron-withdrawing effect by the electronegative Pt species. This is correlated with local acidification and enhanced hydroxyl density at the interface [23]. Electron paramagnetic resonance (EPR) measurements (Fig. 2c) show that H_2_-treated TiO_2_ (i.e. Pt(0)/TiO_2_) generates abundant oxygen vacancies (*g* = 2.003), which may influence the adsorption properties and facilitate oxygen-related reactions [24]. The presence of additional EPR peaks in Pt-modified TiO_2_ suggests the existence of Ptᵟ^+^ sites and strong metal-support interactions, which are key to stabilizing ·CH_3_ and ·Cl radicals under photocatalytic conditions [25]. Thermogravimetric analysis (Fig. S4) reveals that Pt(0.13)/TiO_2_ exhibits superior thermal stability, highlighting its robustness under intense light irradiation.

The role of Pt in facilitating charge separation and regulating surface microenvironment was further examined by photoluminescence (PL) and photoelectrochemical measurements. Figure 2d shows a significant decrease in PL intensity following Pt modification, indicating suppressed recombination of photogenerated charge carriers. This behavior is consistent with the establishment of a Mott–Schottky junction at the Pt/TiO_2_ interface, which promotes directional carrier migration and facilitates charge separation. Electrochemical impedance spectroscopy measurements reveal that Pt(0.13)/TiO_2_ exhibits optimal charge transfer dynamics with minimal charge transfer resistance (Fig. 2e). Photocurrent responses and Mott–Schottky analysis further reveal that Pt and oxygen vacancies cooperatively enhance charge separation and shift the band energetics to more positive flat-band potential (Figs S5–S7), thereby increasing the oxidative driving force of photogenerated holes under illumination [26]. Ultraviolet‒visible diffuse reflectance spectroscopy (Fig. 2f) shows that H_2_ treatment enhances light absorption due to surface disorder, while Pt modification extends light absorption across the visible spectrum via the Schottky barrier that forms at the Pt‒TiO_2_ interface [27]. This increase in light absorption is apparent in the gradual deepening of color of the photocatalyst in the optical images as the Pt content increases. The band gap (*E*_g_) of Pt(0.13)/TiO_2_, as determined by the Tauc plots (Fig. S8) [28], narrows to 2.59 eV in contrast to pristine TiO_2_ (3.04 eV) and Pt(0)/TiO_2_ (3.02 eV). This reflects Pt-induced modification of the band structure. Ultraviolet photoelectron spectroscopy measurements confirm the reduced work function (3.93 vs. 4.00 eV) and valence band upshift (2.53 vs. 2.90 eV) of Pt(0.13)/TiO_2_, in contrast to pristine TiO_2_ (Fig. S9 and Table S3). The modified valence band position endows the photogenerated carriers with the appropriate oxidation potential (Fig. S10), which is essential for enhancing the selectivity of complex CH_4_ conversion [29].

Field emission scanning electron microscopy and transmission electron microscopy images (Figs S11 and 12) reveal uniform photocatalyst particles measuring ∼40 nm in size, with negligible morphological changes after Pt loading. Dark-field microscopy (Fig. 2g) confirms the uniform distribution of Pt NPs on TiO_2_ through high-contrast imaging. Aberration-corrected transmission electron microscopy (ACTEM, Figs 2h and S13) and high-resolution elemental mapping (Fig. 2i) show that the Pt NPs are ∼1–2 nm in size. Line-scanning electron energy loss spectra (Fig. S14) reveal a localized Ti L-edge shift near Pt, consistent with interfacial electronic restructuring that tunes surface acid–base character. *In situ* diffuse reflectance infrared Fourier transform spectroscopy (DRIFTS, Fig. S15) using CO as a probe identifies distinct chemisorption signatures at 2056 cm^−1^ assigned to the Pt NPs [30]. Nitrogen adsorption‒desorption analysis (Fig. S16) shows a slight reduction in surface area after Pt decoration, consistent with the embedding of Pt NPs within surface cavities [31]. The retained porosity ensures sufficient exposure of hydroxyl-rich sites for radical confinement. Moreover, we implemented a scale-up production process to validate the scalability of the Pt(0.13)/TiO_2_ catalysts. As illustrated in Figs 2j and S17, a single batch yielded up to 106 g at an estimated cost of ∼19 USD, demonstrating the practical feasibility of mass production.

### Investigation of photocatalytic CH_4_ chlorination

To evaluate the efficacy of our proposed interfacial strategy for steering aqueous radical reactions, photocatalytic CH_4_ chlorination using a series of chloride salts as benign chlorine sources was performed and optimized in batch reactors under ambient conditions. Bare TiO_2_ exhibited negligible CH_4_ conversion without any detectable H_2_ or C_2_H_6_. However, the incorporation of Pt significantly enhanced CH_4_ conversion activity, underscoring the essential role of the Pt cocatalyst in mediating charge transfer and radical initiation. In chloride-free systems, Pt/TiO_2_ produced C_2_H_6_ at 236 μmol g^−1^ h^−1^ via methyl radical recombination, indicating the abundant formation of ·CH_3_ on the catalyst surface.

**Figure 3.**
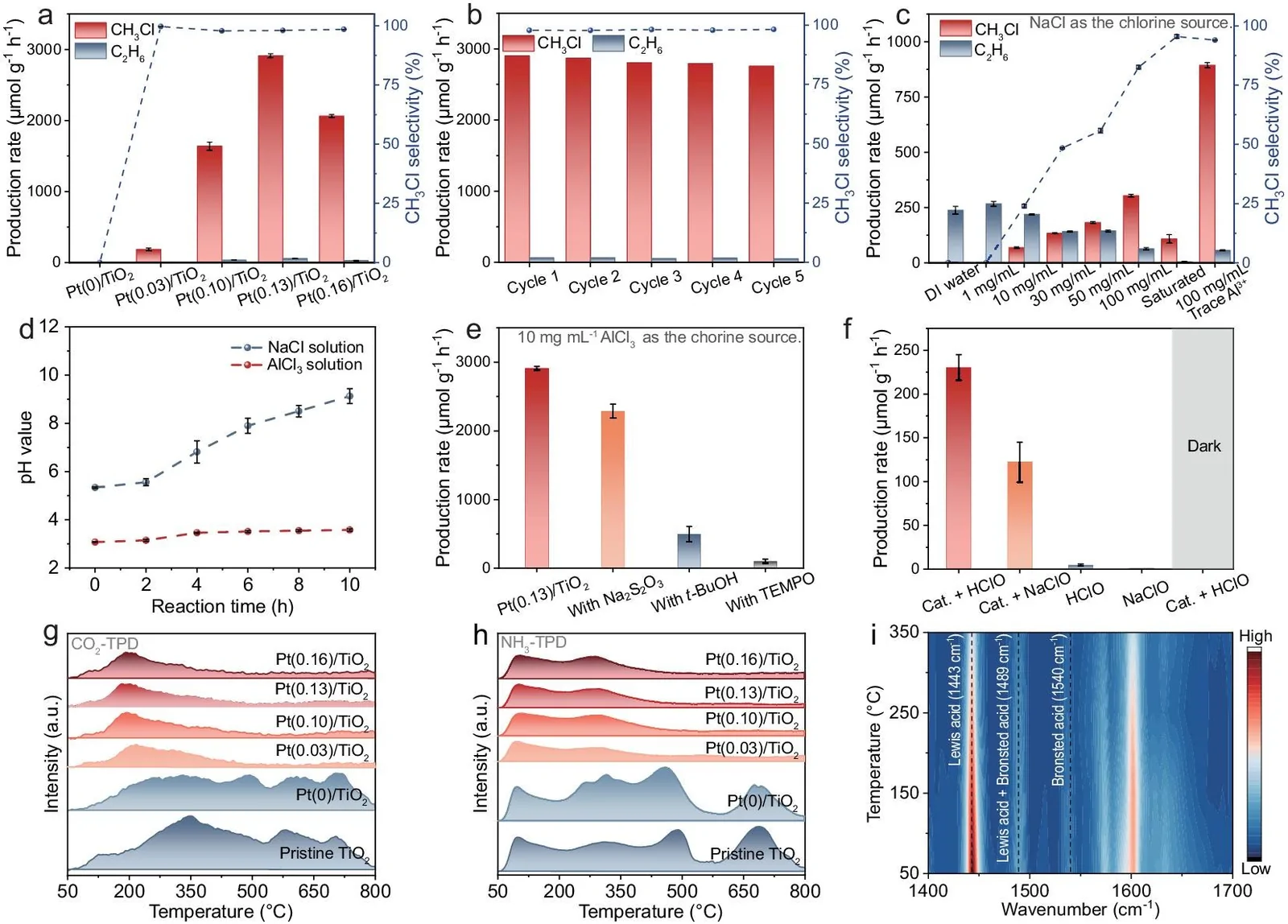
Performance of photocatalytic CH_4_ conversion. (a) Product yields and CH_3_Cl selectivity over Pt(x)/TiO_2_ photocatalyst in 10 mg mL^−1^ AlCl_3_⋅6H_2_O solution. (b) Cycling tests of photocatalytic conversion of CH_4_ over Pt(0.13)/TiO_2_. Product yields and CH_3_Cl selectivity over Pt(0.13)/TiO_2_ in (c) NaCl solutions of different concentrations and with trace Al^3+^ adding to 100 mg mL^−1^ NaCl solution as a control. (d) Variation in pH of solution with reaction time during photocatalytic CH_4_ chlorination over the Pt(0.13)/TiO_2_ in NaCl (100 mg mL^−1^) and AlCl_3_ (10 mg mL^−1^) solutions. (e) Production rates of CH_3_Cl in intermediate scavenging experiments using Na_2_S_2_O_3_ (5 mM, active chlorine scavenger), *t*-BuOH (5 mM, ·OH scavenger), and TEMPO (1 mM, ·CH_3_ scavenger). (f) Production rates of CH_3_Cl over Pt(0.13)/TiO_2_ with different active-chlorine as benign chlorine sources under different reaction conditions. (g) CO_2_-TPD and (h) NH_3_-TPD for pristine TiO_2_ and Pt(x)/TiO_2_. (i) Fourier transform infrared spectra over Pt(0.13)/TiO_2_ with pyridine as the probe molecule at the desorption temperature of 50, 150, 250, and 350°C, respectively.

Orthogonal tests were conducted to optimize Pt loading and chloride concentration for CH_3_Cl production. As shown in Fig. 3a, the CH_3_Cl yield exhibits a volcano-shaped trend with increasing Pt loading, likely due to balanced light absorption and shielding effect [32]. Pt(0.13)/TiO_2_ exhibited the highest CH_3_Cl production rate (2912 μmol g^−1^ h^−1^), together with a remarkable selectivity of 98.1%. Gas chromatography and ^1^H nuclear magnetic resonance confirmed dominant CH_3_Cl production and suppressed overoxidation (Fig. S18) [33]. Lewis acid concentration also plays a critical role in governing both activity and selectivity. The optimal AlCl_3_⋅6H_2_O concentration was determined to be 10 mg mL^−1^ (Fig. S19), as this maximized the catalyst‒substrate interaction. Higher concentrations, however, led to reduced CH_3_Cl yields due to excessive Al(OH)_3_ formation blocking the active sites. Cycling stability tests under optimized conditions (i.e. 10 mg mL^−1^ AlCl_3_⋅6H_2_O) revealed that Pt(0.13)/TiO_2_ retained 89% of the initial CH_3_Cl yield after five cycles, demonstrating consistently high selectivity (Fig. 3b). Furthermore, morphological and structural characterizations (Figs S20–S23) of the photocatalyst following cycling performance tests revealed no discernible change. ICP-AES analysis identified the retention of Pt content, with only minimal accumulation of Al (0.356 wt%; Table S2) in the form of Al(OH)_3_ on the surface. These findings collectively underscore the photocatalyst’s exceptional stability. Additionally, isotopic labelling experiments (Fig. S24) provided direct evidence of CH_4_ chlorination in chloride solutions [34].

To investigate the impact of chloride sources on the production of CH_3_Cl, a systematic evaluation of various alkali, alkaline-earth and transition-metal chlorides was conducted. This cation screening revealed a critical dependence on Lewis acidity: alkali-metal chlorides generally exhibited a relatively inferior production rate and selectivity for CH_4_ chlorination to CH_3_Cl (Figs S25–S27), whereas alkaline-earth-metal chlorides significantly improved performance. Adding trace Al^3+^ to a 100 mg mL^−1^ NaCl solution enhanced the CH_3_Cl production rate from 303 to ∼900 μmol g^−1^ h^−1^ with 94% selectivity, demonstrating the ability of Lewis-acidic species to regulate interfacial reactivity. The superior CH_3_Cl production rate (485 μmol g^−1^ h^−1^) in the MgCl_2_ solution is probably due to the strong Lewis acidity of Mg^2+^ and the limited solubility of its hydroxide Mg(OH)_2_. Among the transition metal chlorides, an impressive CH_3_Cl yield of 2141 μmol g^−1^ h^−1^ was achieved in the FeCl_3_ solution, accompanied by a selectivity of 97.3%. These results emphasize the important role of cation species in CH_4_ chlorination, with the chemical softness of the cations, particularly their Lewis acidity, being a key factor in determining the efficiency and selectivity of interfacial radical coupling [35].

To determine whether the Lewis-acid-driven promotion of CH_3_Cl formation is a general phenomenon, we extended our study to diverse catalytic platforms. A series of representative photocatalysts for CH_4_ activation were evaluated, comprising various oxide supports (anatase TiO_2_, rutile TiO_2_, ZnO, and Co_3_O_4_) and metal cocatalysts (Pt, Pd, Ru, and Ni). As shown in Figs S28 and S29, CH_3_Cl formation was highly dependent on catalyst composition, underscoring the sensitivity of CH_4_ chlorination to interfacial properties. While Pt/anatase TiO_2_ delivered the highest activity, other oxide supports, as well as TiO_2_ loaded with Ru or Ni, were substantially less effective, with Pd/TiO_2_ showing moderate performance. These variations align well with the distinct optical absorption, band alignment and C–H activation capability of the respective catalysts. Remarkably, the introduction of AlCl_3_ induced a pronounced and consistent enhancement in CH_3_Cl formation across all tested systems. The production rates surged to ∼2912 μmol g^−1^ h^−1^ on Pt/anatase TiO_2_ and ∼1534 μmol g^−1^ h^−1^ on Pd/TiO_2_. Despite the distinct intrinsic activities of these materials, this universal enhancement confirms that Lewis-acid assistance broadly and fundamentally modulates the interfacial reaction environment to drive CH_4_ chlorination.

The correlation between Lewis acidity, local pH stability and radical selectivity was further investigated. As shown in Fig. 3d, the pH of the NaCl solution continuously increased during photocatalytic CH_4_ conversion, which is consistent with hydroxide accumulation resulting from ·OH generation. In contrast, the AlCl_3_ system exhibited a nearly constant pH value due to Al^3+^ hydrolysis and the sustained acidity of its conjugate aqua complexes. This self-buffering effect preserves a mildly acidic interface, thereby stabilizing reactive intermediates and promoting the coupling of ·CH_3_ and ·Cl. Radical-scavenging experiments confirmed that CH_4_ chlorination proceeds via a radical synergy mechanism (Fig. 3e), as the addition of *t*-BuOH (·OH scavenger) or TEMPO (·CH_3_ scavenger) almost completely suppressed CH_3_Cl formation, while Na_2_S_2_O_3_ (scavenger of active-chlorine species) caused only partial inhibition. These results indicate that ·OH radicals are primarily responsible for C–H activation, while surface-confined ·Cl species drive selective C–Cl coupling. Although active-chlorine species (HClO, NaClO) can yield CH_3_Cl under illumination (Fig. 3f), their contribution is minor compared to chloride-mediated photocatalysis. HClO may additionally undergo limited direct photolysis to generate ·Cl and ·OH radicals under illumination. However, this contribution remains weak under the present Xe-lamp irradiation conditions (4.7 μmol g^−1^ h^−1^). The significantly reduced activity in the absence of Cl^−^ and the negligible conversion in the dark indicate that CH_4_ activation and chlorination proceed mainly through surface-confined radical pathways rather than homogeneous active-chlorine photochemistry.

To decouple the effect of solution acidity, control experiments were conducted in HCl/NaCl mixed electrolytes (Fig. S30). The CH_3_Cl productivity displayed a volcano-type dependence on pH, peaking at pH 3. Overly acidic conditions suppress ·OH formation and C–H activation, while alkaline conditions favor unselective C–C coupling. These findings collectively reveal that while bulk pH control can modulate the balance between activation and coupling processes, it falls short of explaining the dramatic enhancement induced by Lewis acids. Rather, our results demonstrate that a dynamically buffered, Lewis-acid-regulated local microenvironment is critical. This distinct regulation optimizes the synergy between ·OH-driven activation and ·CH_3_–·Cl coupling, ultimately enabling high selectivity in aqueous methane chlorination.

To further explore the photocatalyst’s surface reactivity, its acidic and basic properties were characterized via temperature-programmed desorption (TPD). As illustrated in Fig. 3g and h, both pristine TiO_2_ and Pt(0)/TiO_2_ exhibit a variety of acidic and basic sites. However, their pronounced non-specific adsorption hinders their reactivity and selectivity. Loading Pt species suppressed strongly acidic and basic sites while preserving moderate sites, as evidenced by desorption signals observed at medium temperatures (50–400°C). According to the Sabatier principle, the adsorption strength of the moderate sites is in the optimal range, enabling effective reactant adsorption while allowing facile product desorption. During CH_4_ chlorination, basic sites were associated with the adsorption of weakly acidic CH_4_, as confirmed by CH_4_-TPD (Fig. S31). Conversely, Cl^−^ ions, which act as Lewis bases, are preferentially adsorbed at acidic sites. The incorporation of Pt species modulated the acidity and basicity of TiO_2_, thereby endowing the Pt(x)/TiO_2_ photocatalyst with a balanced acidic and basic nature that facilitates moderate adsorption, consistent with the Sabatier principle [36,37].

The acid strength and types were further evaluated using Fourier transform infrared spectroscopy, using pyridine as a probe molecule. The results revealed that Lewis acidity (1443 cm^−1^) predominates in Pt(0.13)/TiO_2_, with minor Brønsted acidity (1540 cm^−1^) attributed to surface hydroxylation (Fig. 3i). Additional signals at 1489 cm^−1^ indicate a synergistic interaction between Lewis and Brønsted sites, which is consistent with the radical synergy pathway [38]. The substantial acidity maintained up to 350°C (Table S4) confirms that Pt modification facilitates optimal substrate adsorption and simultaneously increase the stability and concentration of acidic/basic sites on the photocatalyst. Together, these results demonstrate that a self-regulating interface at Pt–TiO_2_, synergistically modulated by Lewis-acidic cations, orchestrates radical confinement and enables precise control over aqueous C–H functionalization [39]. This interfacial microenvironment regulation holds broader significance for catalytic transformations in which local adsorption energetics, intermediate stabilization and proton/charge transfer collectively govern reactivity and selectivity.

### Mechanistic elucidation of CH_4_ chlorination

**Figure 4.**
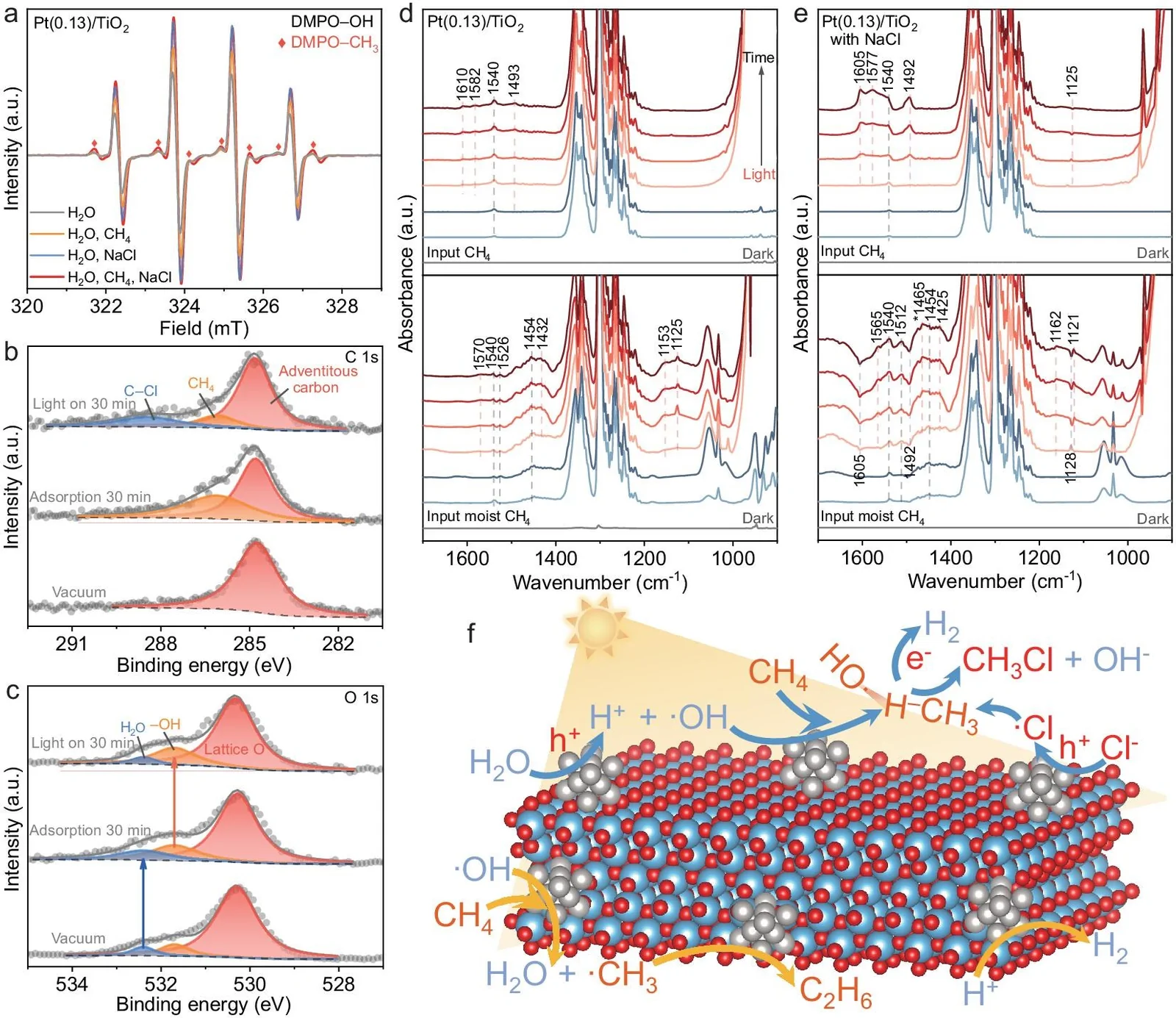
Mechanism investigation of CH_4_ conversion. (a) EPR spin-trapping spectra of active species formed during the photocatalytic reactions over Pt(0.13)/TiO_2_ in different solutions. DMPO was added as the radical trapping agent. *In situ* high-resolution XPS spectra of (b) C 1s and (c) O 1s for the Pt(0.13)/TiO_2_ photocatalyst under different conditions. (d, e) *In situ* DRIFTS spectra for photocatalytic CH_4_ conversion over Pt(0.13)/TiO_2_ under different conditions. (f) Schematic illustration of the proposed photocatalytic mechanism for CH_4_ chlorination over Pt/TiO_2_.

To elucidate the reaction mechanism of CH_4_ chlorination, EPR spin-trapping using 5,5-dimethyl-1-pyrroline N-oxide (DMPO) was conducted initially to monitor radical intermediates (Fig. 4a). The results confirmed the generation of hydroxyl radicals (·OH) by the Pt(0.13)/TiO_2_ photocatalyst in H_2_O under illumination. The ·OH quartet signals intensified upon the introduction of CH_4_, indicating the pivotal role of ·OH in CH_4_ activation [40]. In the NaCl solution, the ·OH signals intensified further and reached a maximum in the presence of both CH_4_ and NaCl, accompanied by the emergence of distinct sextet ·CH_3_ features. This synergistic enhancement suggests that chloride ions facilitate ·CH_3_ generation by modulating ·OH reactivity and stabilizing the radical intermediates. The simultaneous detection of ·OH and ·CH_3_ species thus supports a surface-confined radical synergy mechanism, wherein ·OH initiates C–H activation and ·Cl participates in the subsequent coupling step, in agreement with the radical-scavenging results and the proposed interfacial reaction network.

*In situ* near ambient pressure XPS (NAP-XPS) can be used to further decipher the mechanism. Deconvoluted C 1s spectra (Fig. 4b) revealed the appearance of CH_4_-derived signals when premoistened CH_4_ was introduced into the NAP-XPS chamber. Meanwhile, O 1s spectra (Fig. 4c) demonstrated increased H_2_O and –OH signals. In the absence of light, the Cl 2p and Ti 2p spectra remained unchanged, indicating that CH_4_ chlorination does not proceed spontaneously. Upon illumination, the Cl 2p peaks shifted to higher binding energies (Fig. S32), evidencing the generation of transient surface-bound Cl radicals [7], accompanied by a corresponding positive shift of Ti 2p peaks. Meanwhile, deconvoluted C 1s spectra revealed the transformation of CH_4_-derived C–H signals into C–Cl bond signals after illumination, providing direct evidence of CH_3_Cl formation [41]. Crucially, systems containing NaCl exhibited a signal for hydroxyl-containing species (i.e. –OH, ·OH and OH^−^) in O 1s spectra due to surface hydroxylation (Fig. 4c), whereas this signal was absent in NaCl-free controls (Fig. S33). These findings underscore the synergy between surface hydroxylation and chloride activation that sustains radical generation and enables high selectivity in C–H chlorination.

Furthermore, *in situ* DRIFTS measurements were performed to identify critical intermediates during the reaction. As shown in Fig. 4d and e, characteristic peaks for CH_4_ adsorption on the photocatalyst were evident under all conditions, including asymmetric (1200–1400 cm^−1^) and symmetric (1540 cm^−1^) C–H deformation vibrations [42]. The presence of H_2_O vapor induces additional C–H vibrations (1010–1080 cm^−1^) via hydrogen bonding networks. Illumination-induced peaks at 1022, 1493, 1582 and 1610 cm^−1^ suggest the formation of CH_3_/CH_2_ groups (Fig. 4d), thereby confirming the ability of Pt(0.13)/TiO_2_ to promote CH_4_ activation [7]. The incorporation of moist CH_4_ displays the O–CH_3_ modes (1125 and 1153 cm^−1^), thereby emphasizing the role of H_2_O in CH_4_ activation [43]. Distinct vibrational signals (1432, 1454, 1526, and 1570 cm^−1^) associated with CH_3_/CH_2_ groups were observed, thus confirming the C–H bond stretching induced by –OH. Adding NaCl significantly enhanced and redshifts the CH_3_/CH_2_ peaks (Fig. 4e), signifying Cl^−^-mediated stabilization of the activated methyl intermediates. Although the low-wavenumber C–Cl signals were obscured by additional light interference, the emerging CH_3_ signals (1162 and 1465 cm^−1^) provide direct evidence of CH_3_Cl production. Additionally, the presence of distinct downward signals assigned to C–H (1492 and 1605 cm^−1^) further confirmed the occurrence of CH_4_ chlorination. By integrating insights from photocatalytic experiments and *in situ* spectroscopy, we propose a surface-confined radical synergy mechanism for CH_4_ chlorination over Pt(0.13)/TiO_2_ in aqueous chloride solutions (Fig. 4f). Upon illumination, photoexcited holes oxidize interfacial H_2_O to generate OH radicals, while photogenerated electrons reduce H^+^ to form H_2_ [44]. Simultaneously, chloride ions are oxidized to surface-confined Cl radicals, a process facilitated by Lewis-acid activation (e.g. Al^3+^) that lowers the Cl^−^ oxidation barrier and stabilizes the interfacial proton balance [45]. Crucially, OH radicals attack CH_4_ molecules, weakening the C–H bond to allow coupling with Cl radicals, finally yielding CH_3_Cl, H_2_ and OH^−^ [46].

Maintaining a stable interfacial acid–base environment is essential for sustaining this radical network. An excessive OH^−^ accumulation inhibits CH_3_Cl formation by shifting the equilibrium, as predicted by Le Chatelier’s principle, and competes with Cl^−^ for activating on active sites [47]. Complexation of OH^−^ by Lewis acids has been demonstrated to mitigate this inhibition, preserving the mildly acidic interface necessary for selective radical coupling. In a parallel pathway, minor CH_3_ radicals undergo self-coupling to produce C_2_H_6_, highlighting the inherent complexity of this radical-mediated chlorination process. Nevertheless, due to the high local concentration of Cl radicals relative to CH_3_ radicals, the two encounter each other more readily than CH_3_ radicals do with themselves, leading to the rapid formation of CH_3_Cl. Furthermore, efficient chain propagation sustains this chlorination process and minimizes termination pathways, thereby limiting the formation of the C_2_H_6_ byproduct. The synergistic effect of Pt-mediated electronic modulation and Lewis-acid-induced buffering effectively stabilizes the interfacial radical network and suppresses termination pathways, thereby achieving a remarkable 98.1% selectivity for CH_3_Cl with negligible C_2_ byproducts (C_2_H_6_ and C_2_H_5_Cl, <2%).

### Validation in a solar-powered sustainable tandem system

**Figure 5.**
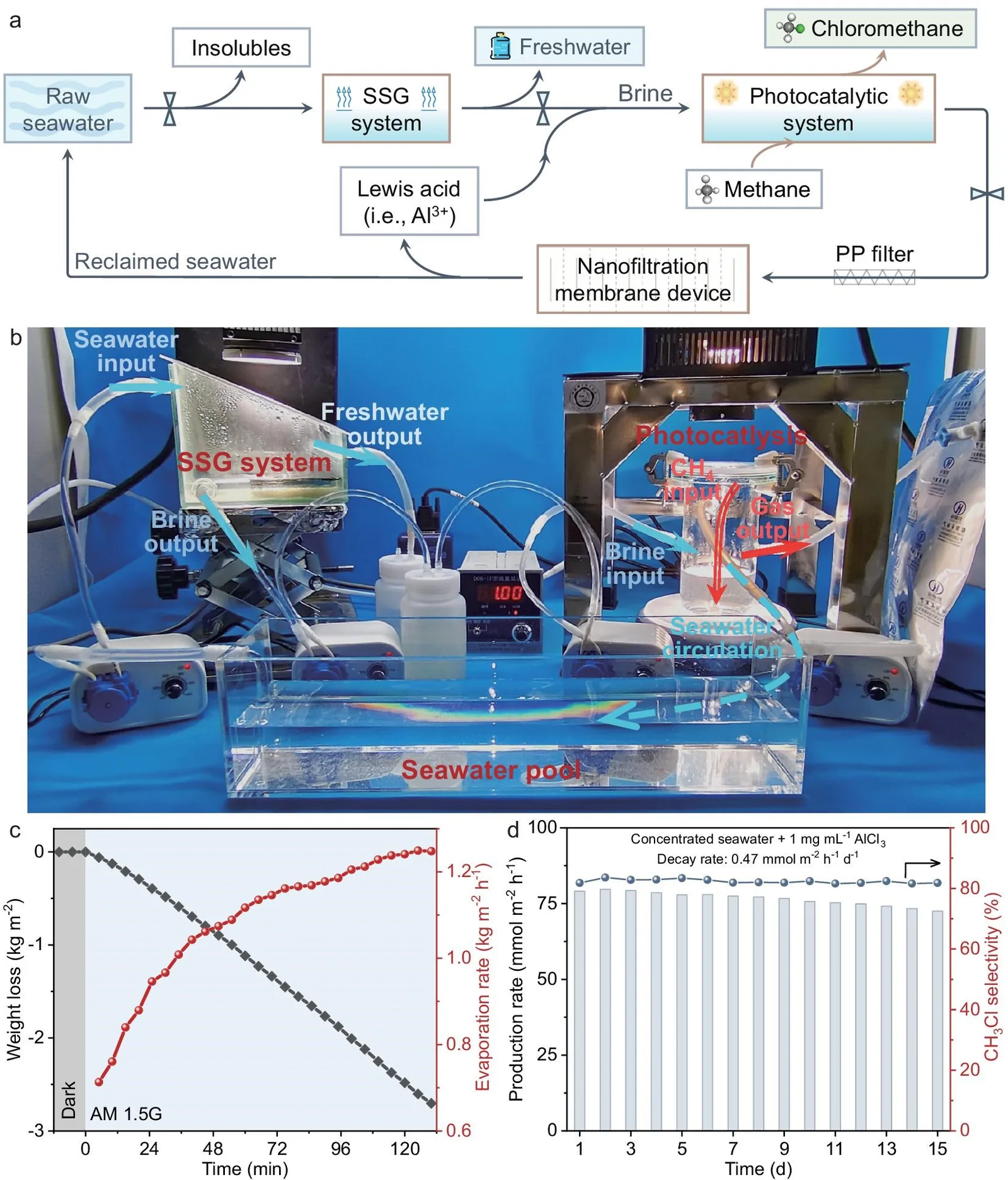
Construction of the flow tandem system. (a) Process flow diagram of a sustainable system for freshwater production and photocatalytic methane chlorination utilizing marine resources. (b) Photograph of the integrated flow tandem system comprising a solar steam generator and a photocatalytic CH_4_ conversion flow reactor. (c) Time-dependent seawater evaporation performance using the PPy–PVDF membrane under one sun irradiation (AM 1.5G), and the corresponding evaporation rate. (d) Long-term stability test of product yields and CH_3_Cl selectivity over Pt(0.13)/TiO_2_@fiberglass floatable plate in the flow reactor, with CH_4_ flow rate at 1.0 mL min^−1^ and brine flow rate at 2.0 mL min^−1^, respectively.

To assess the practical potential and robustness of the interfacial radical management mechanism, we operationalized the process within a designed integrated tandem system that couples solar-driven seawater desalination with photocatalytic CH_4_ chlorination (Fig. 5a). This system serves as a demanding testbed, wherein the photothermal SSG module first concentrates seawater, providing chloride-enriched brine that challenges the photocatalytic unit with a complex, real-world reaction medium. As illustrated in Figs 5b, S34 and S35, a floating PPy–PVDF photothermal membrane was fabricated to drive seawater evaporation [48], with condensed vapor yielding freshwater. The concentrated brine resulting from the SSG process can be directly used as feedstock for CH_4_ chlorination, creating a synergistic desalination–chemical synthesis loop. PPy functionalization enhances light absorption, raising the temperature of the air–seawater interface to 57°C under one-sun illumination (Figs S36–39) and achieving an evaporation rate of 1.25 kg m^−2^ h^−1^ (Fig. 5c). This is comparable to the high performance of previously reported SSG systems [48], demonstrating the reliability of the photothermal platform for tandem operation.

Reusing concentrated brine significantly improved the efficiency for photocatalytic CH_4_ chlorination. Seawater is inherently complex due to its diverse ions and organic impurities that could poison the active sites of catalysts [49], yet the original seawater (13.4 g/L Cl^−^) produced a CH_3_Cl yield of 51.6 μmol g^−1^ h^−1^ with 74.2% selectivity in the batch reactor (Fig. S40). In contrast, saturated brine (89.5 g/L Cl^−^, obtained by recovering ∼85% v/v freshwater) increased the production rate to 127 μmol g^−1^ h^−1^ with 81.0% selectivity. Moreover, seawater samples collected from different marine regions exhibited highly consistent performance (Fig. S41), indicating good tolerance toward natural seawater variability. The slightly higher activity observed for artificial seawater further suggests that trace impurities or naturally occurring reductive species in real seawater may partially consume reactive radical intermediates and modestly suppress CH_4_ chlorination efficiency. This highlights the need for catalyst and interface designs that remain resilient to the chemical complexity of natural seawater. To enable durable offshore operation, we immobilized the Pt(0.13)/TiO_2_ photocatalyst on a fiberglass membrane pretreated with amphiphilic Nafion resin. The resulting Pt(0.13)/TiO_2_@fiberglass floatable plate establishes a persistent gas‒liquid‒solid reaction interface that is suitable for continuous flow systems [50]. For stable reactor operation, ∼50% saturated brine (∼44.8 g/L Cl^−^) was employed to prevent salt crystallization on the floatable plate (Fig. S42), while the selected flow conditions ensured sufficient reactant supply and effective three-phase mass transfer without compromising interfacial stability.

The flow reactor demonstrated exceptional durability for CH_4_ chlorination during continuous operation of over 54 h (Fig. S43), sustaining a CH_3_Cl production rate of 59.7 mmol m^−2^ h^−1^ with 79.2% selectivity. Even after nine months of ambient storage, additional tests showed that the performance of the floatable plate had decayed by only ∼5%, with negligible changes in production rate and selectivity. Adding trace amounts of a Lewis acid (AlCl_3_·6H_2_O, 1 mg mL^−1^) further increased the CH_3_Cl production rate to 79.5 mmol m^−2^ h^−1^, with 82.8% selectivity. This highlights the positive effect of interfacial acid–base modulation. In contrast to batch reactor processes, the lower selectivity observed under continuous-flow operation is primarily associated with dissolved O_2_, which promotes competing overoxidation pathways and CO_2_ formation. This interpretation is further supported by control experiments using CH_4_-, air- and O_2_-saturated reaction solutions (Fig. S44), where the removal of dissolved O_2_ markedly suppressed CO_2_ generation and restored the CH_3_Cl selectivity to above 95%. Such oxygen-induced overoxidation is expected to be readily mitigated in future closed-loop and industrially optimized reactor systems through continuous CH_4_ atmosphere protection and minimized air exposure during recirculation.

Further long-term stability testing demonstrated exceptional durability (Fig. 5d). The device sustained 15 days of continuous operation with a slight decay rate of 0.47 mmol m^−2^ h^−1^ d^−1^ in production, highlighting its potential for practical applications. The minor activity decay is likely associated with the gradual deterioration of the Nafion-based three-phase interface under continuous illumination, which may slightly reduce gas–liquid–solid contact efficiency over extended operation. This suggests that further optimization of interfacial robustness could provide additional improvements in long-term stability. Over 98% of the added Al^3+^ was intercepted and recovered via a nanofiltration system (Fig. S45 and Table S5), without affecting the concentration of NaCl. Effective mass transfer at the three-phase interface ensured consistent and efficient CH_3_Cl production, highlighting the intimate coupling between macroscopic transport processes and the local interfacial environments that govern radical reactivity and selectivity in multiphase photocatalytic systems [50]. This successful translation from mechanistic insight to operational process underscores the potential of interfacial control strategies for sustainable solar-driven chemistry. Compared with alternative CH_4_ chlorination strategies (Table S6), the present photocatalytic system combines high selectivity with exceptional long-term operational stability, representing a distinctive strength of solar-driven catalysis. Although photocatalytic systems remain inherently limited in productivity relative to electrocatalytic counterparts, their direct utilization of solar energy without continuous electrical input provides a complementary pathway for CH_4_ valorization. These findings highlight the unique potential of interfacial photocatalysis for sustainable methane conversion under practically relevant operating conditions.

## CONCLUSIONS

We have presented a photocatalytic approach to the direct conversion of methane to chloromethane under ambient conditions. This approach uses readily available chloride salts as a source of chlorine and is based on the management of interfacial radical intermediates. Through synergistic electronic and acid–base engineering, this approach enables efficient methane chlorination under mild conditions by steering transient ·OH and ·Cl radicals toward a surface-confined radical synergy, where ·OH mediates C–H activation and adjacent ·Cl enables rapid ·CH_3_–·Cl coupling. The introduction of trace Lewis acids such as Al^3+^ plays a critical role in dynamically regulating interfacial proton balance, which suppresses hydroxide accumulation and stabilizes key radical intermediates, thereby enforcing high selectivity. This design achieves a CH_3_Cl production rate of 2.9 mmol g^−1^ h^−1^ with 98.1% selectivity in AlCl_3_ solutions. Remarkably, the CH_3_Cl yield remains at 0.3 mmol g^−1^ h^−1^ even in NaCl solutions, and can be further enhanced to ∼0.9 mmol g^−1^ h^−1^ by introducing trace amounts of Al^3+^. When implemented in a flow reactor using concentrated brine from solar steam generation, this solar-driven tandem system enables the sustainable, co-production of freshwater (1.25 kg m^−2^ h^−1^) and CH_3_Cl (79.5 mmol m^−2^ h^−1^) with exceptional durability (over 15 days). This solar-driven tandem system paves the way for a promising strategy that concurrently produces freshwater and transforms CH_4_ into a value-added product, establishing a transformative paradigm for the sustainable use of ocean resources. Fundamentally, these results highlight that precise control of interfacial acid–base equilibria and electronic structure can tame the intrinsic complexity of aqueous radical chemistry, providing a general blueprint for controlled C–H functionalization in water.

Looking forward, the conceptual framework of interfacial radical control introduced here could inspire catalytic interfaces for a broader set of aqueous radical transformations, including halogenation, oxygenation or amination. The CH_3_Cl produced in this process may further serve as a platform for downstream conversion to higher-value products (e.g. methanol, organosilanes, or fine chemicals), enabling a closed methane-to-chemicals cycle. Scaling up this technology toward pilot-scale and offshore deployment will require continued optimization of reactor architecture and flow management to maximize three-phase interfacial contact, improve mass transfer and minimize oxygen intrusion during recirculation. Strategies such as modular planar reactor configurations, directional flow-field engineering and distributed gas–liquid transport design may provide promising pathways toward efficient large-scale implementation by enhancing reactant utilization and maintaining favorable interfacial environments for selective radical chemistry. Further development of robust and scalable three-phase interfacial configurations, capable of maintaining efficient mass transfer and catalytic activity in the presence of salt accumulation, fouling and seawater impurities, will also be essential for long-term operation under realistic marine conditions. Integrating efficient solar harvesting with the valorization of stranded carbon resources has the potential to make this approach a cornerstone of the offshore circular economy, contributing directly to climate change mitigation and sustainable resource utilization. Furthermore, by combining fundamental radical chemistry with scalable photochemical systems, this work establishes a molecular basis for sustainable catalysis, connecting sunlight, seawater and methane. This paves the way for environmentally friendly synthetic methodologies.

## METHODS

### Synthesis of Pt(x)/TiO_2_ photocatalyst

Hydrophilic TiO_2_ NPs were used as light-absorbing support materials, and Pt as cocatalyst was decorated by an impregnation method. Firstly, Pt(NH_3_)_4_Cl_2_ was dissolved in water at a concentration of 1 mg mL^−1^ as the impregnation solution. In a typical procedure, 2.0 g of TiO_2_ were immersed in dilute impregnation solution (10.0 mL) containing the appropriate amount of Pt(NH_3_)_4_Cl_2_. The slurry was stirred for 12 h at room temperature and dried in an oil bath at 80°C. Subsequently, the resulting solids were fully ground and then reduced with H_2_ (10% in Ar) at 773 K for 5 h. The obtained photocatalysts were denoted as Pt(x)/TiO_2_ (x% representing the nominal metallic Pt weight loading percentage).

### Construction of the tandem flow system

The tandem system was assembled using a series of custom-designed glass components. Specifically, the solar steam generator (SSG) condenser and the photocatalytic reactor were constructed from quartz to ensure optimal light transmission. The system’s liquid circulation was managed by four peristaltic pumps (Changsha SIPULIN Co., Ltd.), with a flow rate set to 2.0 mL min^−1^. A precision mass flowmeter (Seven Star, D08-1F) controlled the CH_4_ flow, which was set at 1.0 mL min^−1^. Both the SSG device and the photocatalytic flow reactor had a total volume of 500 mL each. During the experiment, the liquid volume in the photocatalytic reactor was 150 mL, while the gas volume was 350 mL. The photocatalytic flow reactor featured a detachable quartz light window and four branch tubes. Two of the tubes extended from the upper part to the bottom of the reactor, while the other two were located at the middle section of the reactor to facilitate mass transfer and gas distribution. The system was irradiated by a 300 W xenon lamp (PLS-SXE300, Perfect Light), with a light intensity of 100 mW cm^−2^ (one sun) for the SSG system. For the photocatalytic flow reactor, the light intensity was set to 1.6 W cm^−2^ to optimize the reaction conditions. To prepare the floatable plate for catalyst immobilization, a pre-cleaned fiberglass membrane (47 mm diameter) was treated with amphiphilic Nafion resin. The Nafion solution, with a concentration of 0.5 wt%, was applied at 0.2 mL cm^−2^. The catalyst ink was prepared by dispersing 10.0 mg of composite photocatalyst powder into 1950 µL of ethanol, followed by mixing with 50 µL of a 5 wt% Nafion solution. The mixture was sonicated for 30 min to form a homogeneous ink, which was then uniformly sprayed onto the pretreated fiberglass membrane (∼17.35 cm^2^). The floatable plate was fully dried to ensure complete immobilization of the catalyst on the fiberglass membrane. Mass transfer within the photocatalytic reactor was enhanced by stirring the liquid at 200 rpm using a magnetic stirrer (IKA, color squid white). The gas output during the reaction was collected in an aluminium-plastic film gas sampling bag (HEDETECH). Finally, the wastewater after reaction was post-treated with PP filter and nanofiltration membrane (NF2012-100, VELLANKELL) in a custom-built integrated system.

## Supplementary Material

nwag440_Supplemental_File
