# Supplementary material for: Offshore solar-driven methane valorization to chloromethane with seawater
Source: Natl Sci Rev. 2026 Jul 16;13(17):nwag440. doi: 10.1093/nsr/nwag440 (PMC13579699; doi:10.1093/nsr/nwag440)
Supplement: Supplementary file 1 [file nwag440_supplemental_file.pdf]

Supplementary Information for

Offshore solar-driven methane valorization to chloromethane with seawater

Zilong Li[1,†], Zili Ma[1,†], Pin Song[2], Junchi Xu[1], Zehua Liu[1,3], Yiming Zhang[1], Yuhe Zhu[1], Chuanjian Wang[1], Hao Yuan[1], Chao Gao[1,*], Federico Rosei[4,*] and Yujie Xiong[1,2,3,*]

[1] Hefei National Research Center for Physical Sciences at the Microscale, School of Chemistry and Materials Science, University of Science and Technology of China, Hefei, Anhui 230026, China.

[2] Anhui Engineering Research Center of Carbon Neutrality, Key Laboratory of Functional Molecular Solids, Ministry of Education, College of Chemistry and Materials Science, Anhui Normal University, Wuhu, Anhui 241000, China.

[3] Suzhou Institute for Advanced Research, University of Science and Technology of China, Suzhou, Jiangsu 215123, China.

[4] Department of Chemical and Pharmaceutical Sciences, University of Trieste, Via Giorgeri 1, 34127, Trieste, Italy.

[†] Zilong Li, Zili Ma contributed equally to this work.

*Correspondence: gaoc@ustc.edu.cn; federico.rosei@units.it; yjxiong@ustc.edu.cn.

METHODS

Reagents and materials

All chemical reagents were used as received without further purification. Anatase TiO_2 (99.8%, 40 nm), rutile TiO_2 (99.8%, 40 nm), ZnO (99.8%, 50 nm), Co_3O_4 (99.5%, 50 nm), $Pt(NH_3)_4Cl_2$ (98%), $Pd(NH_3)_4Cl_2$ (98%), $RuCl_3$ (99.9%), $NiCl_2$ (99.9%), pyrrole (99%), D_2O (99.9%), $Na_2S_2O_3$ (99%) and *t*-BuOH (99.5%) were purchased from Aladdin. All chlorine salts used in the reaction, including LiCl (99%), NaCl (99.8%), KCl (99.8%), CsCl (99.5%), $MgCl_2·6H_2O$ (98%), $CaCl_2$ (96%), $SrCl_2·6H_2O$ (99.5%), $BaCl_2·2H_2O$ (99.5%), $AlCl_3·6H_2O$ (97%), $FeCl_3·6H_2O$ (99%), $CuCl_2·2H_2O$ (99%), $NiCl_2·6H_2O$ (98%) and $MnCl_2$ (98%) were purchased from Sinopharm Chemical Reagent Co., Ltd. 5,5-Dimethyl-1-pyrroline N-oxide (DMPO, 97%) and 2,2,6,6-Tetramethylpiperidine-1-oxyl (TEMPO, 99%) were purchased from TCI Chemicals. Hydrophobic polyvinylidene fluoride (PVDF, 6.6×8.5 cm^2) was purchased from Beyotime Biotechnology Co., Ltd. The fiberglass membrane (47 mm diameter) was purchased from Dongguan Zhongtian Electronics Co., Ltd. The water used in all experiments was ultra-pure water (18.2 MΩ).

Synthesis of photocatalyst

The synthesis process of Pt(x)/TiO_2 photocatalyst can be found in the main text. For comparison, photocatalysts with different oxide supports or metal cocatalysts were synthesized following the identical impregnation–reduction procedure. Only the support material or metal precursor was varied, ensuring consistent preparation conditions across all samples. All catalysts were kept sealed in an inert gas (Ar) to avoid oxidation by air.

Preparation of photothermal PPy–PVDF membrane

Polypyrrole (PPy) was coated on a hydrophobic polyvinylidene fluoride membrane via a modified chemical vapor deposition polymerization (CVDP) process. Firstly, PVDF membrane was immersed in 0.5 M $FeCl_3$ solution for 6 h and dried naturally. The $FeCl_3$-containing membrane was placed in a sealed container with 50 μL pyrrole for *in situ* polymerization. The container was then placed in an oven at 70°C for 3 h. To remove the residual pyrrole, the obtained black membrane was baked in an oven at 120°C for 2 h. Finally, the obtained PPy–PVDF membrane was thoroughly washed with water and dried at 70°C.

Characterization

The crystal structure was identified by X-ray diffraction using the SmartLab (Rigaku) X-ray diffractometer (Cu K_α, 40 kV, 15 mA). Field emission scanning electron microscopy (Zeiss Supra 40), transmission electron microscopy (HITACHI HT7700), high-resolution transmission electron microscopy (JEM F200) and atomic resolution transmission electron microscopy (JEM-ARM200F) were used to image the morphology and structure of the materials. The chemical composition was analyzed by inductively

coupled plasma atomic emission spectroscopy (iCAP 7400) and X-ray photoelectron spectroscopy (AXIS SUPRA+ RATOS, Al K_{α}). The surface area and pore size were measured with the Brunauer-Emmett-Teller method, conducted on the Micromeritics Tristar II 3020. The thermal stability and acid-base sites of the catalysts were evaluated by thermogravimetry (Q5000IR) and programmed adsorption chemical adsorption (AutoChem II 2920). Raman spectra were acquired on a WITec alpha 300R confocal Raman microscope with a 600 grooves/mm diffraction grating. Photoluminescence spectroscopy of the catalysts was collected by steady-state/lifetime spectrofluorometer (Fluorolog-3-Tau and deltaflex), which was used to evaluate the recombination of photogenerated carriers. Diffuse reflection absorption spectra were recorded by UV-Visible-Near infrared spectrophotometer (Shimadzu SolidSpec-3700). The valence-band spectra were measured by ultraviolet photoelectron spectroscopy, and a bias of 5 V was applied when measuring the secondary electron edge. The defect structure of catalysts and the free radical intermediates of the reaction were collected by electron paramagnetic resonance spectrometer (JEOL JES-FA200) equipped with a 500 W xenon lamp (USHIO Optical Modulex SX-U1501XQ) as the illumination source. The Fourier transform infrared spectra (Nicolet 8700) were used to define the structure of PPy–PVDF membranes. The concentration of Cl^- was determined by means of ion chromatography (Dionex ICS-3000).

Photoelectrochemical measurements

Photoelectrochemical measurements were acquired with a three-electrode system on the electrochemical workstation (Corrtest CS300M) in 0.5 M NaCl at room temperature. An H-type photoelectrochemical cell with quartz window was used in the experiment, and the anode and cathode were separated by a proton exchange membrane (Nafion N117). ITO-coated glass substrate (1.0×1.0 cm^2) coated with as-prepared materials were used as working electrodes. Pt plate and saturated calomel electrodes (SCE) functioned as counter and reference electrodes, respectively. To prepare the working electrode, 5 mg of composite catalyst powder was dispersed in 1 mL ethanol and contained with 30 μL of 5 wt% Nafion solution, and the mixture was sonicated for 30 min to form a homogeneous catalyst ink, 200 μL of which was dropped onto the ITO-coated glass substrate at a catalyst loading of 1.0 mg cm^{-2} and dried at room temperature. All photoelectrochemical tests were conducted using a 300 W xenon lamp (Perfect Light PLS-SXE300) equipped with AM1.5G filters as light source and the light intensity was set to 0.5 W cm^{-2}.

***In situ* NAP-XPS measurement**

In situ near ambient pressure XPS (NAP-XPS) measurements were carried out on SpecsLab. To introduce NaCl, 1.0 mg photocatalysts (Pt/TiO_2) were first dispersed in 100 μL NaCl solution (5 mg mL^{-1}) and coated on pre-cleaned Si wafers (1.0×1.0 cm^2). CH_4 gas (99.999 vol.%) was moistened with H_2O before introducing into NAP-XPS chamber (0.45 mbar). A 300 W xenon lamp was used as the illumination source and the light intensity was set to 1.0 W cm^{-2}.

In situ DRIFTS measurement

In situ diffuse reflectance infrared Fourier-transform spectroscopy (DRIFTS) measurements were performed using a Bruker IFS 66v Fourier-transform spectrometer equipped with a Harrick diffuse reflectance accessory at the BL01B beamline in the National Synchrotron Radiation Laboratory (NSRL) in Hefei, China. Each spectrum was collected by taking an average of 128 scans, maintaining a resolution of 4 cm^{-1}. The samples were placed in a custom-fabricated IR reaction chamber, which was specifically designed to examine highly scattering powder samples in the diffuse reflection mode. The chamber was sealed with two ZnSe windows. After sample (mixture of Pt/TiO_2 and NaCl) loading, the chamber was purged with Ar (99.999 vol.%) for 30 min. Then, the samples were measured to obtain background spectra. Subsequently, CH_4 gas (99.999 vol.%) or moist CH_4 gas was introduced into the system. After allowing one hour for surface adsorption to fully stabilize, the spectra under dark were collected. The samples were then exposed to illumination, and the spectra were recorded at various time intervals under gas flow and light illumination. The collected spectra were background-subtracted. The light was introduced using an optical fiber positioned at the front window of the chamber, with a 300 W xenon lamp serving as the light source.

Photocatalytic CH_4 chlorination

The photocatalytic conversion of CH_4 was conducted under atmospheric pressure in a custom-built 350 mL top-irradiation quartz batch reactor. In a typical test, 20 mg catalyst was first suspended and subsequently sonicated in 10 mL deionized water or the aqueous solutions containing chlorides ($AlCl_3$, NaCl, LiCl, KCl, $FeCl_3$, etc.). The reactor was purged with CH_4 gas (99.999 vol.%) or D isotope-labelled CD_4 and sealed carefully. A magnetic stirrer (300 rpm) was used to facilitate the mass transfer between the reactants and photocatalysts. The reactor was irradiated by a 300 W xenon lamp (PLS-SXE300, Perfect Light) with light intensity of 1.6 W cm^{-1}. Such intensity can be easily achieved via a concentrating solar system such as Fresnel lens. After two hours, the reacted gas was syringed from the reactor. A gas chromatographer (GC, 7890B, Ar carrier, Agilent) equipped with a flame ionization detector (FID) and thermal conductivity detector (TCD) was used for the determination of hydrocarbons, CH_3Cl, and H_2 in the reacted gas. Another GC (7890A, Ar carrier, Agilent) equipped with a methanizer, FID detector, and TCD detector was used to analyze CO and CO_2 products. The isotope-labelled experiments were performed by using CD_4 as feeding gas, and the products were analyzed by gas chromatography-mass spectrometry (GC-MS, 7890 A and 5975 C, He carrier, Agilent). Liquid products were analyzed by the 1H nuclear magnetic resonance (NMR) spectroscopy (400 MHz, AVANCE 400). Seawater was sourced from the East China sea in June 2024.

Supplementary Figures

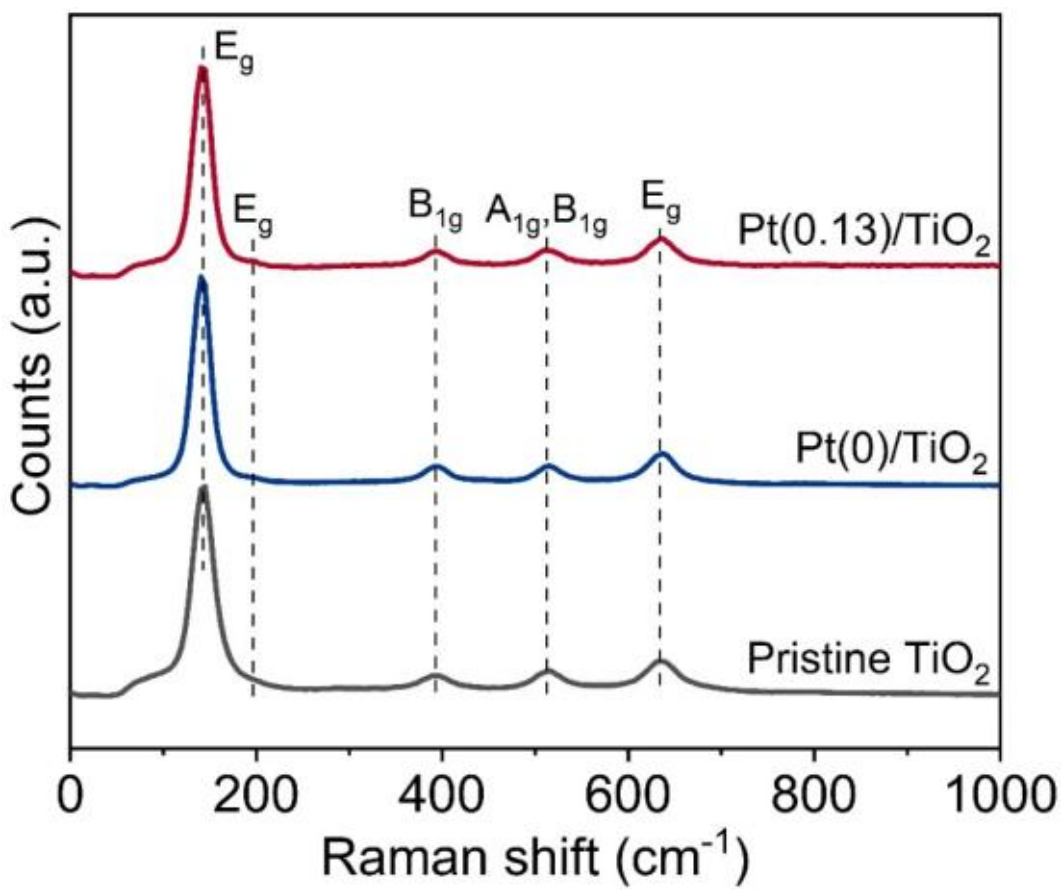

Fig. S1 | Raman spectra of pristine TiO_2, Pt(0)/TiO_2 and Pt(0.13)/TiO_2.

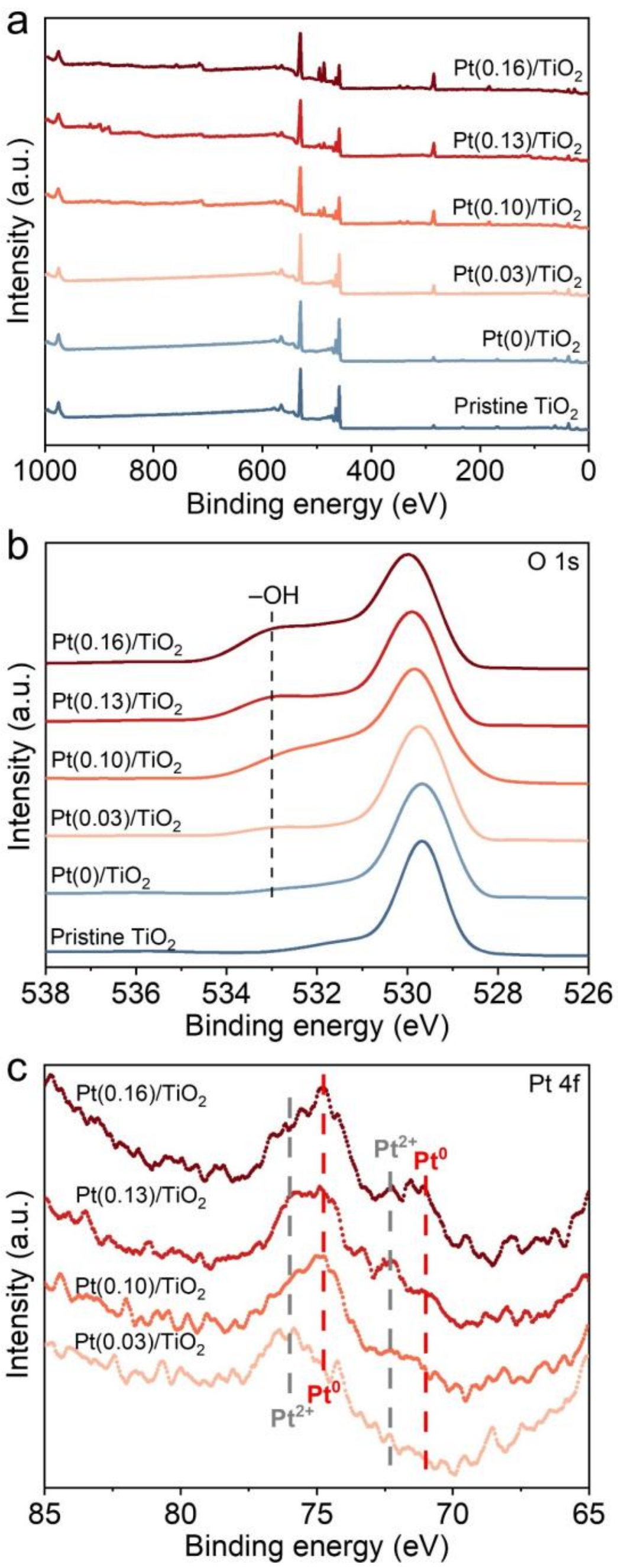

Fig. S2 | (**a**) X-ray photoelectron spectroscopy (XPS) survey, and high-resolution XPS spectra of (**b**) O 1s and (**c**) Pt 4f for pristine TiO_2 and Pt(x)/TiO_2.

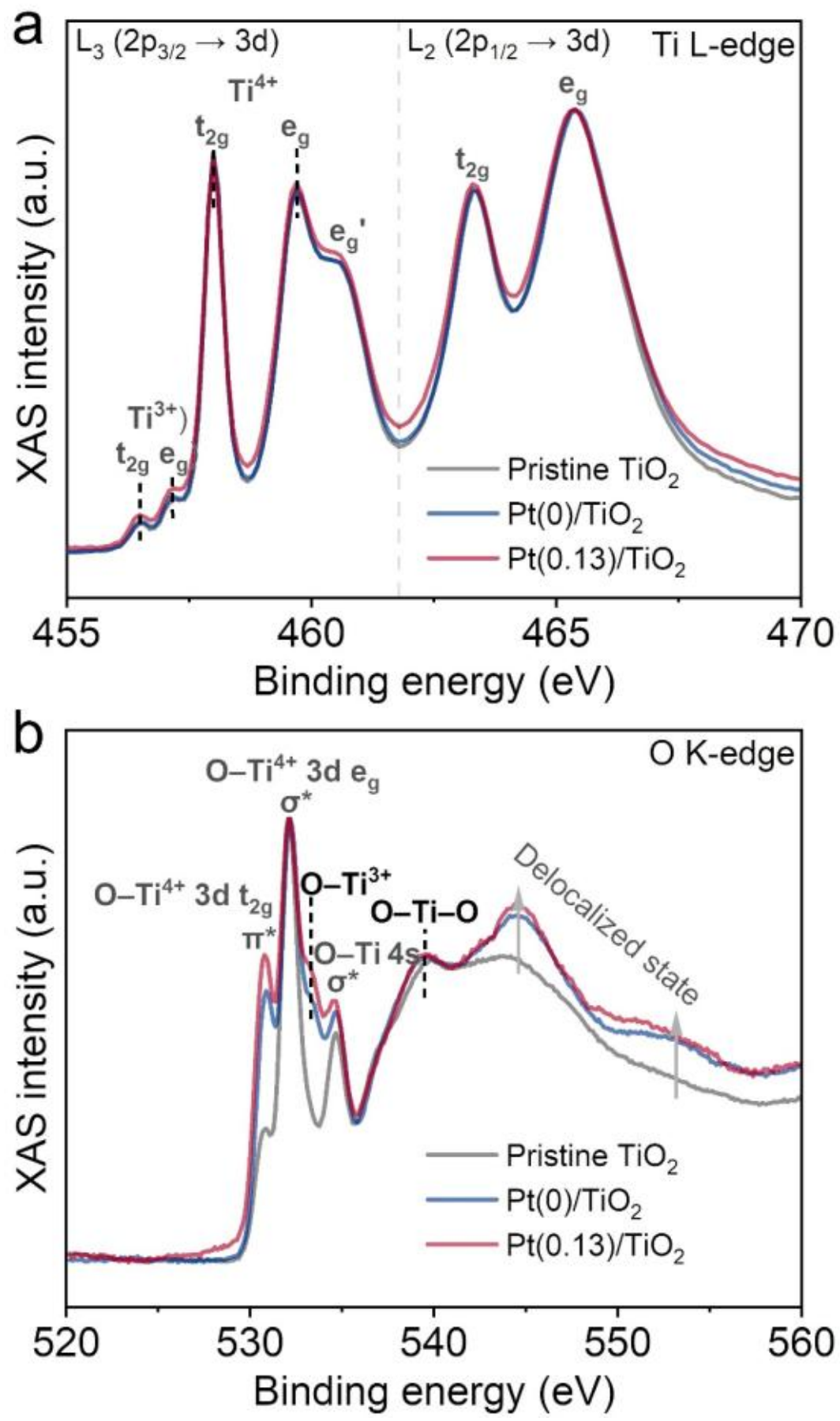

Fig. S3 | (**a**) Ti L-edge and (**b**) O K-edge X-ray absorption spectra (XAS) for pristine TiO_2, Pt(0)/TiO_2 and Pt(0.13)/TiO_2.

Upon Pt loading and H_2 treatment, the Pt(0.13)/TiO_2 sample exhibits significantly enhanced XAS spectral features assigned to Ti^{3+} species, especially in the Ti L_3-edge region where prominent shoulders (~456.5 and 457.1 eV) emerge near the t_{2g} and e_g transitions.[1] Concurrently, the O K-edge spectrum reveals an enhanced shoulder (~533.3 eV), corresponding to O 2p-Ti^{3+} hybridized states. The post-edge region of both edges shows intensified delocalized state contributions, indicating increased electronic conductivity and defect-induced orbital overlap. These spectral changes confirm the formation of Ti^{3+} and oxygen vacancy states upon Pt loading and reduction, supporting the establishment of a pronounced Schottky junction at the Pt–TiO_2 interface. Such defect-related states are expected to promote charge separation and facilitate photocatalytic performance.

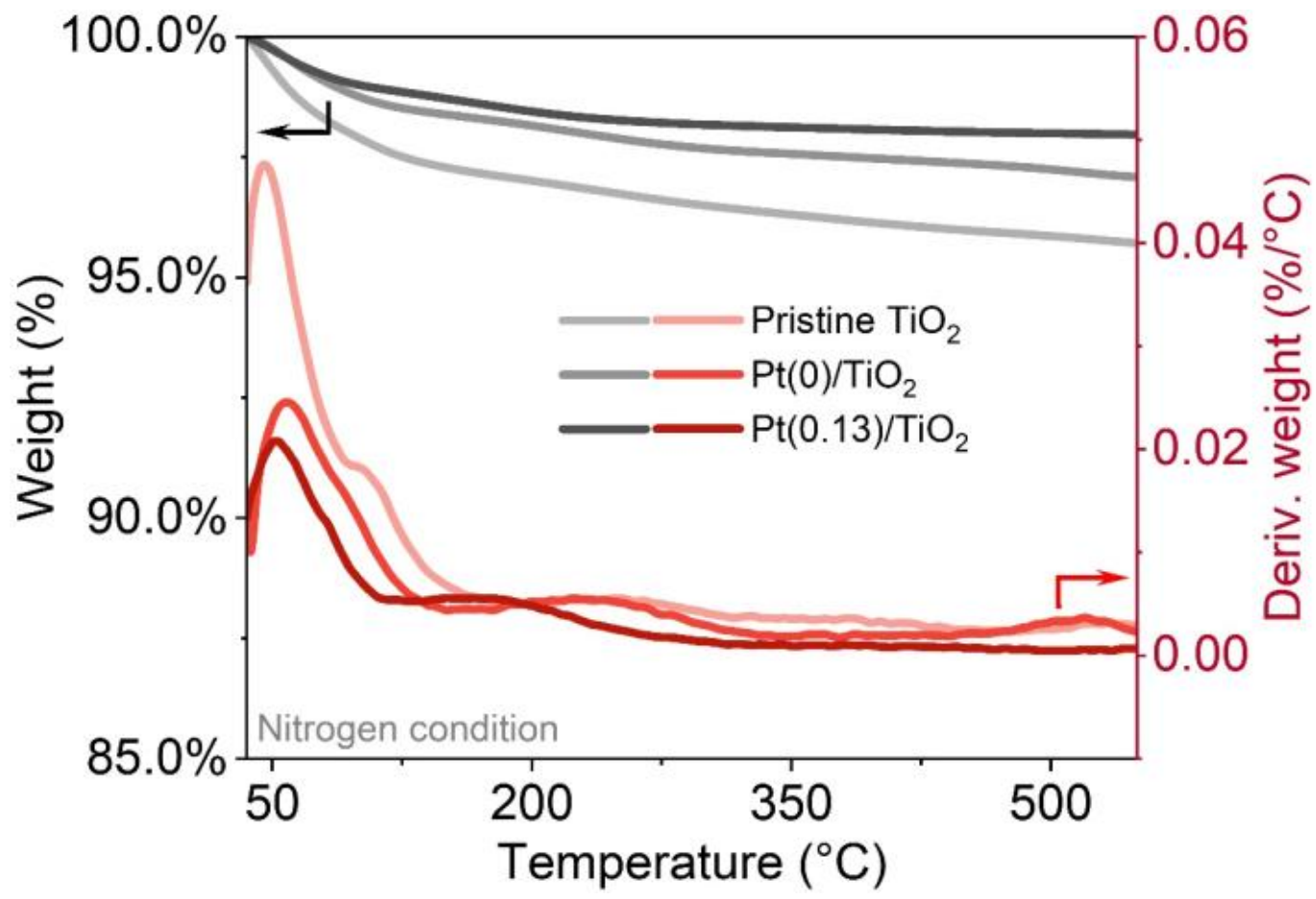

Fig. S4 | Thermogravimetric analysis (TGA) and derivative thermogravimetric (DTG) curves of pristine TiO_2, Pt(0)/TiO_2 and Pt(0.13)/TiO_2.

TGA reveals that all samples, including TiO_2 and Pt-loaded TiO_2, exhibit excellent thermal stability with total weight losses below 5% up to 600 °C. The DTG curves show a clear peak below 100 °C, indicating that the observed mass loss in this region is primarily due to the desorption of physically adsorbed water. Beyond this range, only a slight and gradual weight loss is observed, with no significant peaks in the DTG curves, suggesting minimal structural degradation and the slow release of a small amount of surface-adsorbed species or lattice oxygen (a typical occurrence for stable metal oxides).[2] Among them, Pt(0.13)/TiO_2 shows the lowest overall mass loss, confirming its superior thermal stability. These results support the conclusion that Pt(0.13)/TiO_2 is a thermally robust photocatalyst, capable of withstanding high-intensity illumination conditions that may induce local heating.

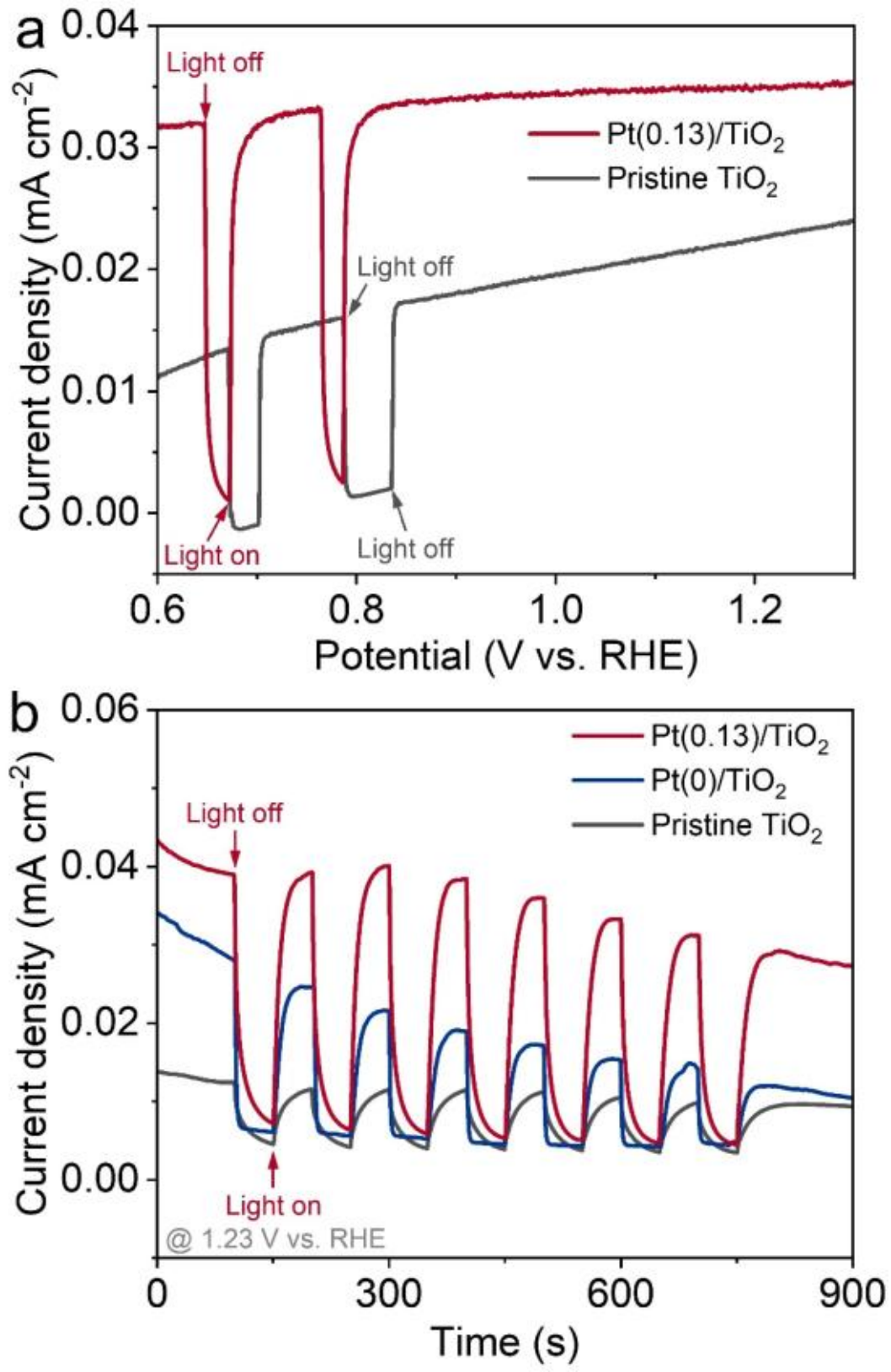

Fig. S5 | (**a**) Intermittent light-on/off linear sweep voltammetry (LSV) curves of pristine TiO_2 and Pt(0.13)/TiO_2. (**b**) Photocurrent measurements at 1.23 V vs. RHE for pristine TiO_2, Pt(0)/TiO_2 and Pt(0.13)/TiO_2.

Both intermittent light-on/off LSV and photocurrent measurements revealed an enhanced photoresponse, highlighting the critical roles of Pt species and oxygen vacancies in facilitating charge formation, transport and separation.

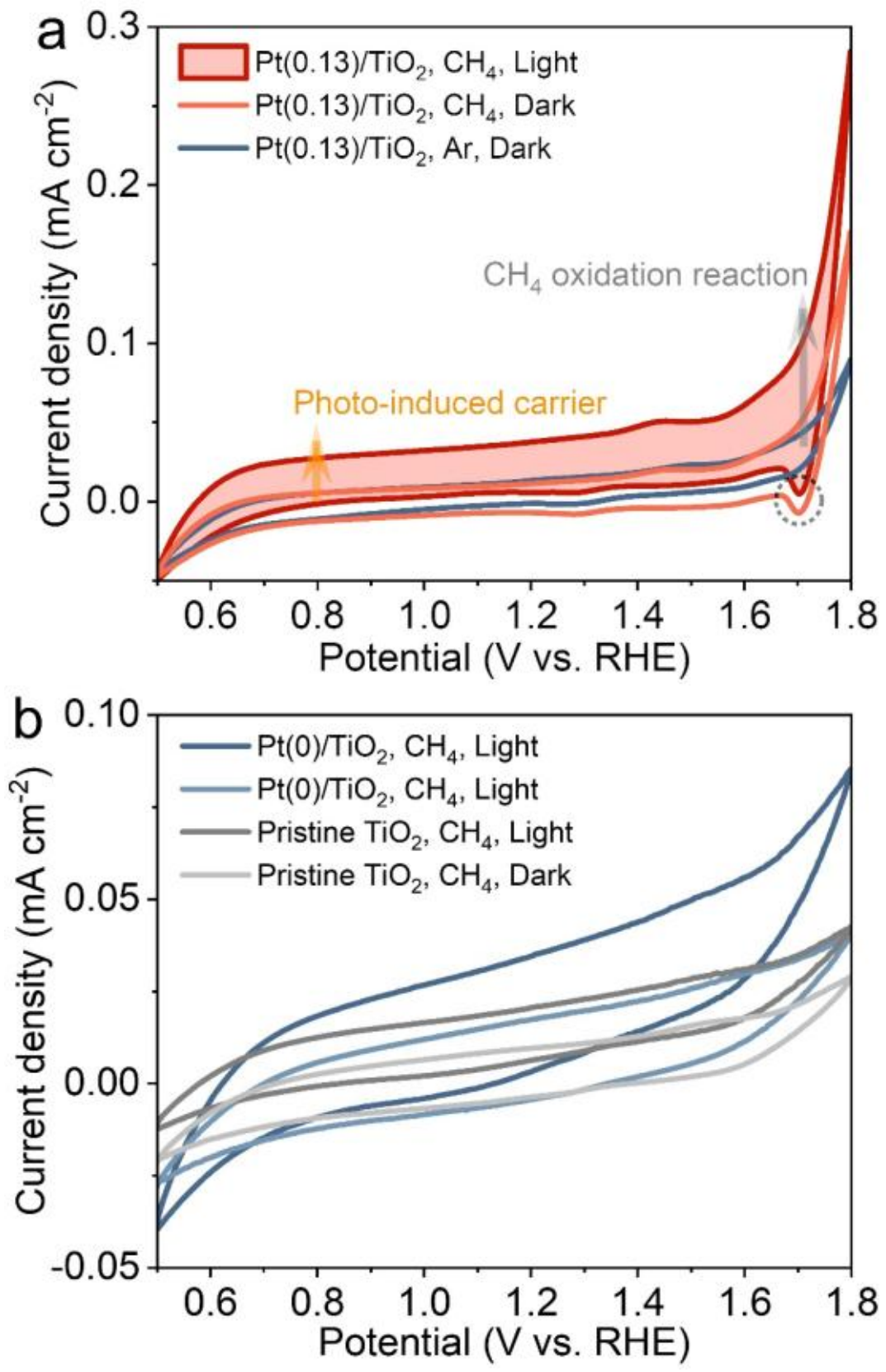

Fig. S6 | Cyclic voltammetry curves of (**a**) Pt(0.13)/TiO_2, and (**b**) pristine TiO_2 and Pt(0)/TiO_2 under different atmospheres (Ar or CH_4) and different light conditions (dark or AM 1.5G).

In the cyclic voltammetry curves, the overall current density of Pt(0.13)/TiO_2 increased markedly following the introduction of CH_4 and light. Compared to TiO_2 without Pt modification, characteristic reduction peaks of Pt species were observed exclusively in the negative scanning segments of the Pt(0.13)/TiO_2 photocatalyst, providing direct evidence of CH_4 activation by the Pt cocatalyst.[3]

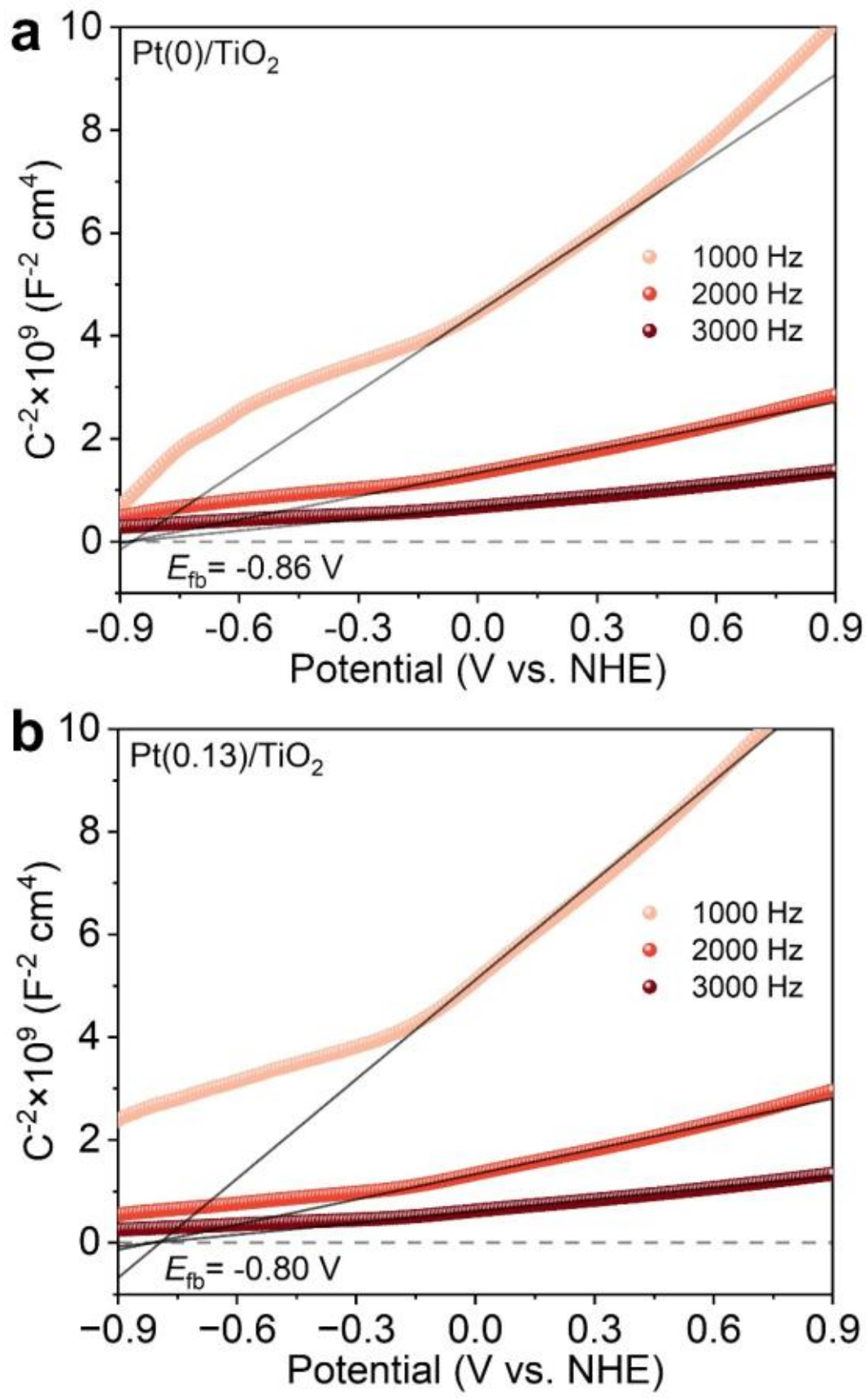

Fig. S7 | Mott-Schottky curves of (**a**) Pt(0.13)/TiO_2 and (**b**) Pt(0)/TiO_2 at frequencies of 1000, 2000 and 3000 Hz. E_{fb}, flat band potential, represents the balanced distribution of electron inside the semiconductor.

The higher flat band potential of Pt(0.13)/TiO_2 (-0.80 V vs. NHE), as determined from Mott-Schottky analysis, indicates a more positive conduction band edge when compared to Pt(0)/TiO_2 (-0.86 V vs. NHE). This upshift enhances the oxidative power of photogenerated holes, which is favorable for hole-driven photocatalytic methane chlorination. The more positive potential also suggests improved charge separation efficiency, thereby contributing to the superior photocatalytic activity.[4]

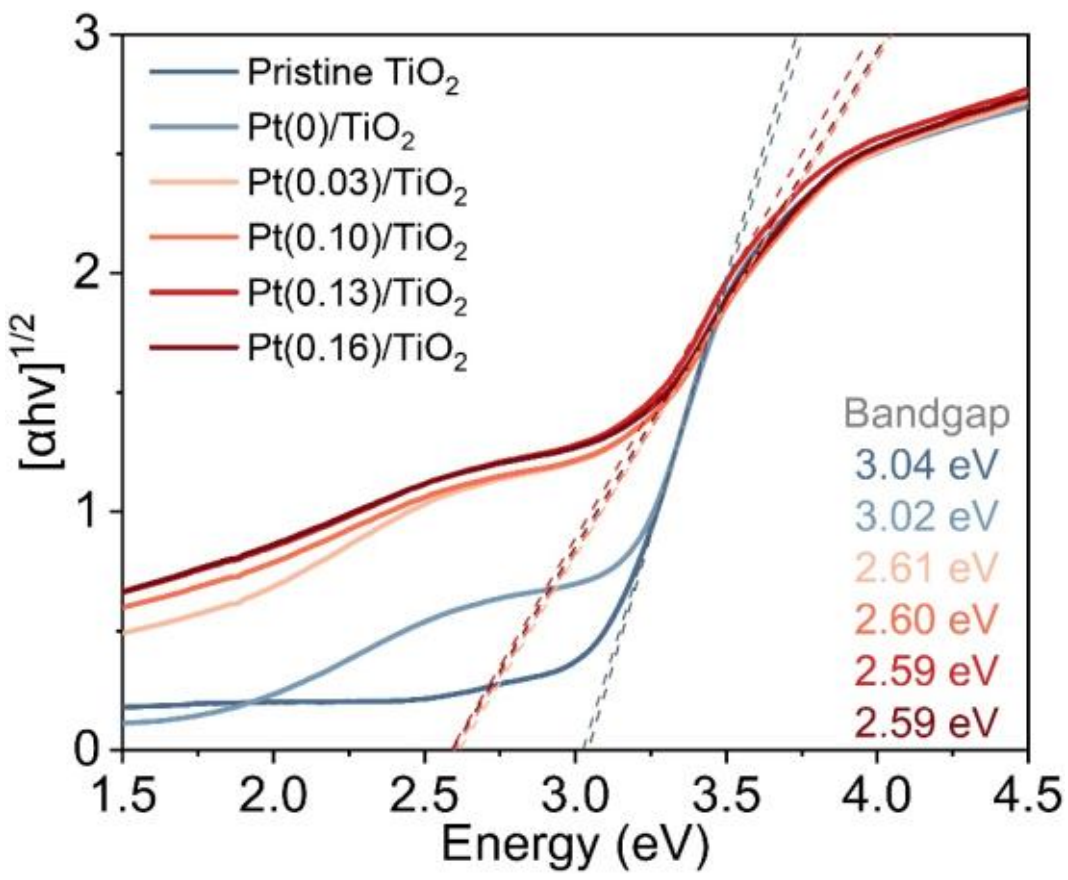

Fig. S8 | Tauc plots for pristine TiO_2 and Pt(x)/TiO_2 photocatalysts. The dashed lines represent the extrapolation for the determination of the indirect band gap.

The band gap of the prepared photocatalysts was determined using the Kubelka–Munk (K–M) function in conjunction with Tauc plots.[5] The band gap (E_g) for pristine TiO_2 and Pt(0)/TiO_2 was determined to be 3.04 and 3.02 eV, respectively, consistent with the classical TiO_2 range of 3.0–3.2 eV. The E_g of the Pt(0.13)/TiO_2 nanocomposite was determined to be 2.59 eV, indicating the influence of Pt species and the Schottky barrier on modulating band structure. The presence of Pt introduces localized states or enhances interfacial charge transfer, which can effectively narrow the apparent optical band gap by facilitating sub-bandgap absorption.

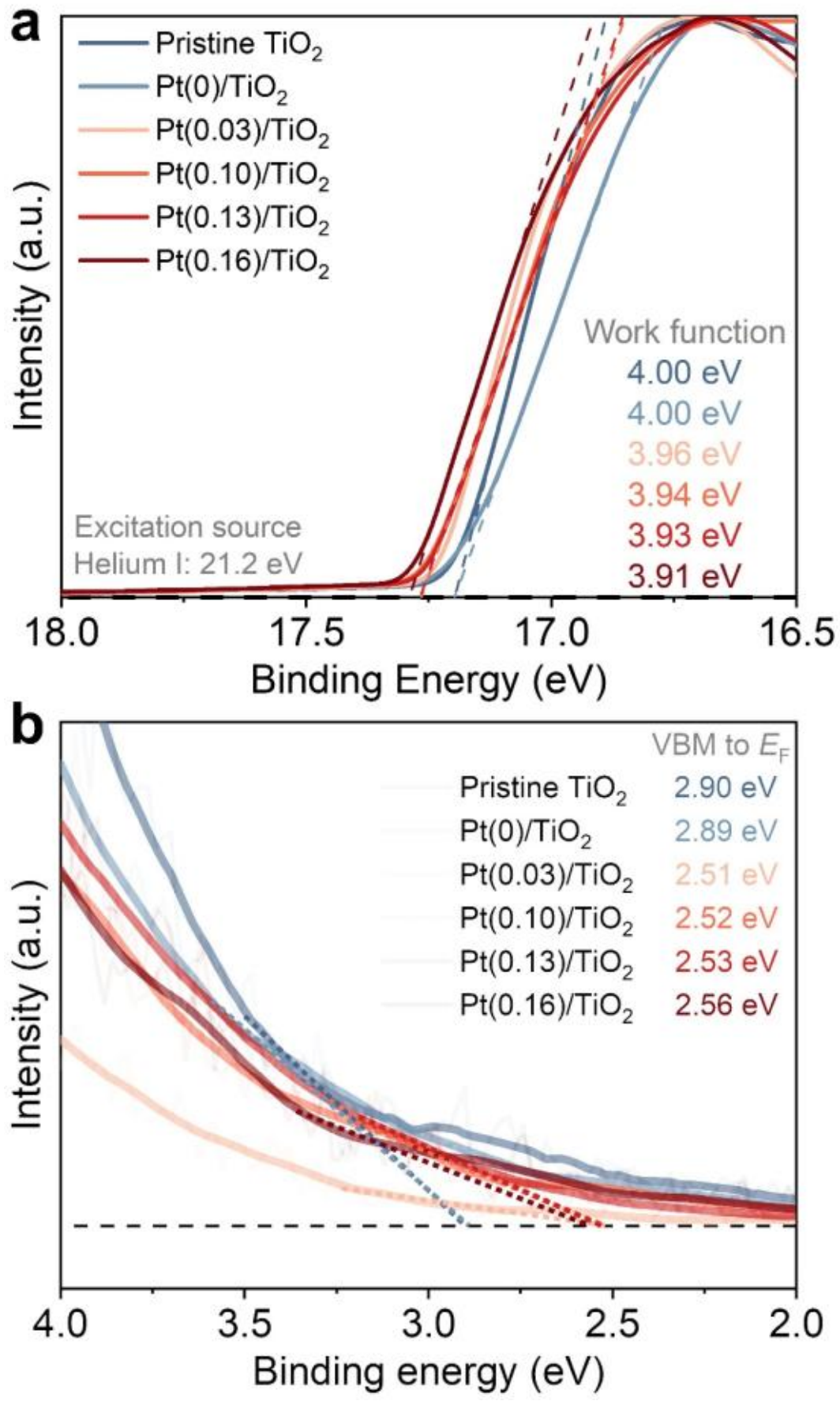

Fig. S9 | (a) The secondary electron cutoffs and calculated work functions for pristine TiO_2 and Pt(x)/TiO_2. (**b**) The Fermi edge and estimated gaps from the valence band maximum (VBM) to the Fermi level for pristine TiO_2 and Pt(x)/TiO_2.

Ultraviolet photoelectron spectroscopy (UPS) was employed to determine the secondary electron cutoffs (E_{cutoff}) and Fermi edges for resolving the energy band structures. The work functions (W_f) of pristine TiO_2 and Pt(x)/TiO_2 were comparable, ranging from 4.00 to 3.91 eV. The slight reduction of W_f induced by Pt modification further confirmed the interaction between Pt cocatalyst and TiO_2 support, indicating electron transfer from Pt to TiO_2. Fermi edge analysis revealed gaps from the valence band maximum (VBM) to the Fermi level of 2.90 eV for pristine TiO_2 and 2.53 eV for Pt(0.13)/TiO_2, confirming that Pt decoration significantly shifted the valance band position and altered the energy band structure. Detail information of energy band structures for pristine TiO_2 and Pt(x)/TiO_2 were provided in Table S3.

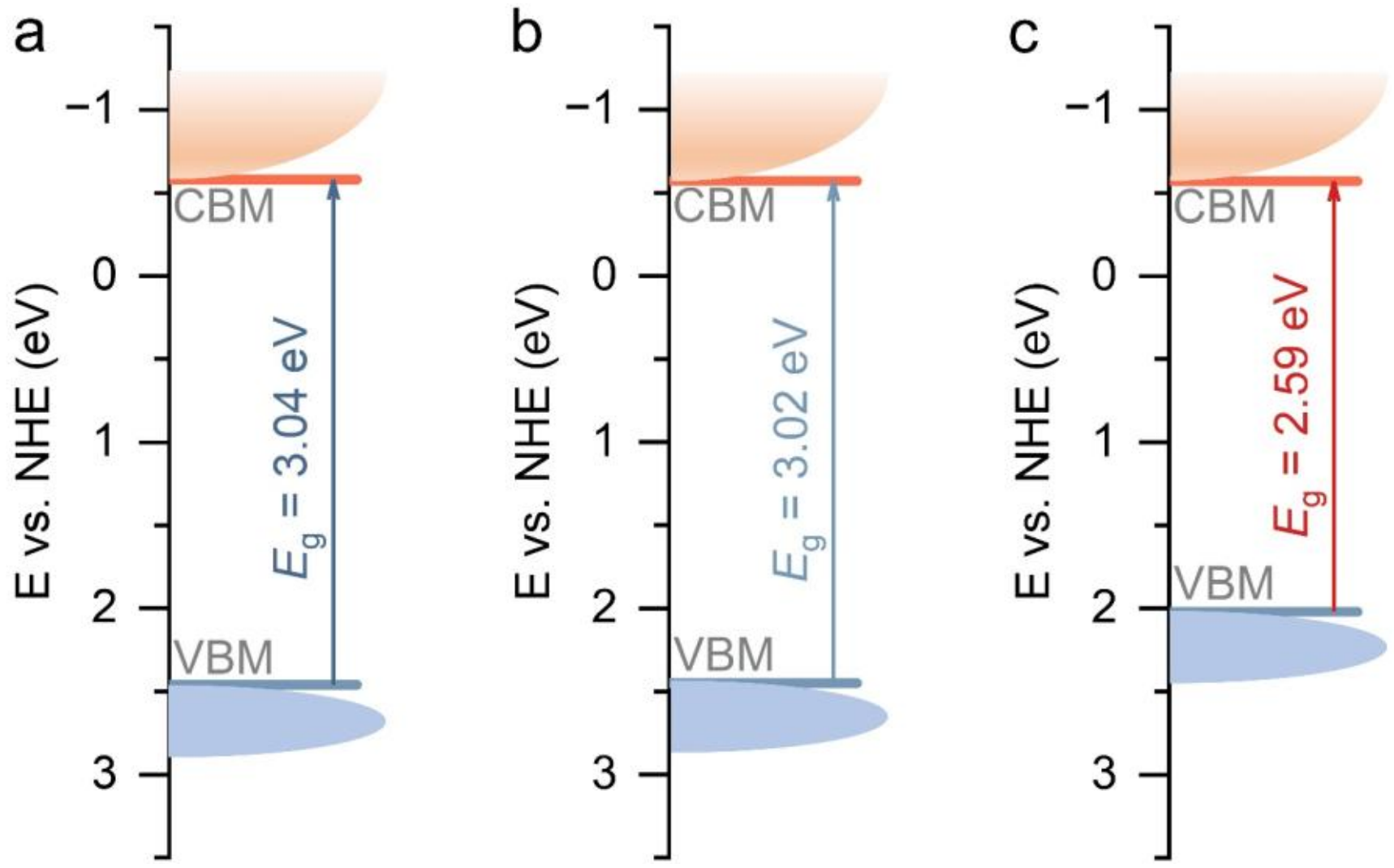

Fig. S10 | Schematic illustration for the energy band structures of pristine TiO_2, Pt(0)/TiO_2 and Pt(0.13)/TiO_2. CBM, conduction band maximum; VBM, valence band maximum; E_g, energy of band gap; NHE, normal hydrogen electrode, is equivalent to E_V - 4.44 eV.

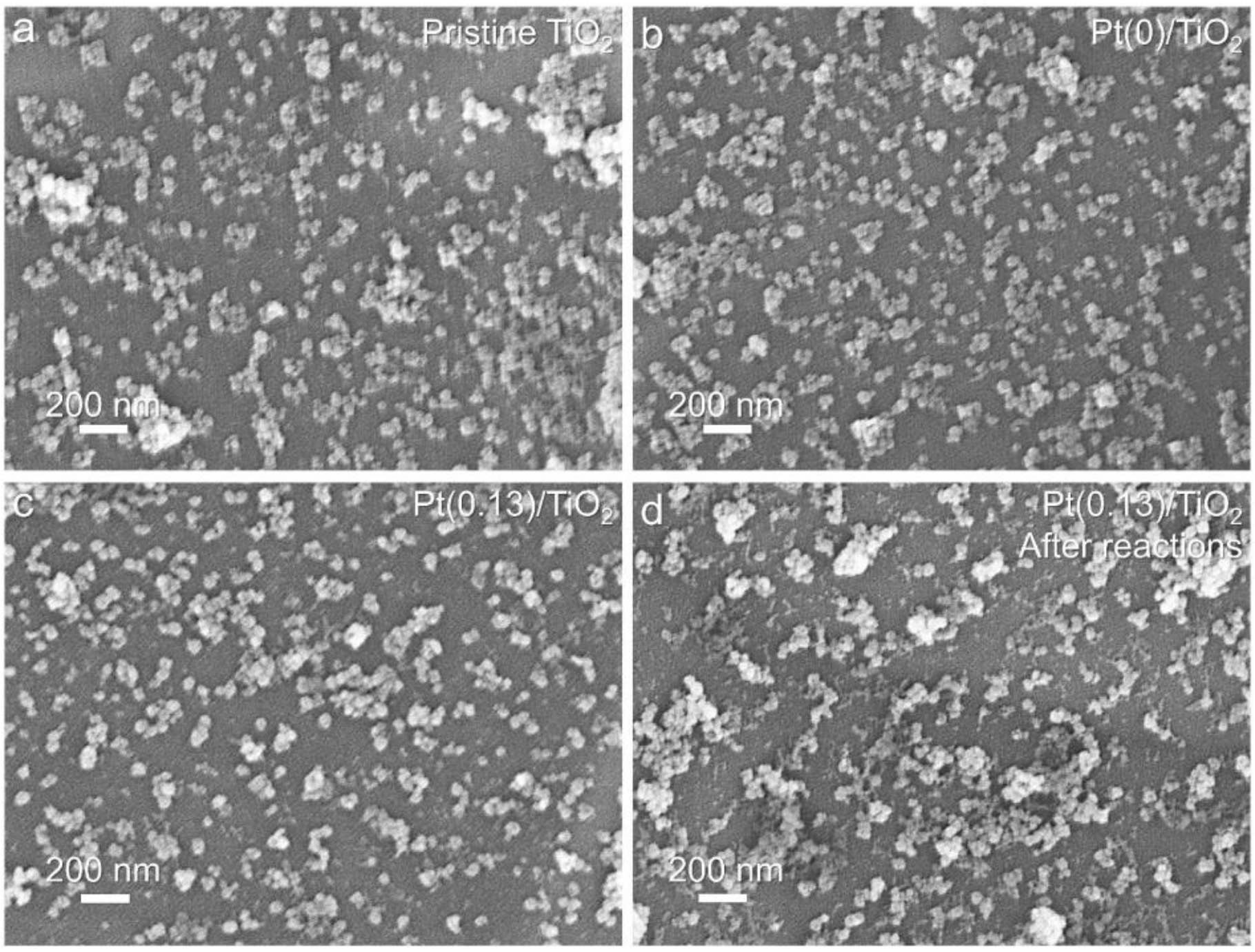

Fig. S11 | Field emission scanning electron microscopy (FESEM) images of (**a**) pristine TiO_2, (**b**) Pt(0)/TiO_2, and (**c, d**) Pt(0.13)/TiO_2 before and after reactions.

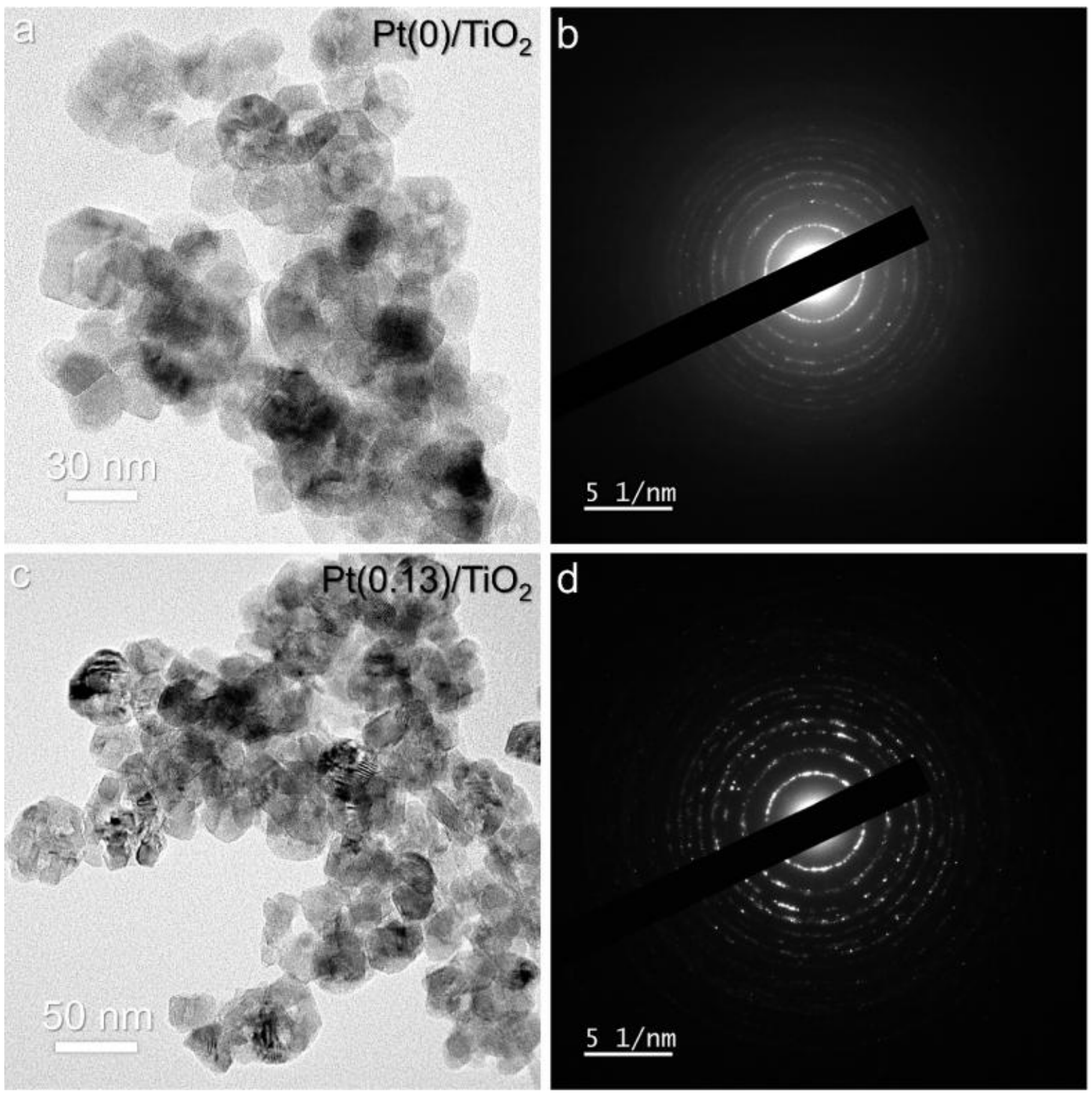

Fig. S12 | Transmission electron microscopy (TEM) images and selected area electron diffraction (SAED) patterns of (**a, b**) Pt(0)/TiO_2, and (**c, d**) Pt(0.13)/TiO_2.

Selected area electron diffraction patterns (Fig. S12 b,d) revealed additional diffraction spots in Pt(0.13)/TiO_2, confirming the formation of Pt nanoparticles (NPs).

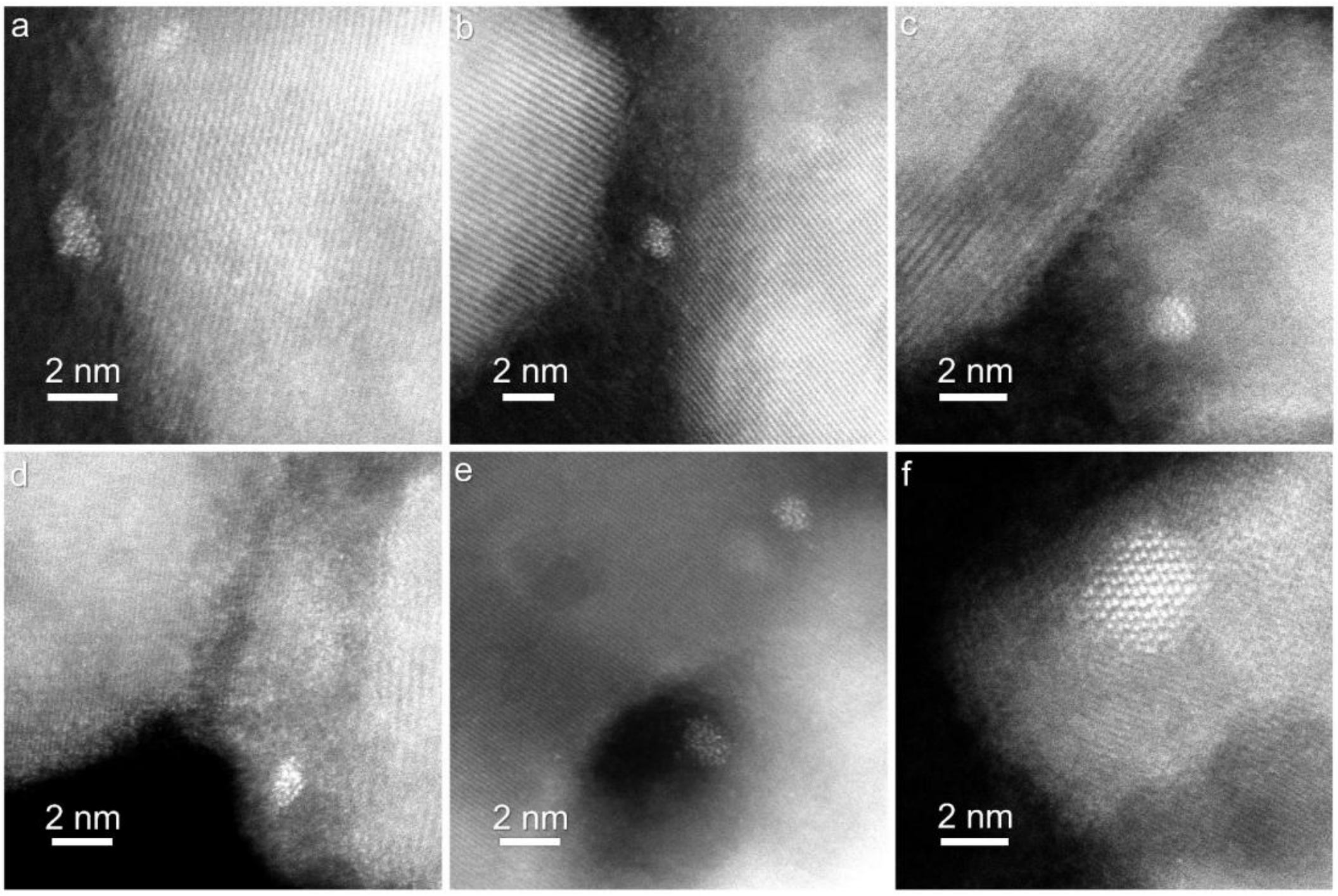

Fig. S13 | Aberration-corrected transmission electron microscopy (ACTEM) images of Pt(0.13)/TiO_2.

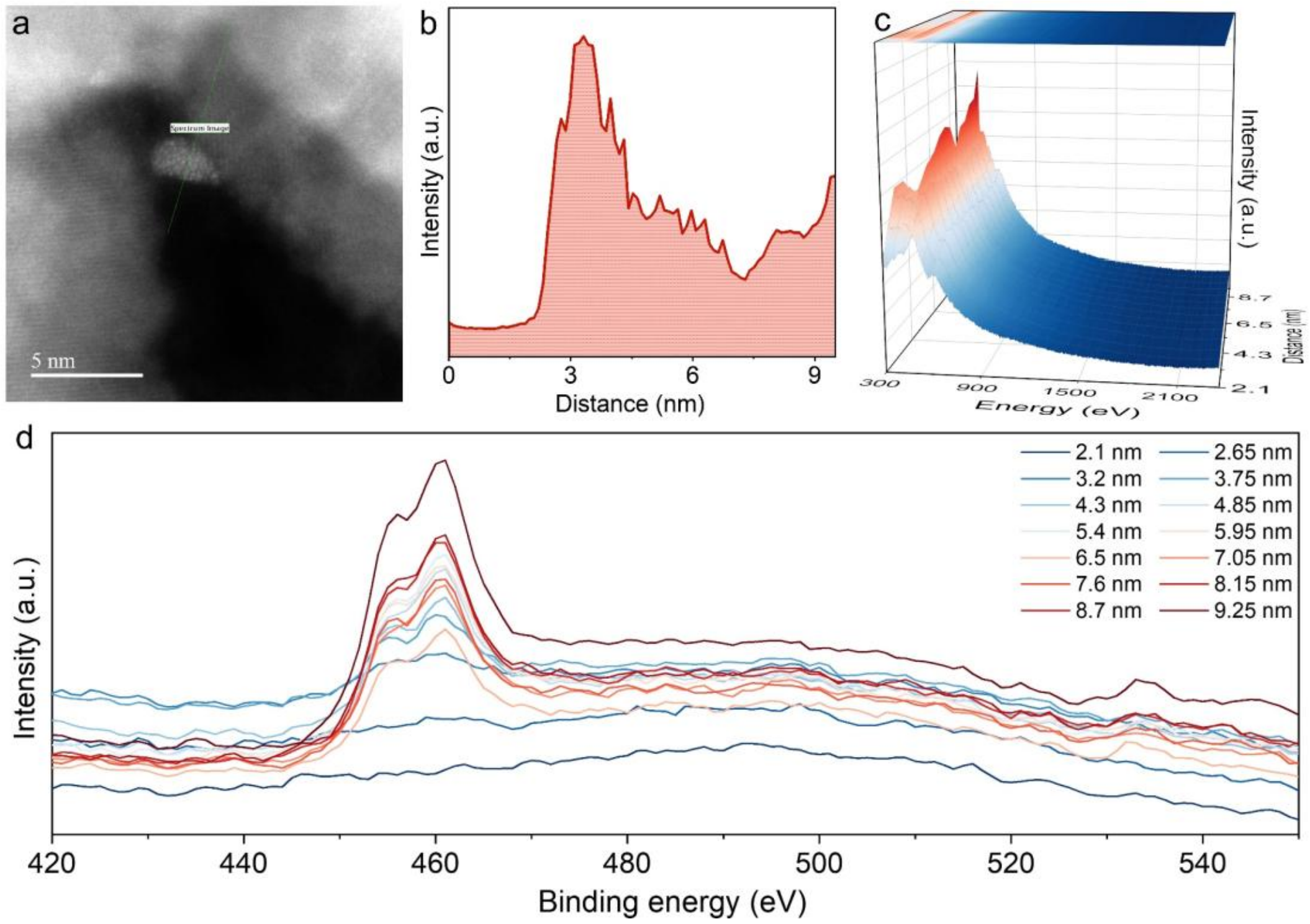

Fig. S14 | Line-scanning electron energy loss spectroscopy (EELS) of Pt(0.13)/TiO_2 measured using ACTEM. (**a**) Survey image, (**b**) dark field scan image, (**c**) EELS spectra in matrix form, and (**d**) EELS spectra of Ti L-edge analyzed at different distances.

Fig. S14 shows EELS line-scan spectra acquired across a Pt nanoparticle on TiO_2. The Ti $L_{2,3}$-edge (Fig. 14d) displays a slight positive energy shift and increased L_3–L_2 splitting within ~3–5 nm of the Pt–TiO_2 contact, while the spectra recorded beyond ~6 nm revert to the bulk anatase signature. Such spectral evolution is attributed to a local modification of ligand-field symmetry and charge polarization at interfacial Ti sites rather than a uniform reduction from Ti^{4+} to Ti^{3+}. In other words, the data indicate a localized electronic/coordination perturbation induced by the metal–oxide interaction. This interfacial restructuring is expected to increase the Lewis-acidic character of surface Ti centers and to alter adsorption energetics for anions such as Cl^-. Taken together with complementary UPS and Mott–Schottky results, the EELS data support a picture in which Pt creates a spatially confined electronic microenvironment that modulates acid-base properties at the surface. This modulation provides a basis for enhanced chloride adsorption and stabilization of surface-bound reaction intermediates implicated in the observed radical-relay chlorination.

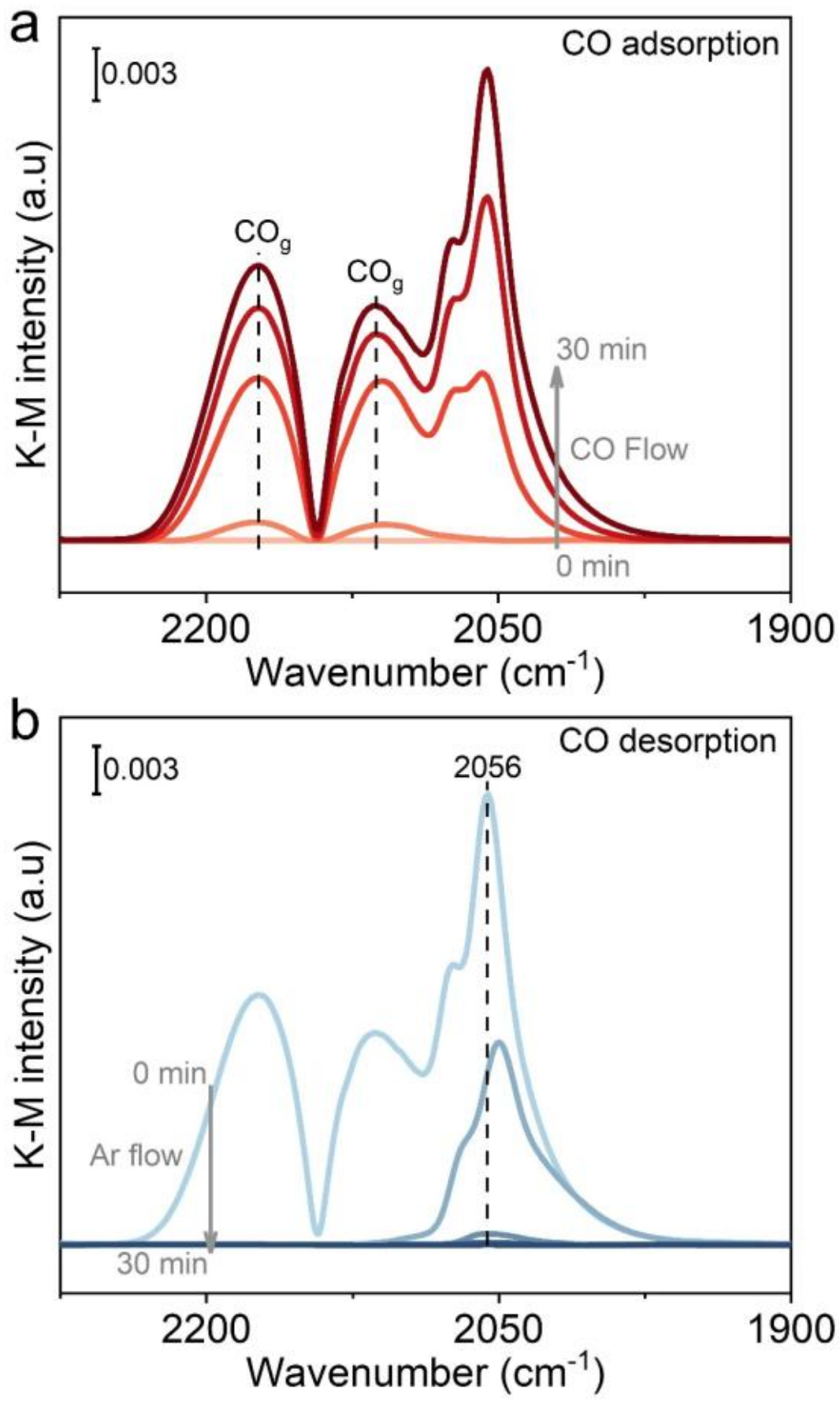

Fig. S15 | In situ diffuse reflectance infrared Fourier transform spectra (DRIFTS) with carbonic oxide as probe molecule for Pt(0.13)/TiO_2 at the temperature of 473 K. The time of collecting spectra was 0, 5, 10, 15 and 30 min, respectively.

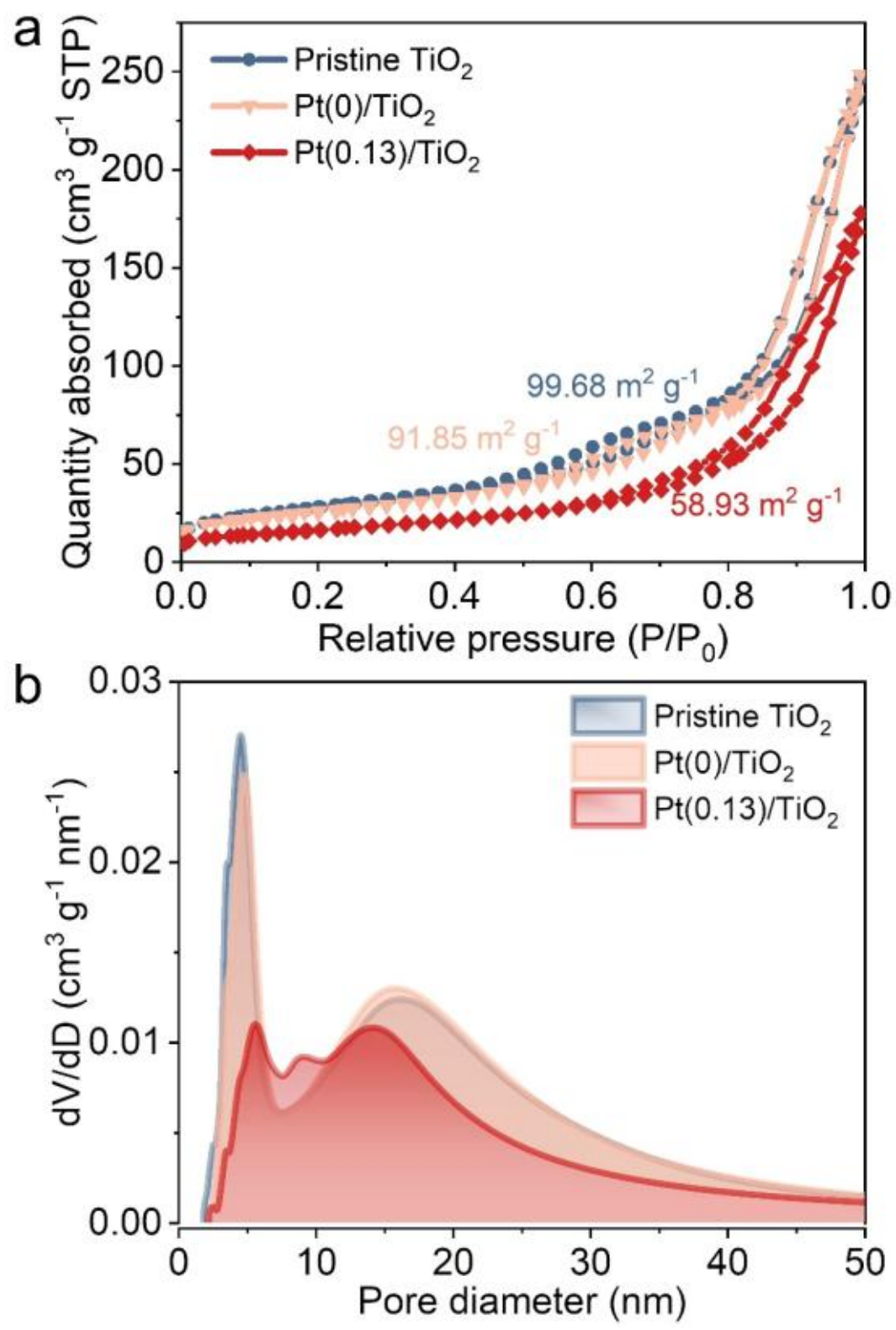

Fig. S16 | (a) N_2 adsorption-desorption isotherm and (**b**) the corresponding mesopore size distribution of pristine TiO_2, Pt(0)/TiO_2 and Pt(0.13)/TiO_2.

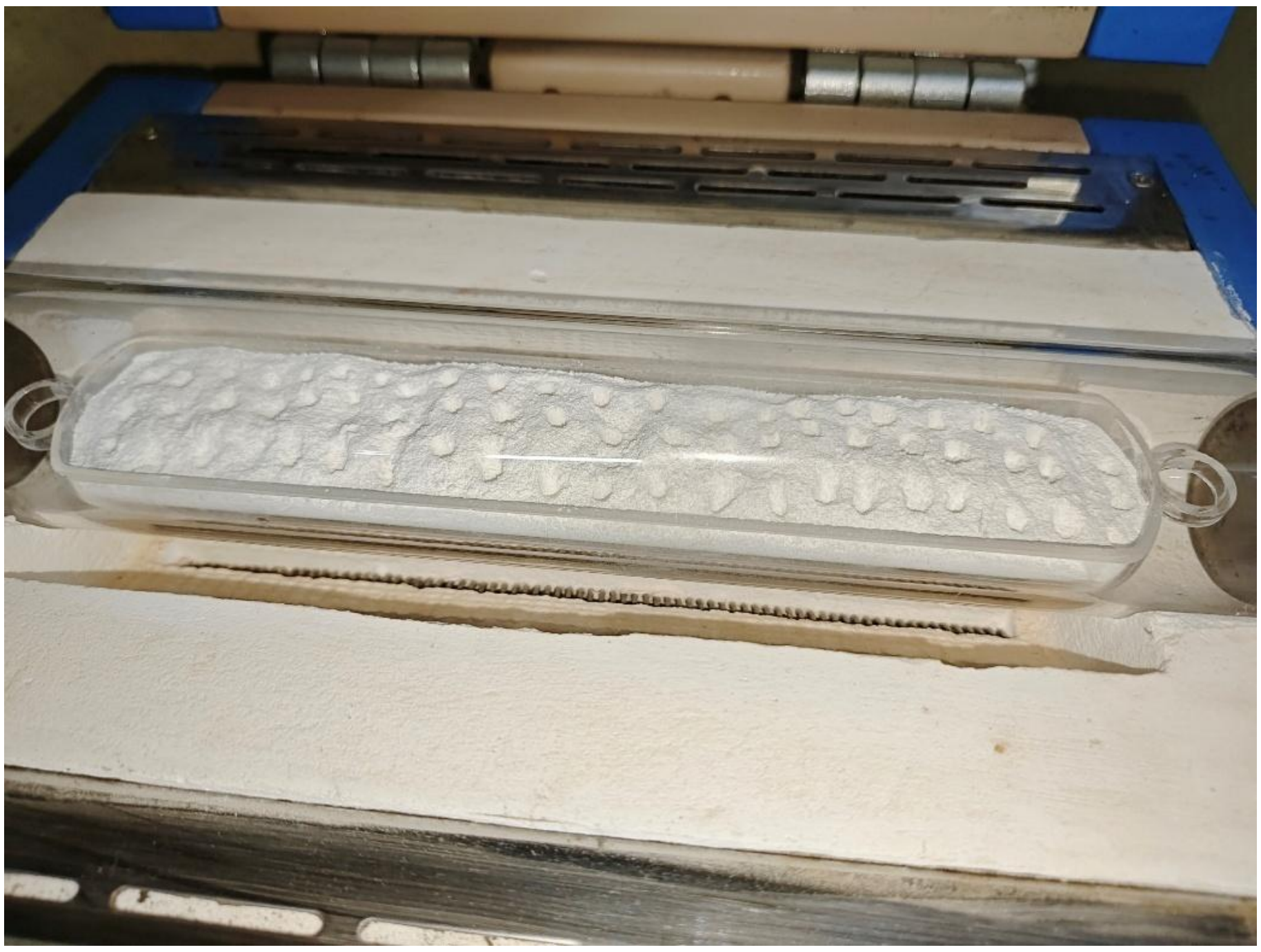

Fig. S17 | Photograph of the heat treatment step in the scaled-up procedure for Pt(0.13)/TiO_2 photocatalyst, which was taken before it was annealed.

Cost estimation for the scaled-up synthesis of Pt(0.13)/TiO_2 photocatalyst.

To evaluate the economic feasibility of the scaled-up synthesis of the Pt(0.13)/TiO_2 photocatalyst, a cost estimation was conducted based on the actual material inputs and operational parameters. In the scaled-up procedure, 0.250 g of $Pt(NH_3)_4Cl_2$ (purchased from Aladdin, ~\$12) and 110 g of TiO_2 powders (purchased from Aladdin, ~\$5) were used to yield 106.0 g of the final catalyst. The procedure included impregnation, drying at 80 °C, and high-temperature reduction under 10% H_2/Ar at 773 K for 5 hours. The total electricity consumption for the drying and reduction steps was estimated to be approximately 5 kWh (1 kWh for drying and 4 kWh for annealing), corresponding to an energy cost of \$0.40 based on a rate of \$0.08 per kWh. Additional expenses for consumables such as distilled water, reducing gas (H_2/Ar) and inert atmosphere (Ar) were collectively estimated at \$1.60. Therefore, the total production cost amounts to approximately \$19.00, corresponding to an estimated unit cost of \$0.18 per gram of Pt(0.13)/TiO_2 catalyst. These results highlight the promising economic viability of the Pt(0.13)/TiO_2 photocatalyst for practical applications.

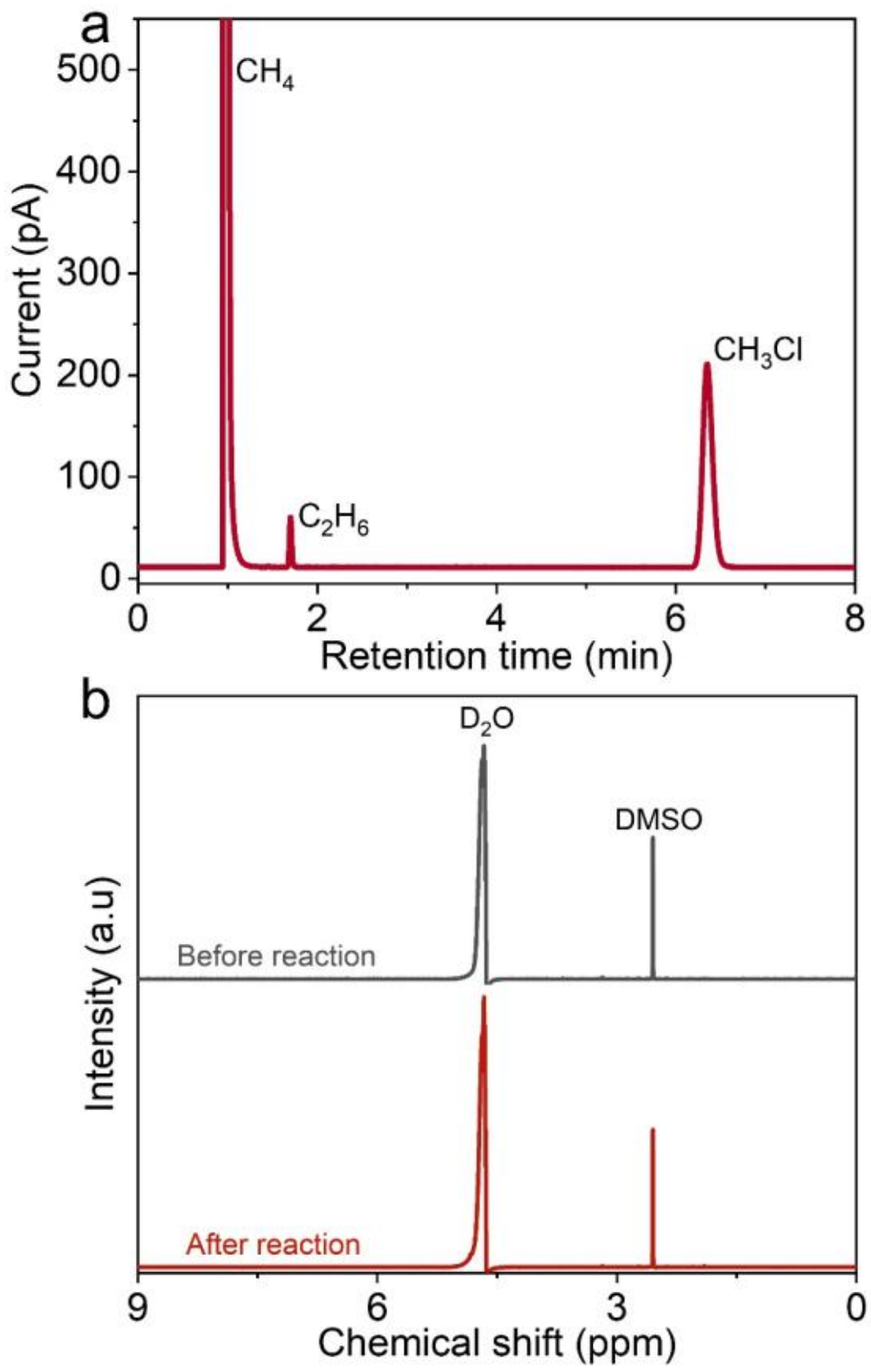

Fig. S18 | (a) Typical gas chromatography (GC) traces of photocatalytic CH_4 conversion using Pt(0.13)/TiO_2 photocatalyst. (**b**) ^{1}H NMR spectra of the products generated using Pt(0.13)/TiO_2 photocatalyst before and after reaction.

Gas chromatography and ^{1}H nuclear magnetic resonance confirmed that CH_3Cl was the dominant product, with only a small amount of the C_2H_6 by-product, indicating that CH_4 overoxidation was suppressed.

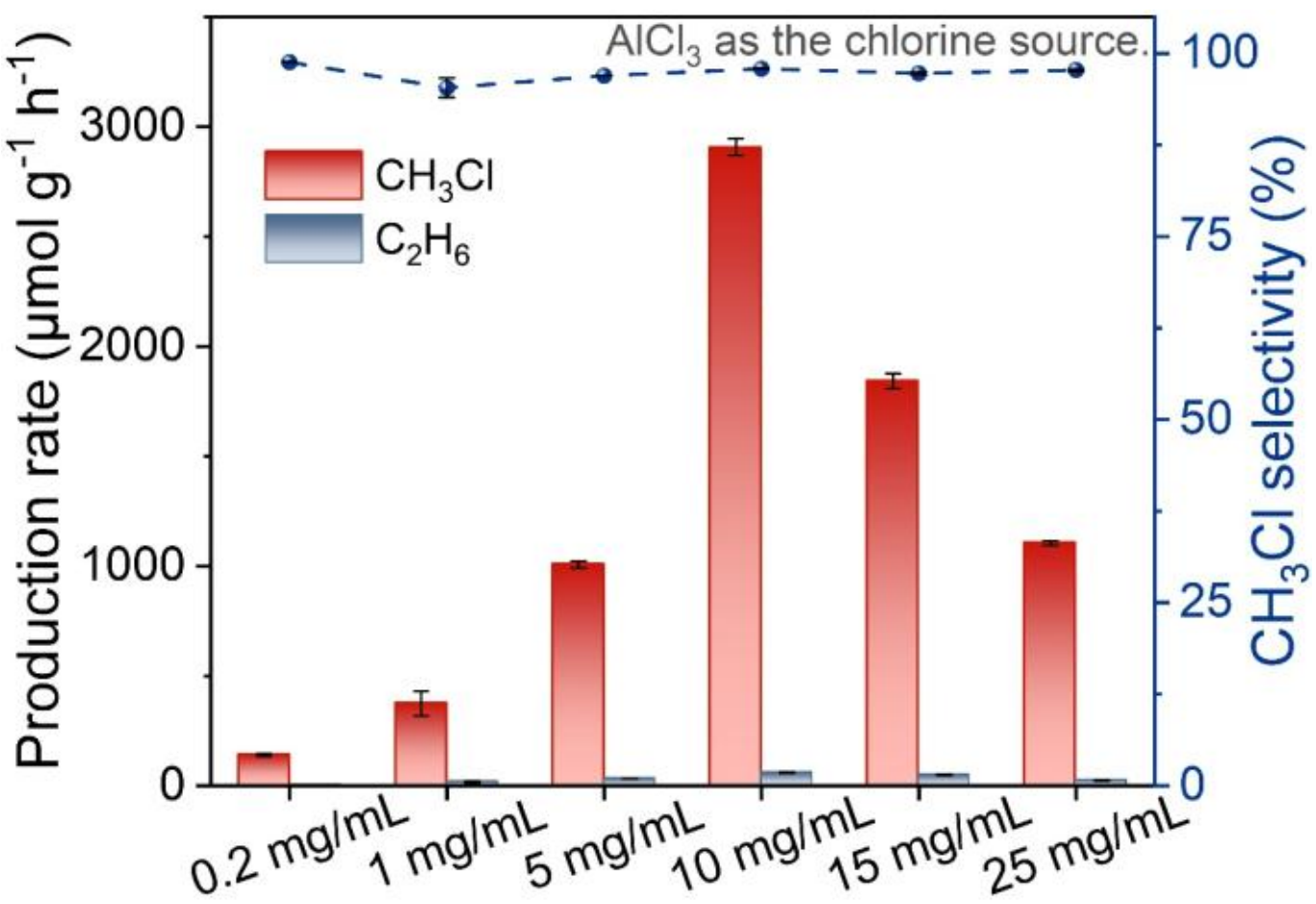

Fig. S19 | Product yields and CH_3Cl selectivity over Pt(0.13)/TiO_2 photocatalyst in different concentrations of $AlCl_3 \cdot 6H_2O$ solution.

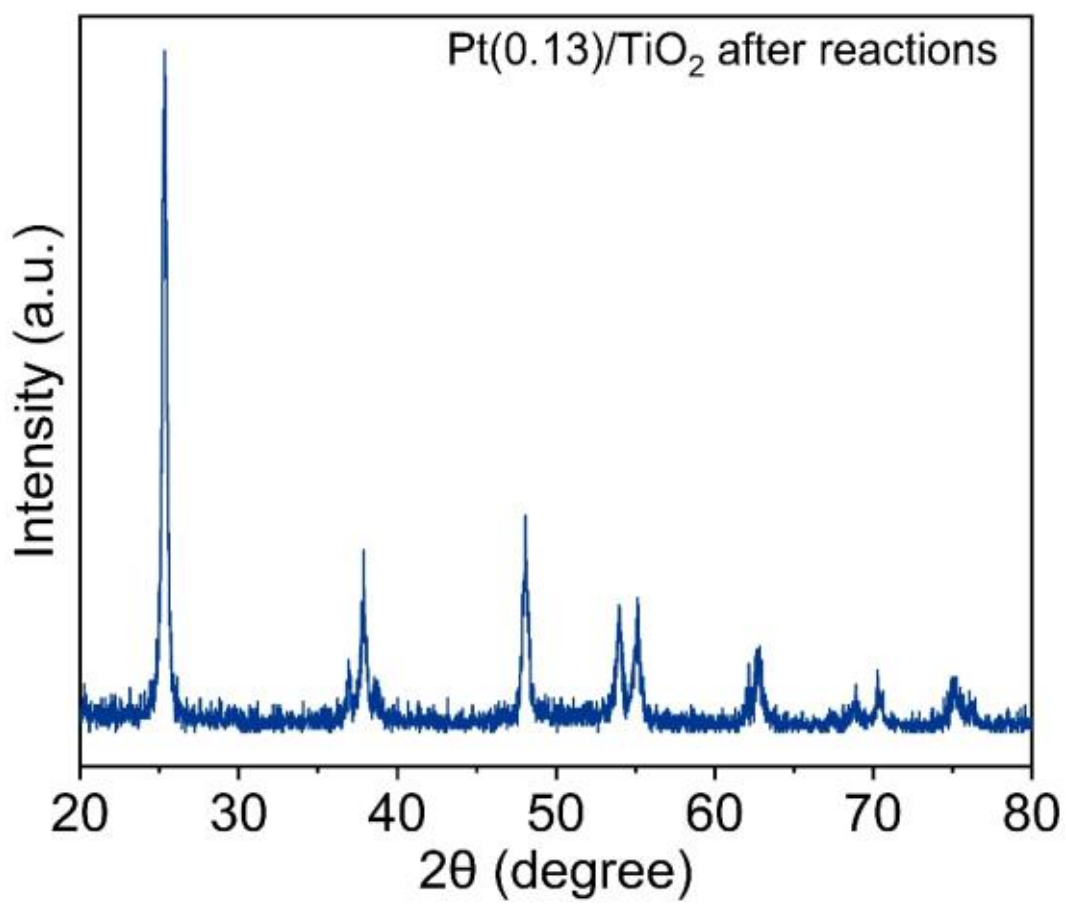

Fig. S20 | X-ray diffraction pattern of Pt(0.13)/TiO_2 photocatalyst after five reaction cycles.

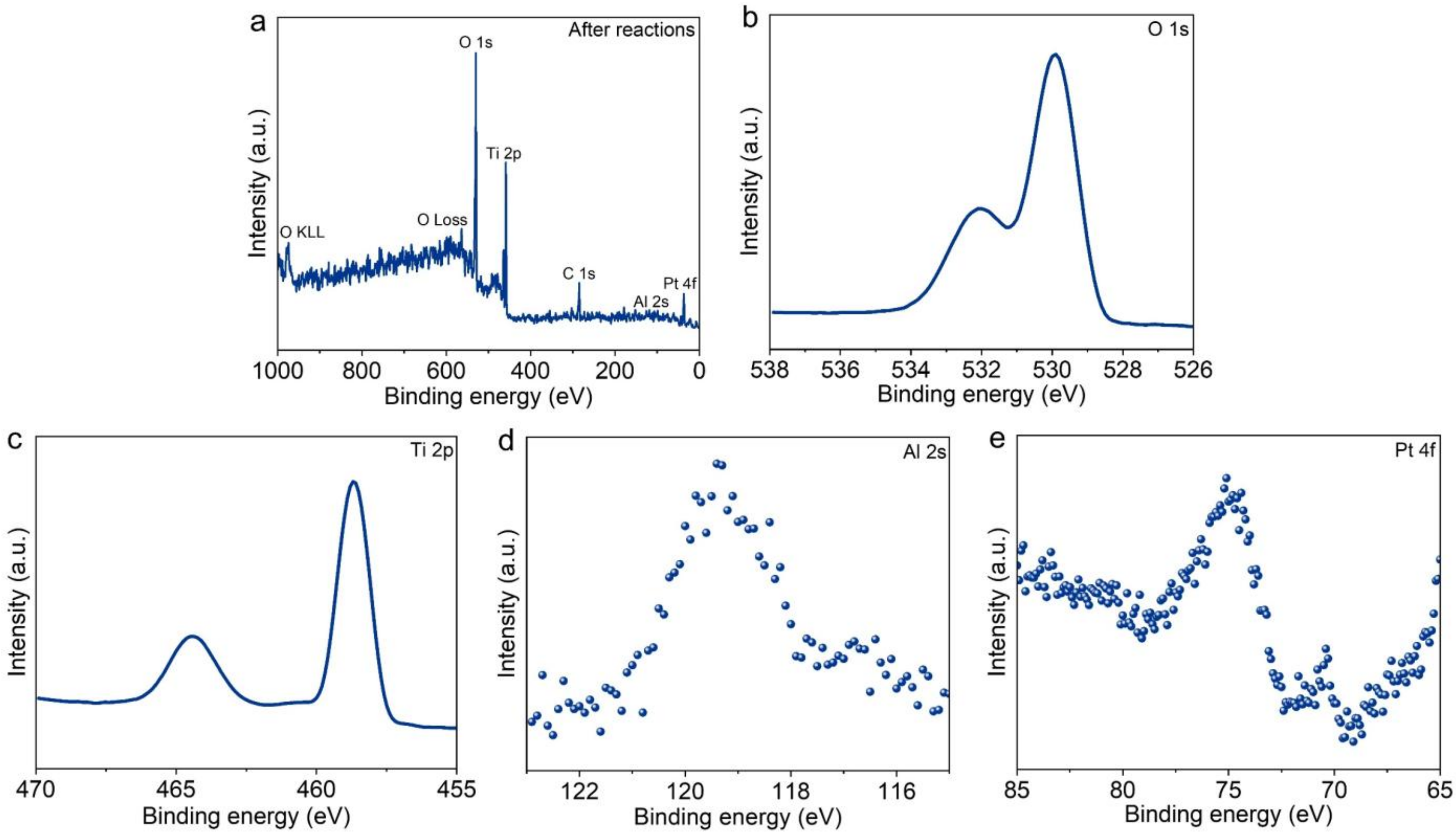

Fig. S21 | (**a**) XPS survey and high-resolution XPS spectra of (**b**) O 1s, (**c**) Ti 2p, (**d**) Al 2s, and (**e**) Pt 4f for Pt(0.13)/TiO_2 photocatalyst after five reaction cycles.

XPS analysis of the Pt(0.13)/TiO_2 photocatalyst after five reaction cycles reveal that the elemental composition remained largely unchanged, indicating excellent structural stability. No significant loss of Pt was observed, confirming that the active metal species remained immobilized during the photocatalytic methane chlorination. Only trace amounts of residual Al were detected, likely originating from the insoluble $Al(OH)_3$. These results demonstrate the catalyst's outstanding physicochemical stability under reaction conditions.

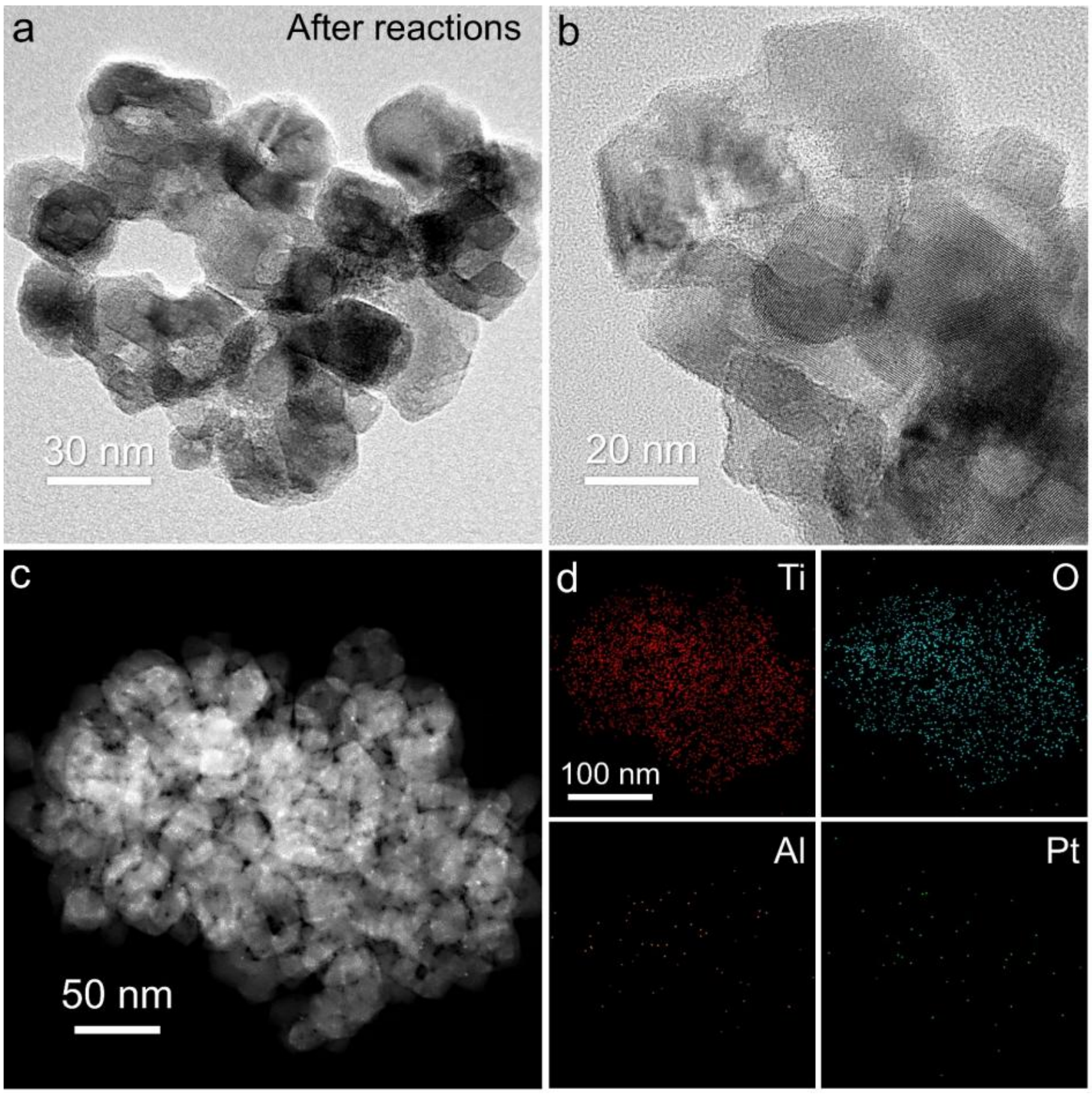

Fig. S22 | (**a**) TEM, (**b**) high-resolution TEM, (**c**) dark field, and (**d**) elements mapping images of $Pt(0.13)/TiO_2$ photocatalyst after five reaction cycles.

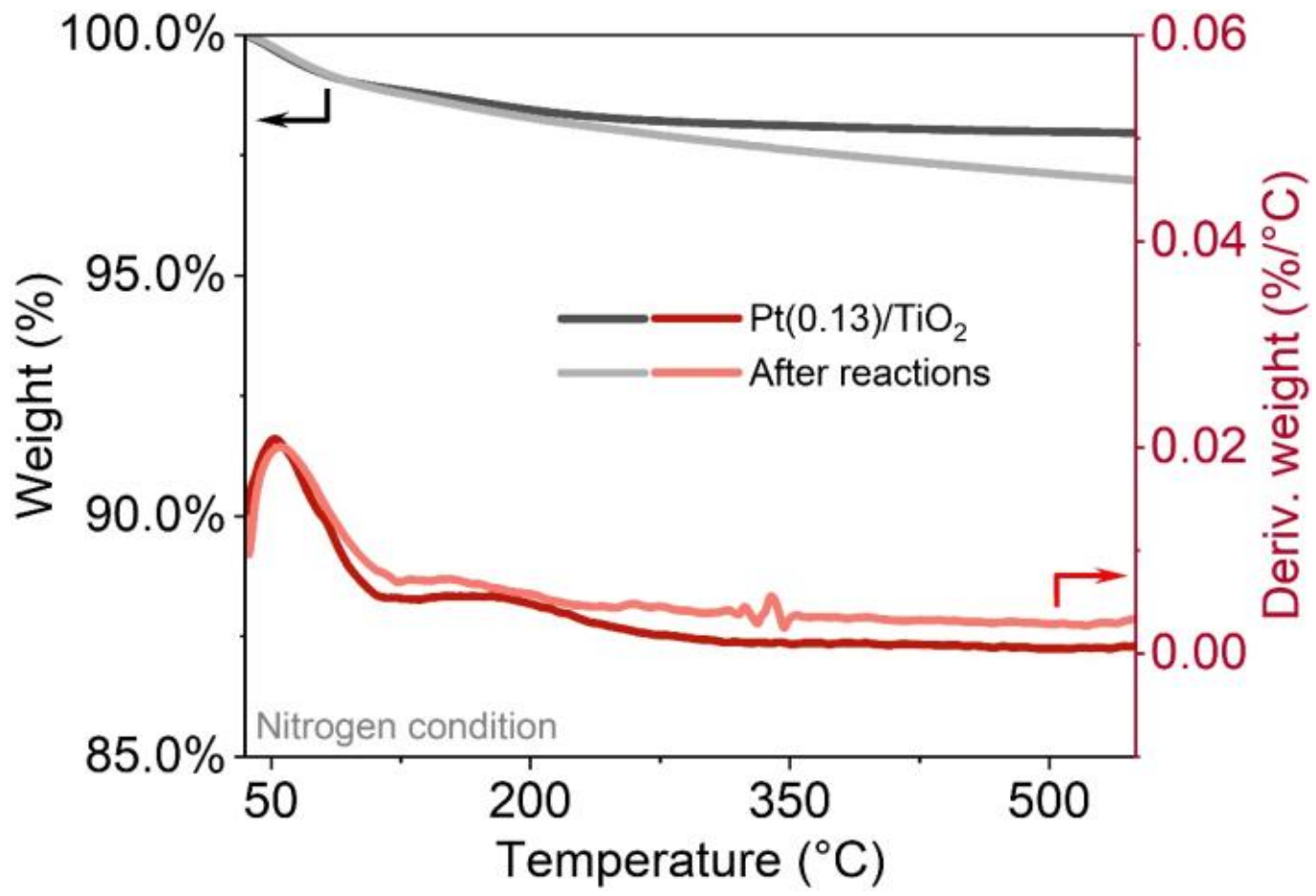

Fig. S23 | TGA and DTG curves of Pt(0.13)/TiO_2 before and after five reaction cycles.

TGA revealed characteristic $Al(OH)_3$ signals at 340°C,[6] suggesting minimal accumulation of insoluble Al species on recycled photocatalyst.

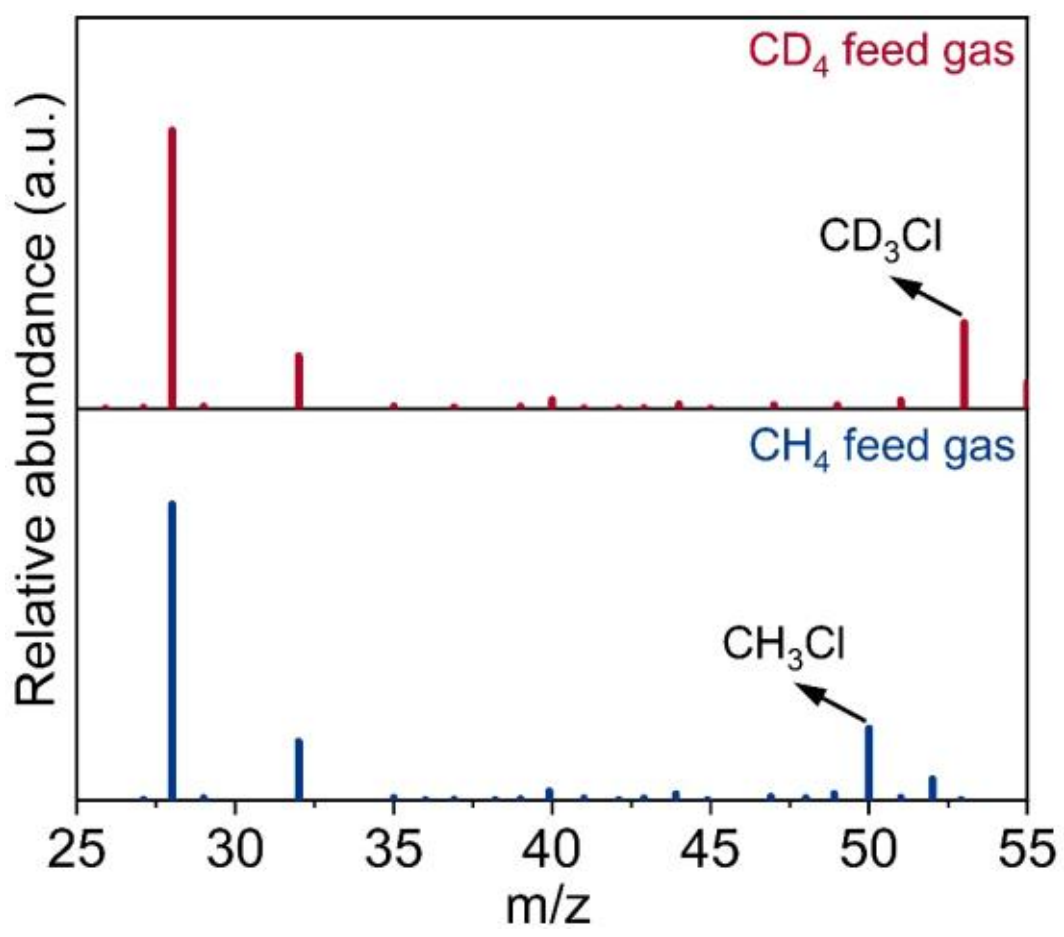

Fig. S24 | Gas chromatography-mass spectrometry (GC-MS) spectra of chloromethane using CH_4 or CD_4 as feed gas.

Isotopic labelling experiments using GC-MS with CH_4 and deuterated methane (CD_4) as reactants revealed distinct changes in m/z values, shifting from 50 (corresponding to CH_3Cl) to 53 (corresponding to CD_3Cl). This confirmed the conversion of methane to chloromethane.

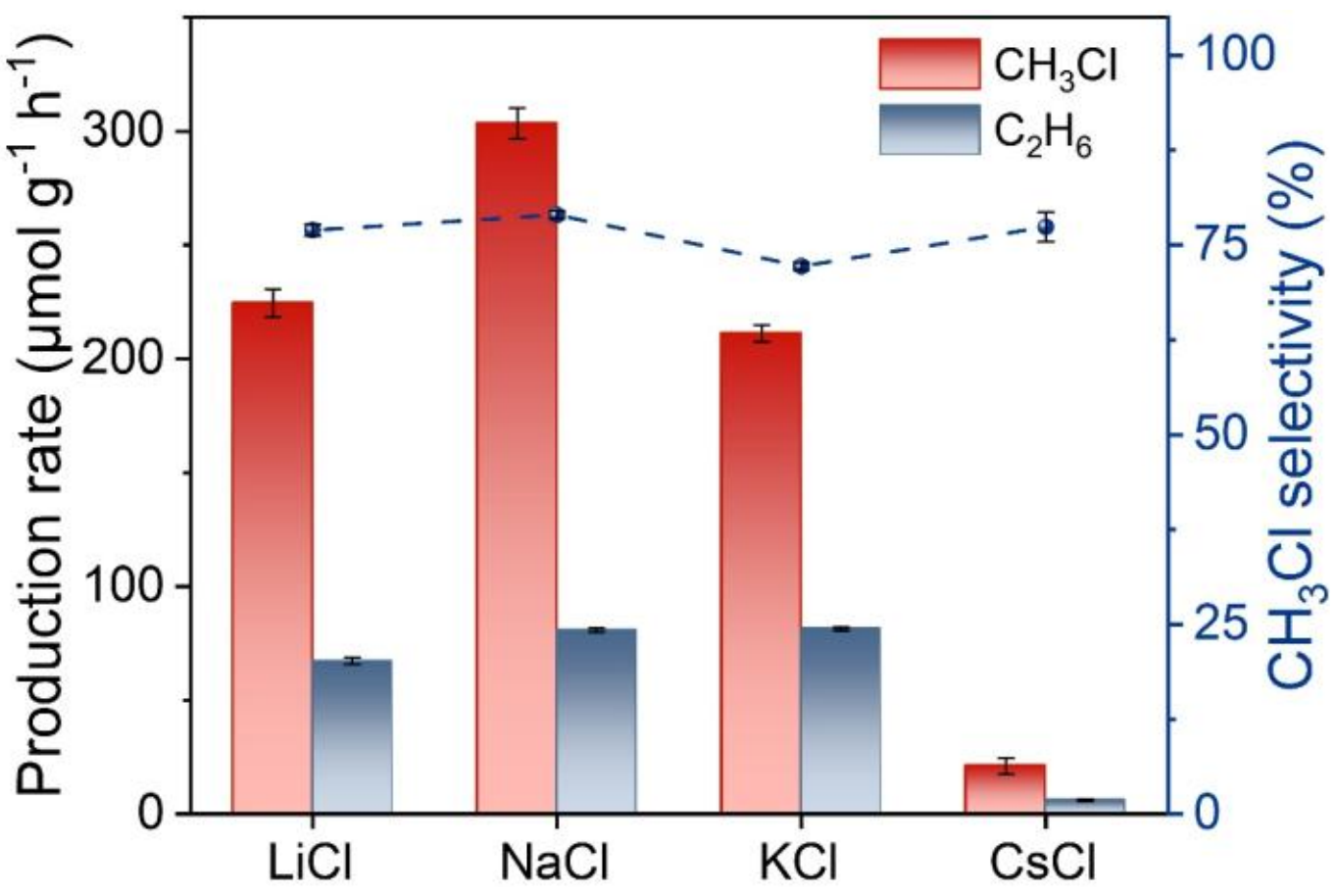

Fig. S25 | Product yields and CH_3Cl selectivity over Pt(0.13)/TiO_2 photocatalyst in different alkali-metal chlorides solution (10 mg/mL).

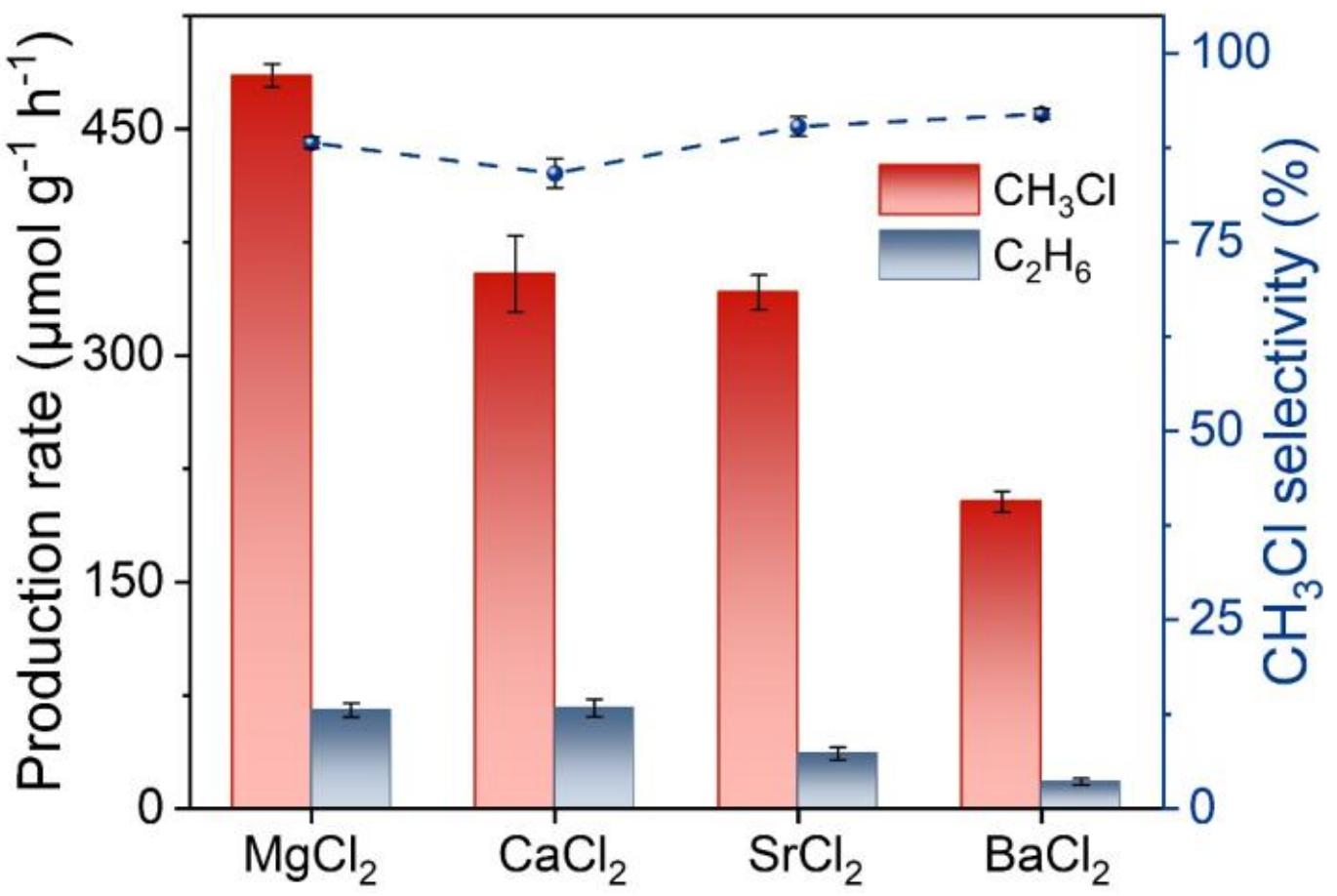

Fig. S26 | Product yields and CH_3Cl selectivity over Pt(0.13)/TiO_2 photocatalyst in different alkaline-earth-metal chlorides solution (10 mg/mL).

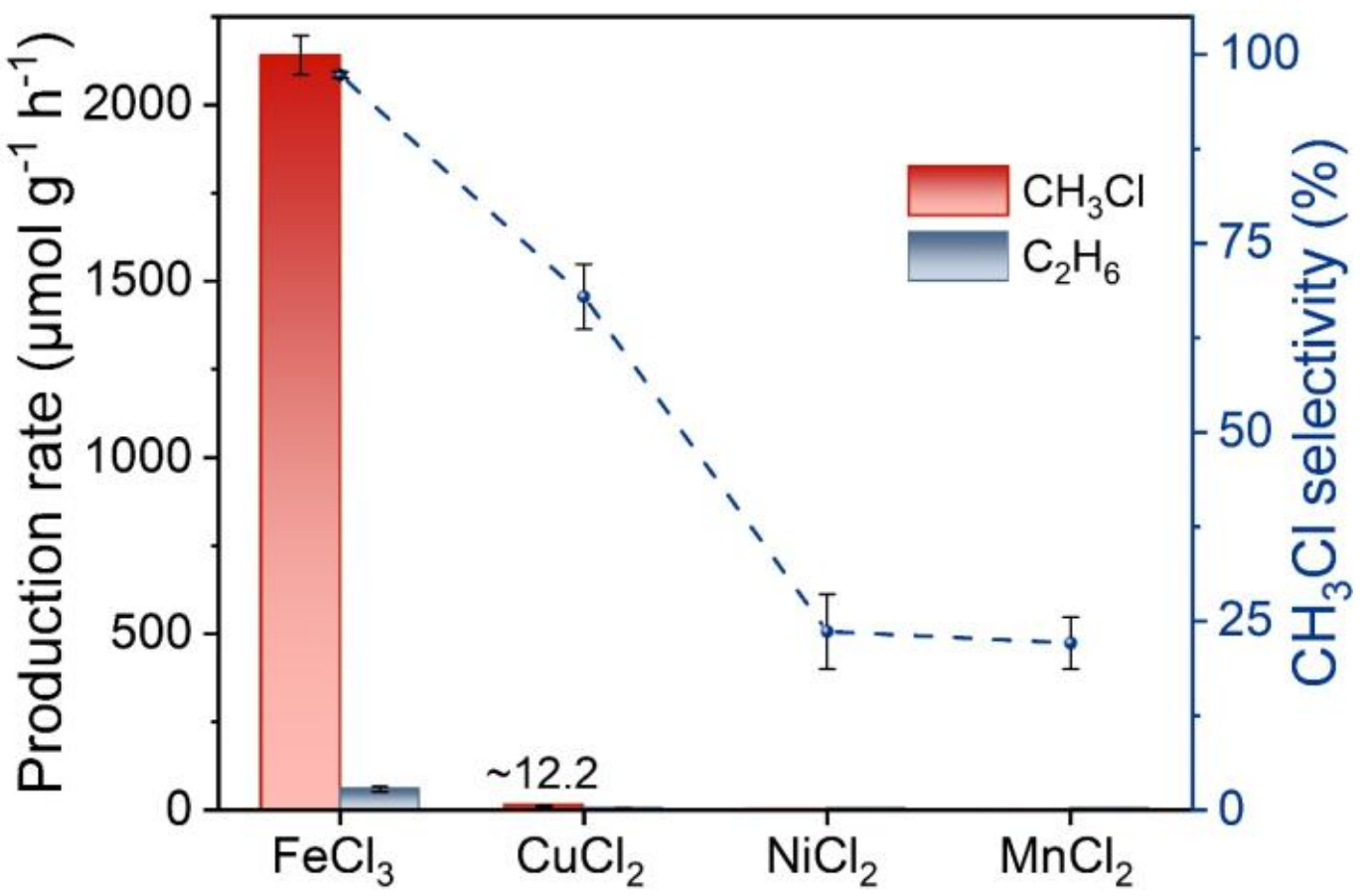

Fig. S27 | Product yields and CH_3Cl selectivity over Pt(0.13)/TiO_2 photocatalyst in different transition-metal chlorides solution (10 mg/mL).

Negligible CH_3Cl yields were observed in $CuCl_2$, $NiCl_2$ and $MnCl_2$ solutions, likely due to the formation of their insoluble hydroxides that block active sites and severely hinder the CH_4 chlorination reaction.

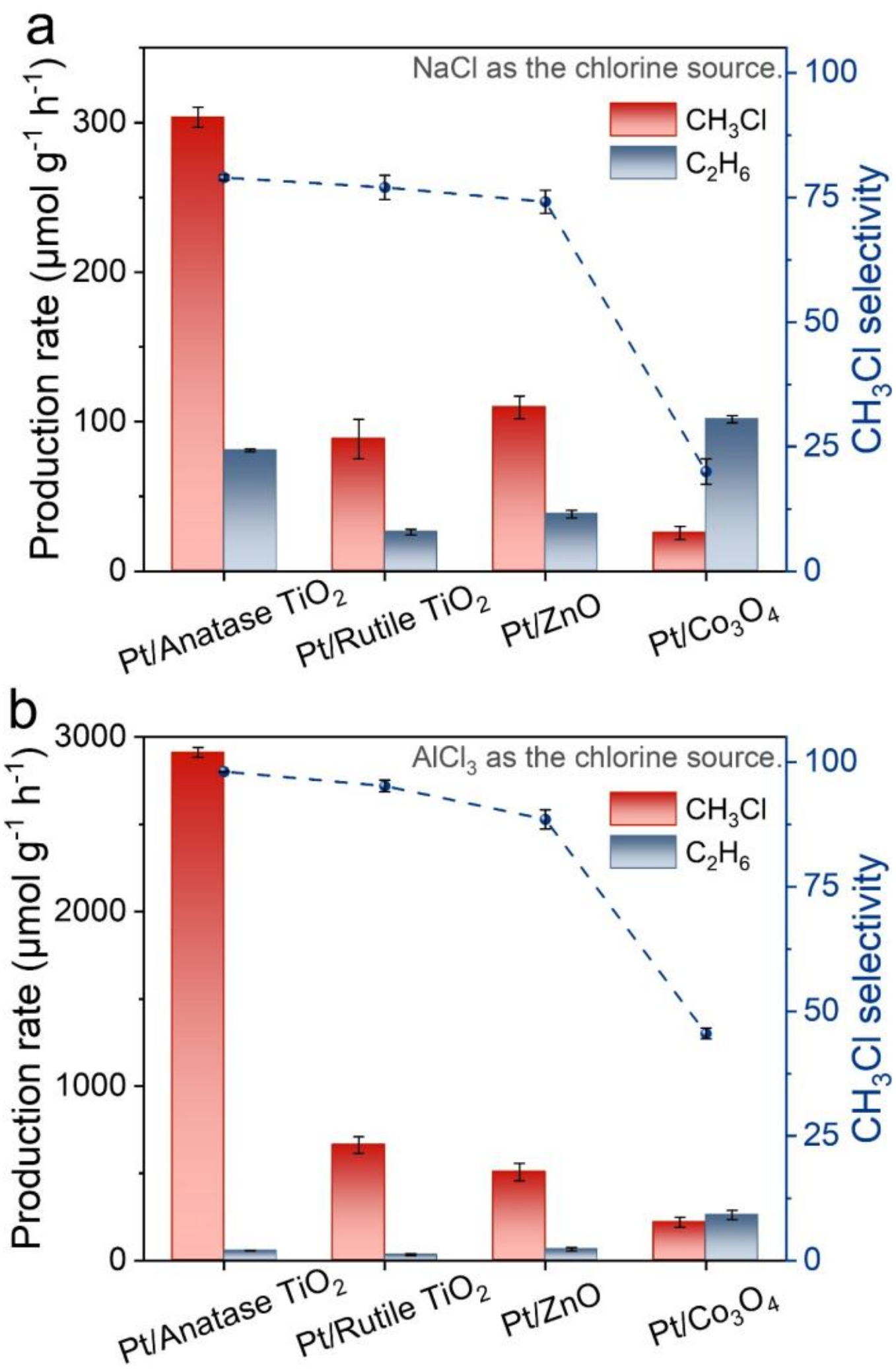

Fig. S28 | Product yields and CH_3Cl selectivity over supported photocatalysts with different oxide supports (Pt/Anatase TiO_2, Pt/Rutile TiO_2, Pt/ZnO, Pt/Co_3O_4) using NaCl or $AlCl_3$ as chlorine sources.

Different oxide supports were selected based on their reported photocatalytic activity toward CH_4 activation and their distinct electronic structures. As a result, the intrinsic CH_3Cl formation rates vary significantly across supports under NaCl conditions (e.g., Pt/anatase TiO_2 ~303 μmol g^{-1} h^{-1} vs. substantially lower activity on rutile TiO_2, ZnO, and Co_3O_4). Upon introducing $AlCl_3$, however, CH_3Cl formation is markedly enhanced across all systems (e.g., up to ~2912 μmol g^{-1} h^{-1} on Pt/anatase TiO_2), indicating that the promotion effect is not limited to a specific semiconductor but arises from a general interfacial regulation mechanism.

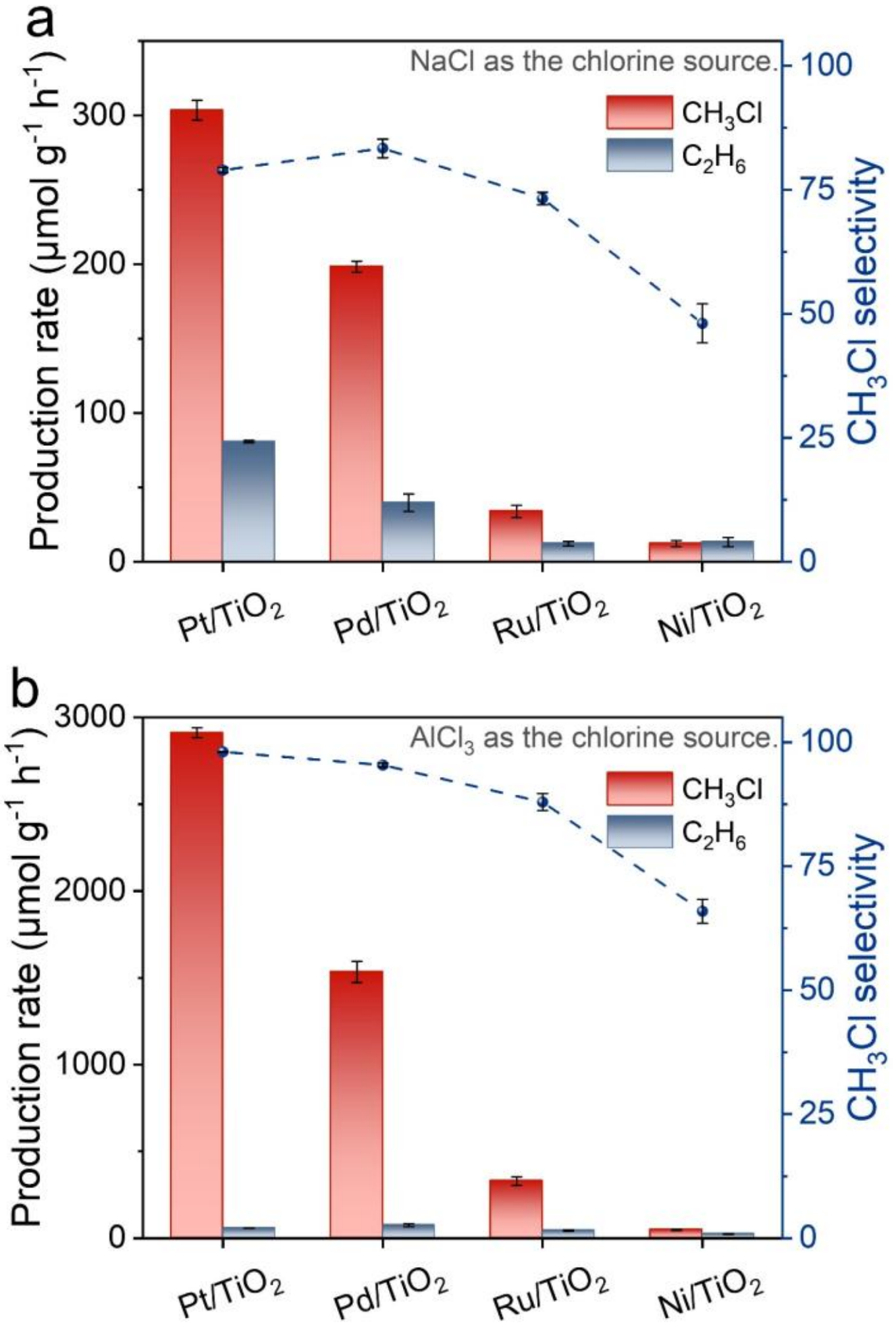

Fig. S29 | Product yields and CH_3Cl selectivity over TiO_2-supported photocatalysts with different metal cocatalysts (Pt, Pd, Ru, Ni) using NaCl or $AlCl_3$ as chlorine sources.

A range of metal cocatalysts (Pt, Pd, Ru, and Ni) was examined to evaluate the generality of the Lewis-acid-assisted effect. The intrinsic CH_3Cl formation rates vary with cocatalyst identity (e.g., Pt ~303 μmol g^{-1} h^{-1}, Pd ~198 μmol g^{-1} h^{-1} under NaCl conditions), reflecting differences in C–H activation capability. Upon the addition of $AlCl_3$, all systems exhibit a pronounced increase in CH_3Cl production (e.g., Pt ~2912 μmol g^{-1} h^{-1}, Pd ~1534 μmol g^{-1} h^{-1}), demonstrating that the promotional effect of Lewis-acidic species is not confined to a specific cocatalyst, but instead originates from a common modulation of the interfacial reaction environment.

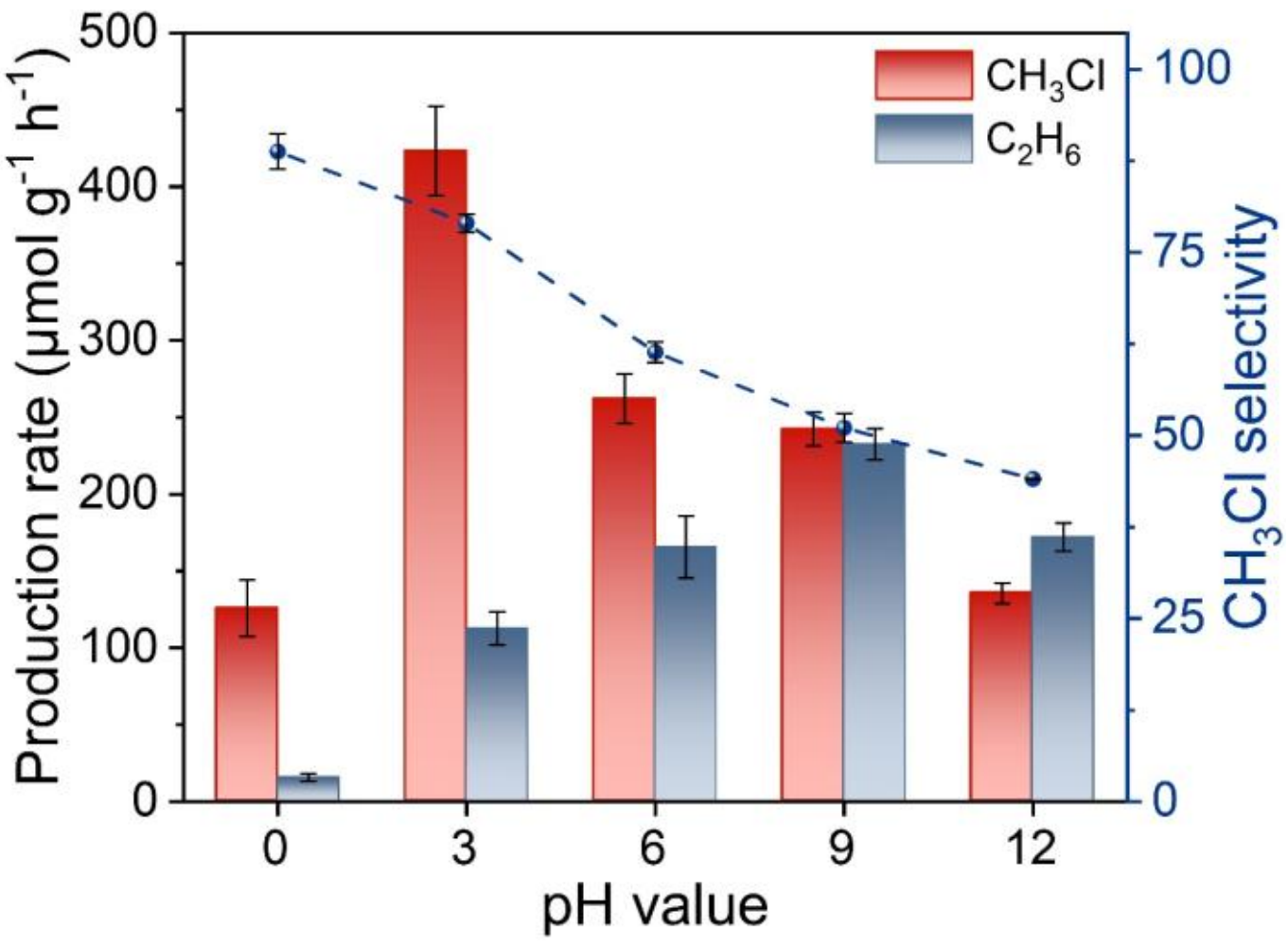

Fig. S30 | Product yields and CH_3Cl selectivity over Pt(0.13)/TiO_2 photocatalyst in HCl/NaCl mixed solution (total Cl^- concentration = 1 mM) as a function of pH value.

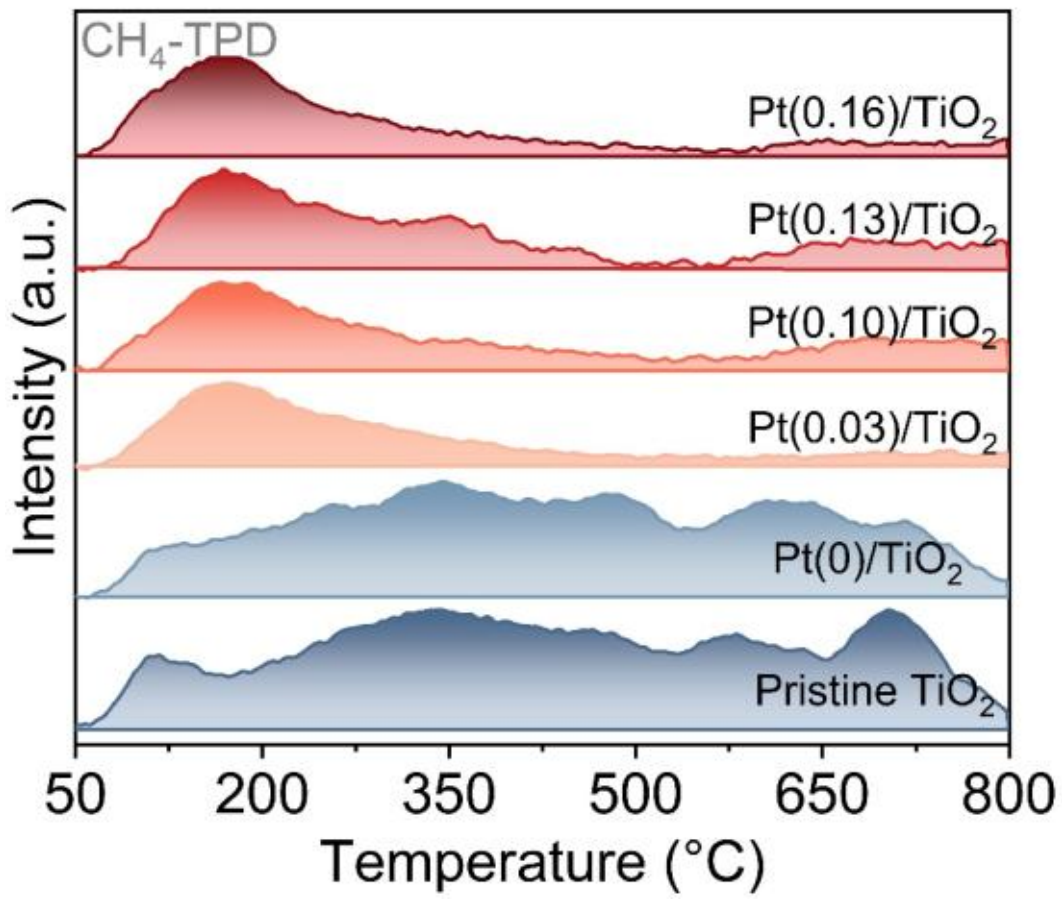

Fig. S31 | CH_4 temperature programmed desorption (CH_4-TPD) for pristine TiO_2 and Pt(x)/TiO_2.

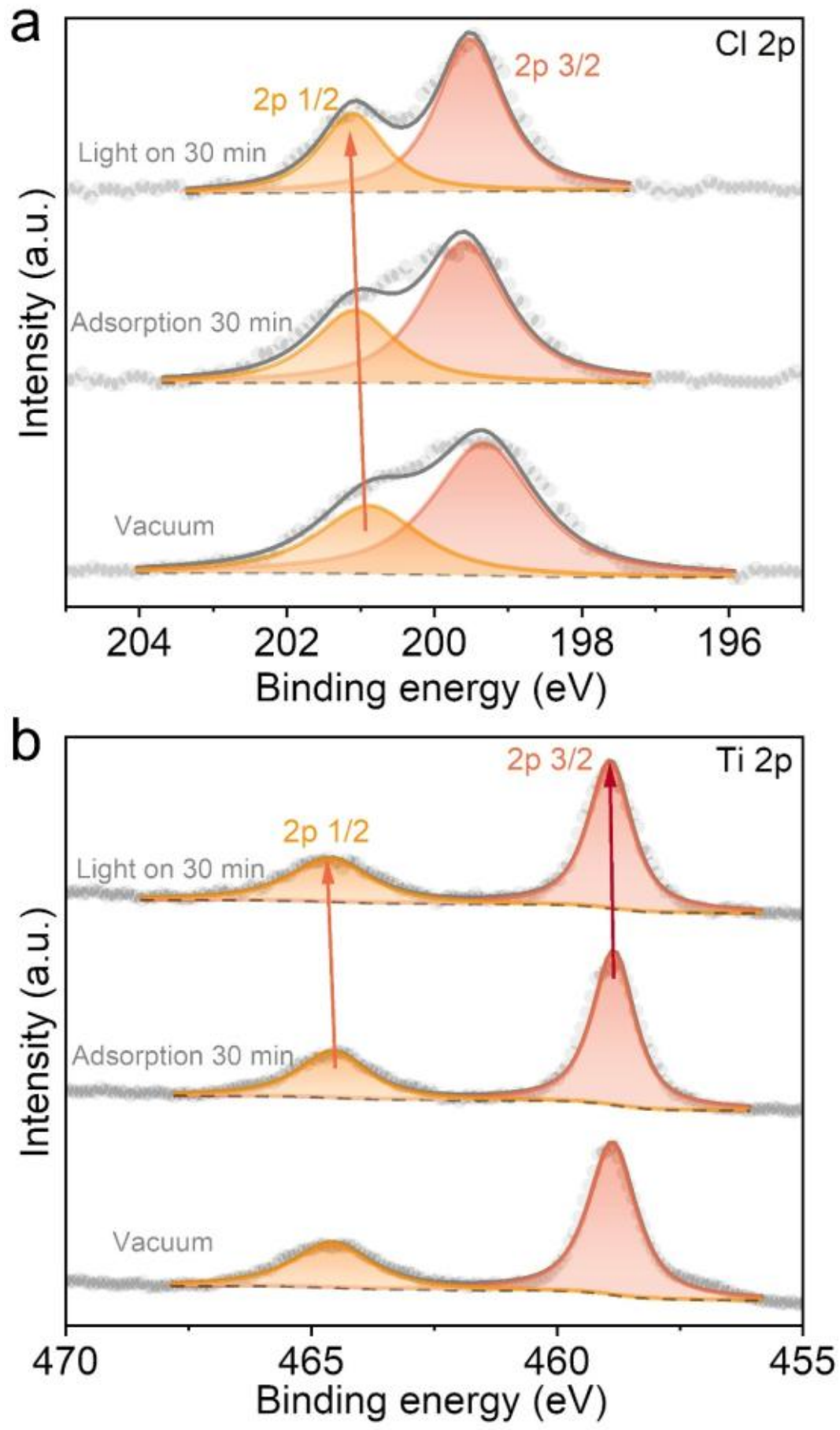

Fig. S32 | High-resolution XPS spectra of (**a**) Cl 2p and (**b**) Ti 2p for Pt(0.13)/TiO_2 photocatalyst with adding of NaCl in ultrahigh vacuum under dark condition, and upon exposure to 0.45 mbar of moist CH_4 in dark and under light illumination.

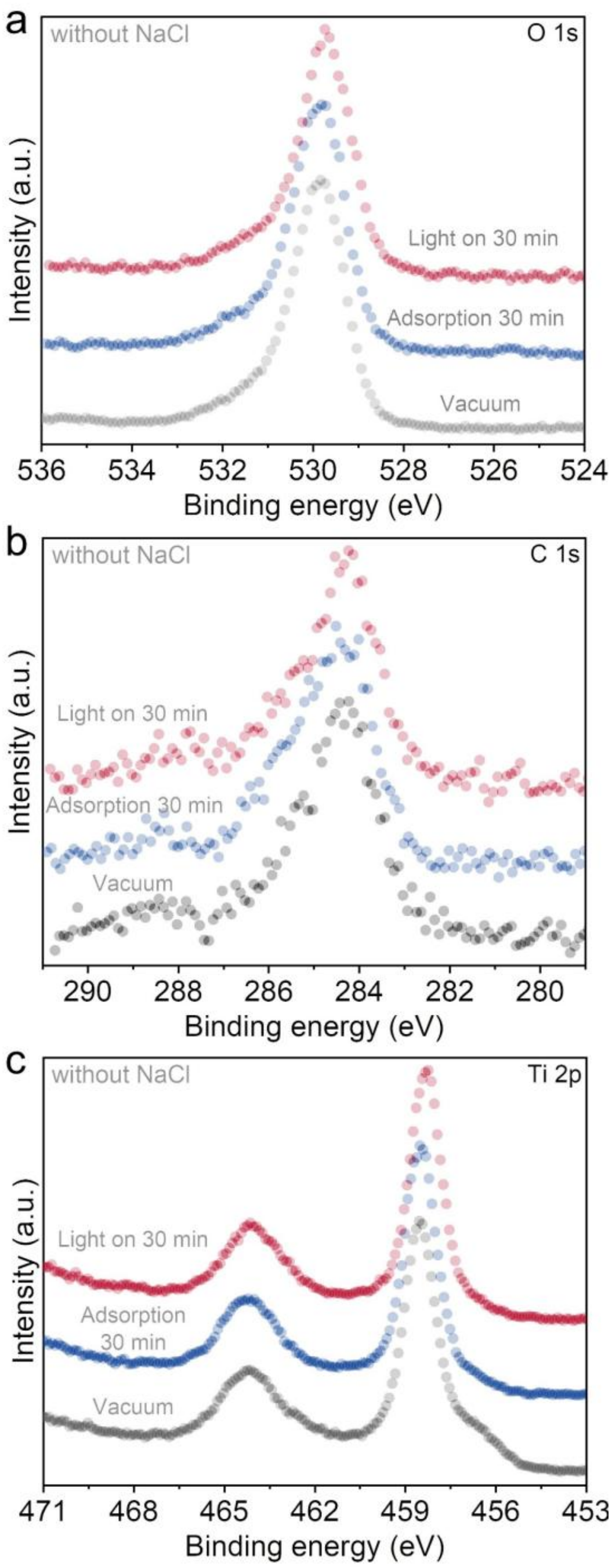

Fig. S33 | High-resolution XPS spectra of (**a**) O 1s, (**b**) C 1s, and (**c**) Ti 2p for Pt(0.13)/TiO_2 photocatalyst without adding of NaCl in ultrahigh vacuum under dark condition, and upon exposure to 0.45 mbar of moist CH_4 in dark and under light illumination.

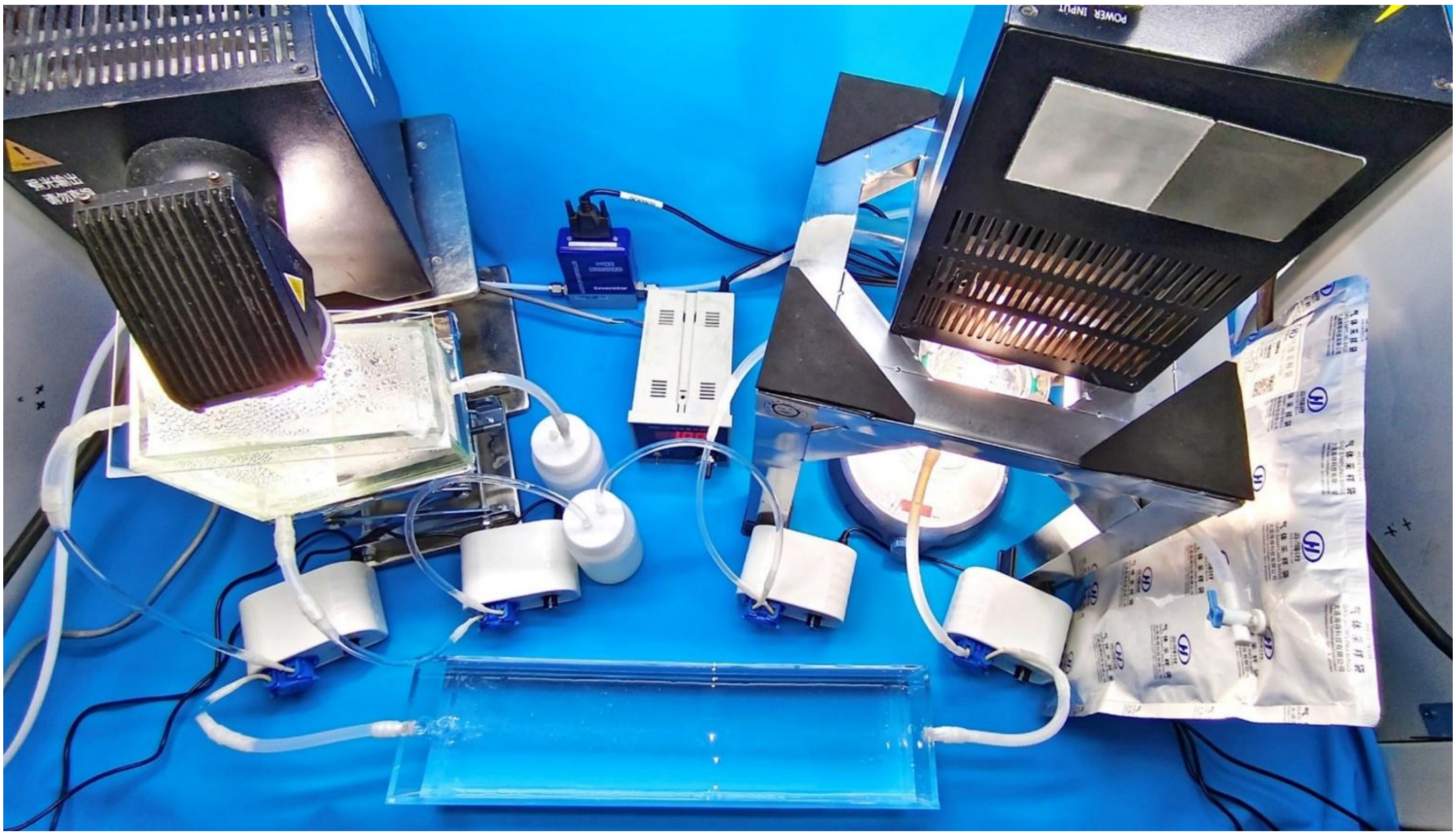

Fig. S34 | Top-view photograph of the tandem system comprising a solar steam generator and a photocatalytic CH_4 conversion flow reactor.

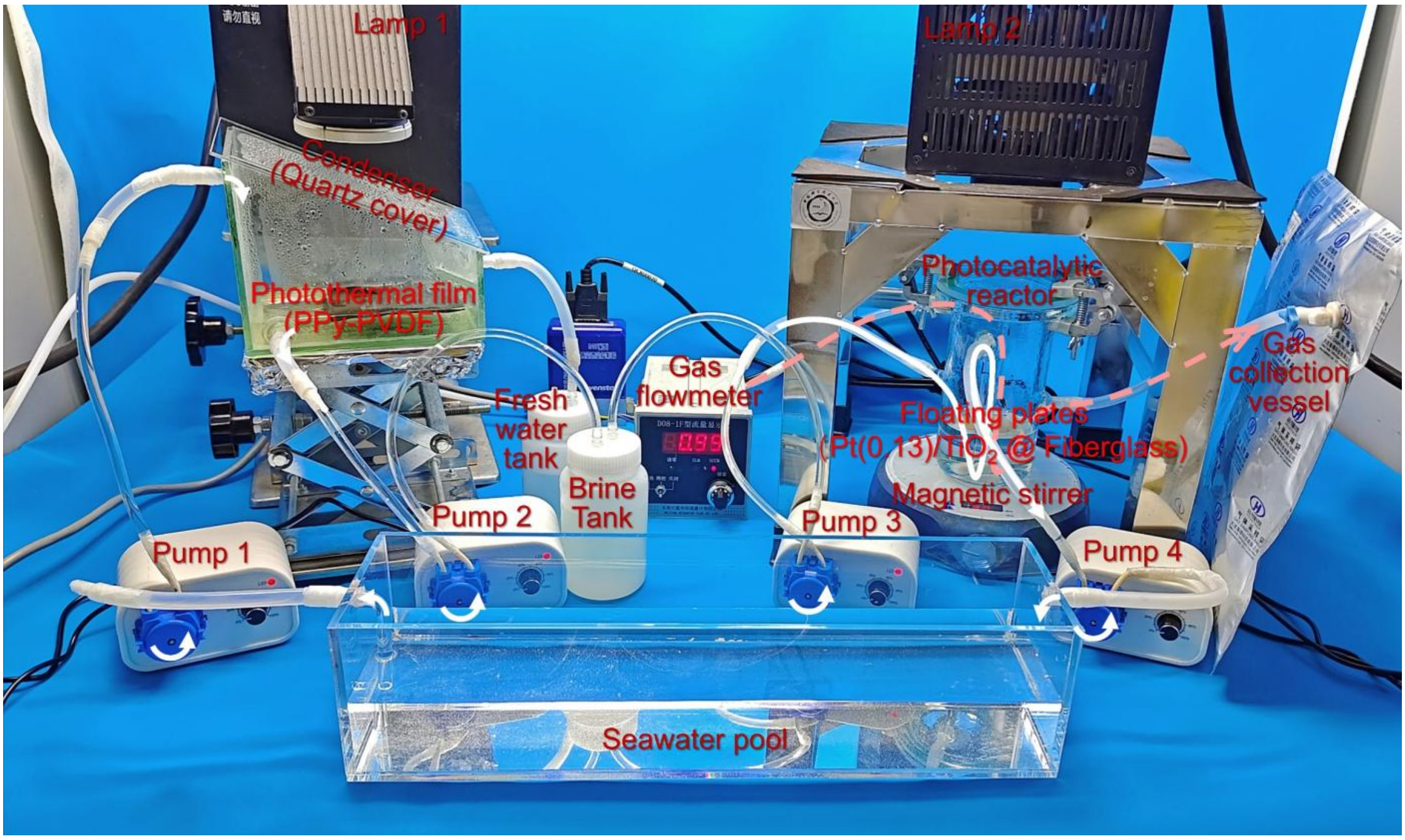

Fig. S35 | Photograph showing the layout and connections of the integrated tandem system.

A floating PPy–PVDF photothermal membrane was used to evaporate seawater in the SSG system. The resulting vapor condensed on the quartz cover above and was subsequently collected in a freshwater tank. Meanwhile, the floatable Pt(0.13)/TiO_2@fiberglass plates were employed to utilize the brine byproduct from the SSG system for photocatalytic CH_4 chlorination in a dedicated reactor. A gas flowmeter and four pumps were arranged to ensure continuous flow throughout the entire tandem system.

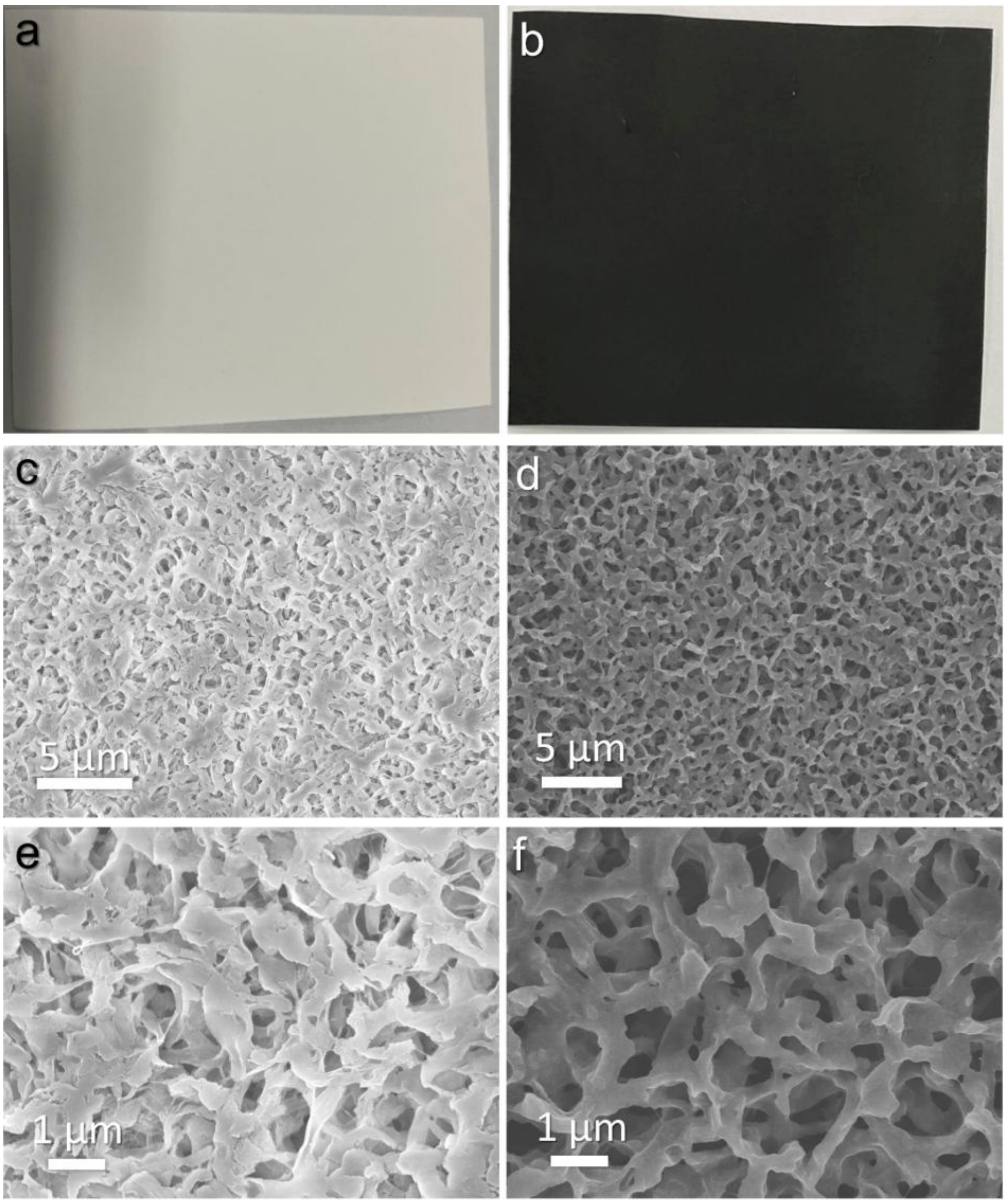

Fig. S36 | Optical photograph and FESEM images of (**a, d, e**) PVDF membrane and (**b, d, f**) PPy–PVDF membrane.

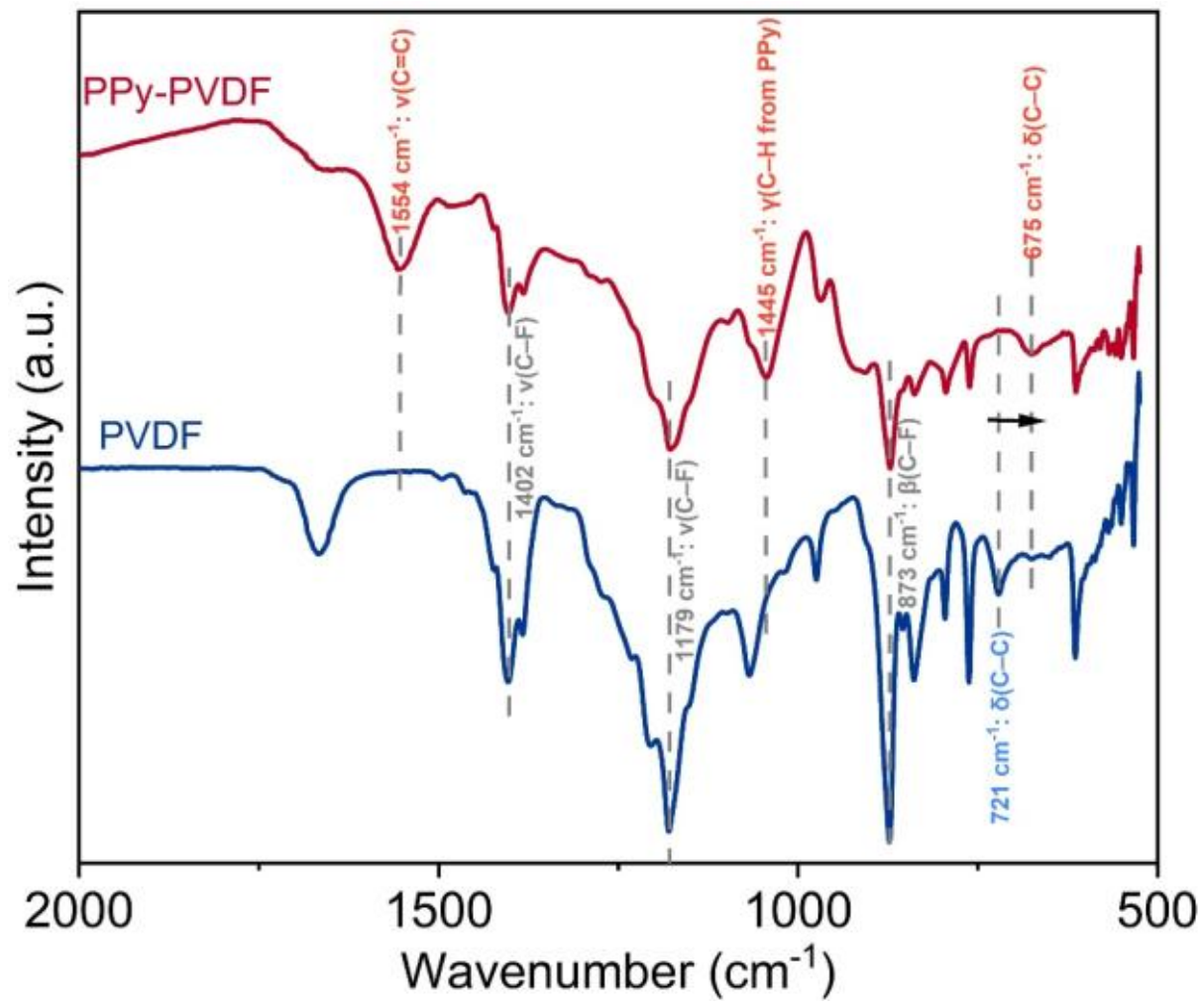

Fig. S37 | Fourier transform infrared (FTIR) spectra of PVDF membrane and PPy–PVDF membrane.

The successful construction of the PPy–PVDF membrane was verified by FTIR spectroscopy. Characteristic peaks of PVDF were clearly retained, including the C–F stretching vibrations at 1402 and 1179 cm^{-1}, and the β-phase characteristic vibration at 873 cm^{-1}. Compared to the δ (C–F) deformation band observed at 721 cm^{-1} in pristine PVDF, a noticeable shift to 675 cm^{-1} was observed in the PPy–PVDF membrane, suggesting surface functionalization and possible interaction between PPy and the PVDF matrix.[7] Furthermore, the presence of distinct absorption bands at 1554 and 1445 cm^{-1}, corresponding to the C=C stretching and out-of-plane C–H deformation of polypyrrole, respectively, further confirmed the successful incorporation of PPy onto the PVDF surface.

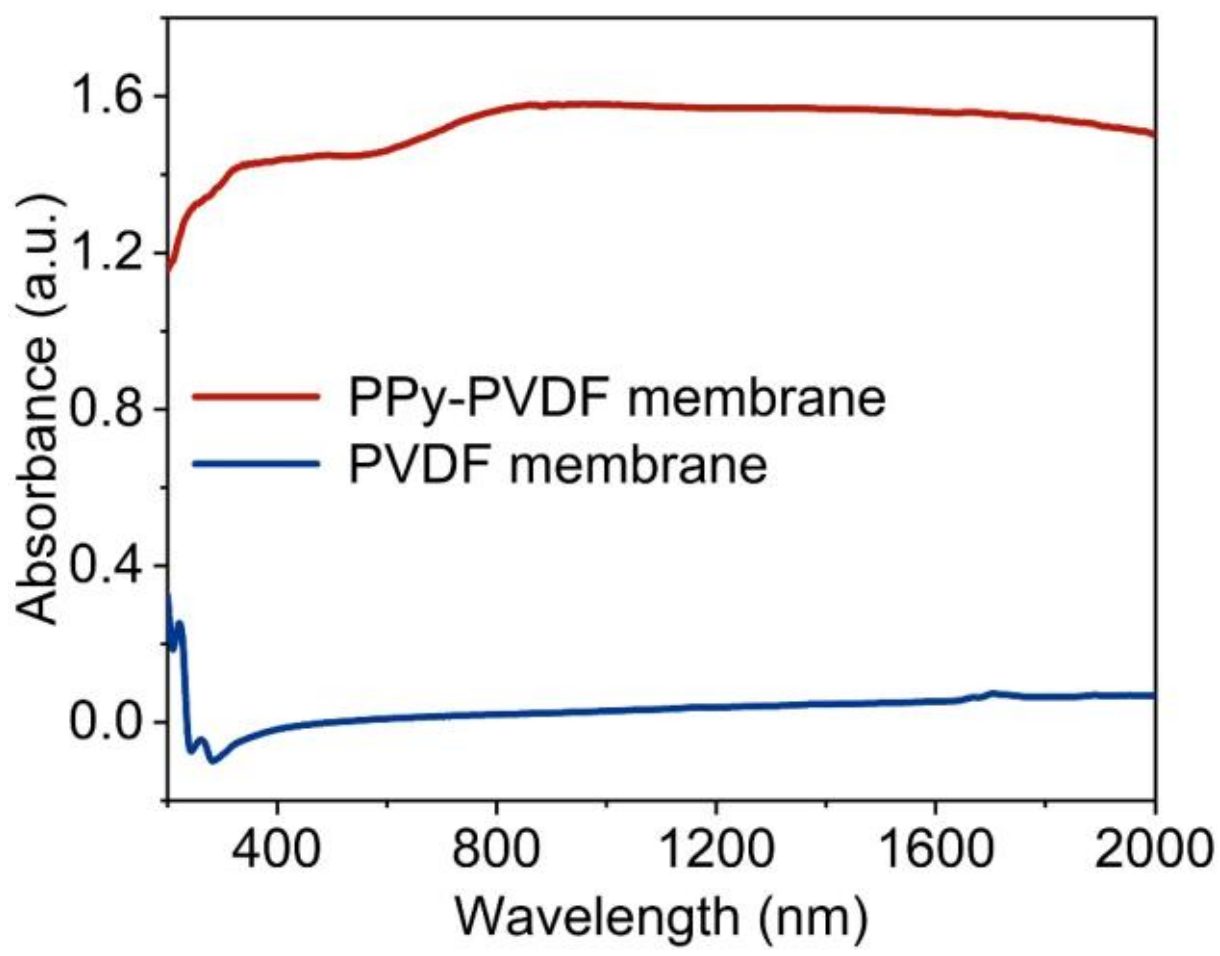

Fig. S38 | Ultraviolet-visible diffuse reflectance spectra (UV-vis DRS) of PVDF membrane and PPy–PVDF membrane.

UV-vis DRS indicate that the surface functionalization of the PVDF membrane with in situ synthesized polypyrrole (PPy) significantly enhances light absorption across the full spectral range, thereby endowing photothermal conversion capability to the resulted PPy–PVDF membrane.

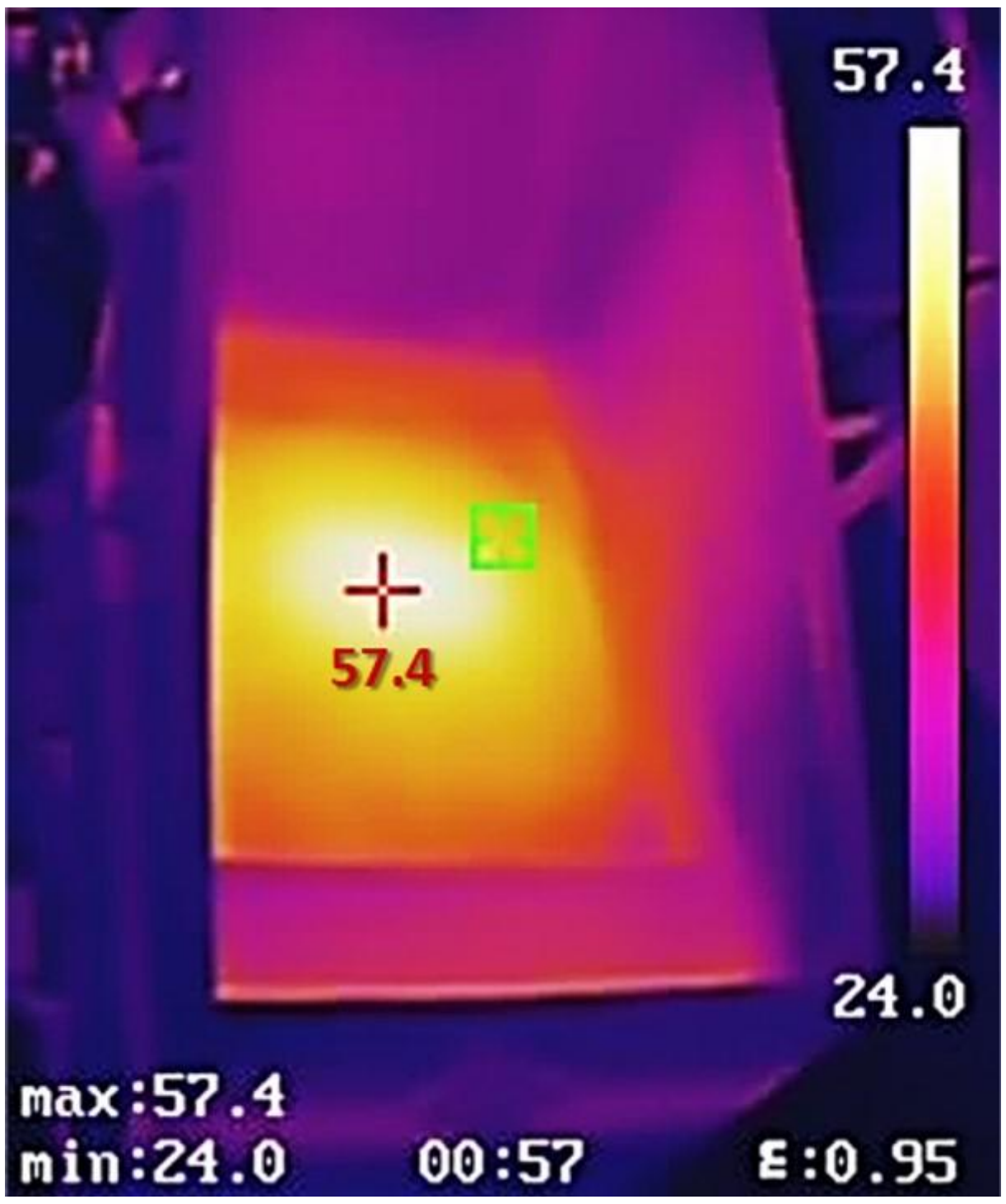

Fig. S39 | Infrared thermal image of the seawater evaporator.

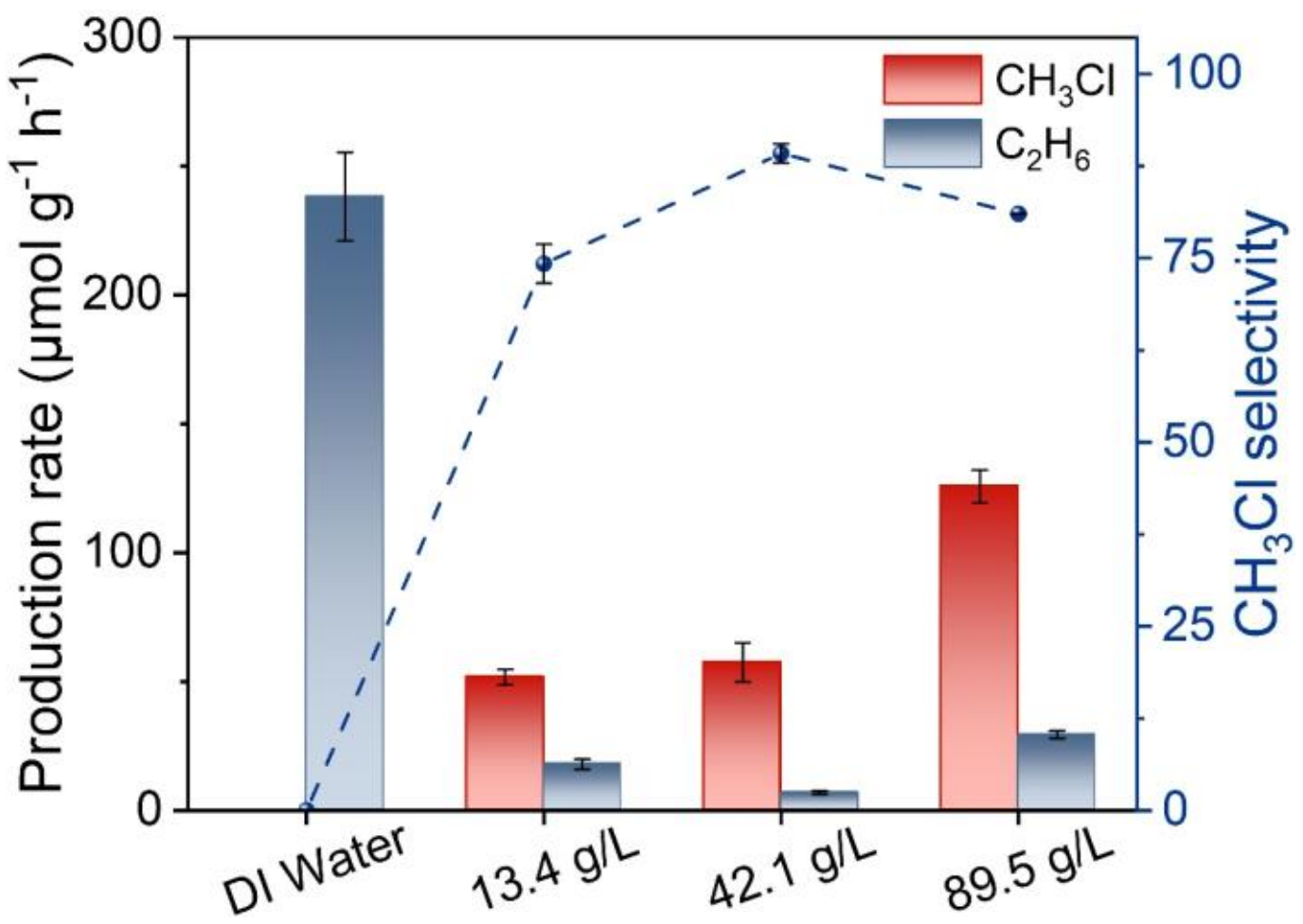

Fig. S40 | Product yields and CH_3Cl selectivity over Pt(0.13)/TiO_2 photocatalyst with concentrated seawater in different stages.

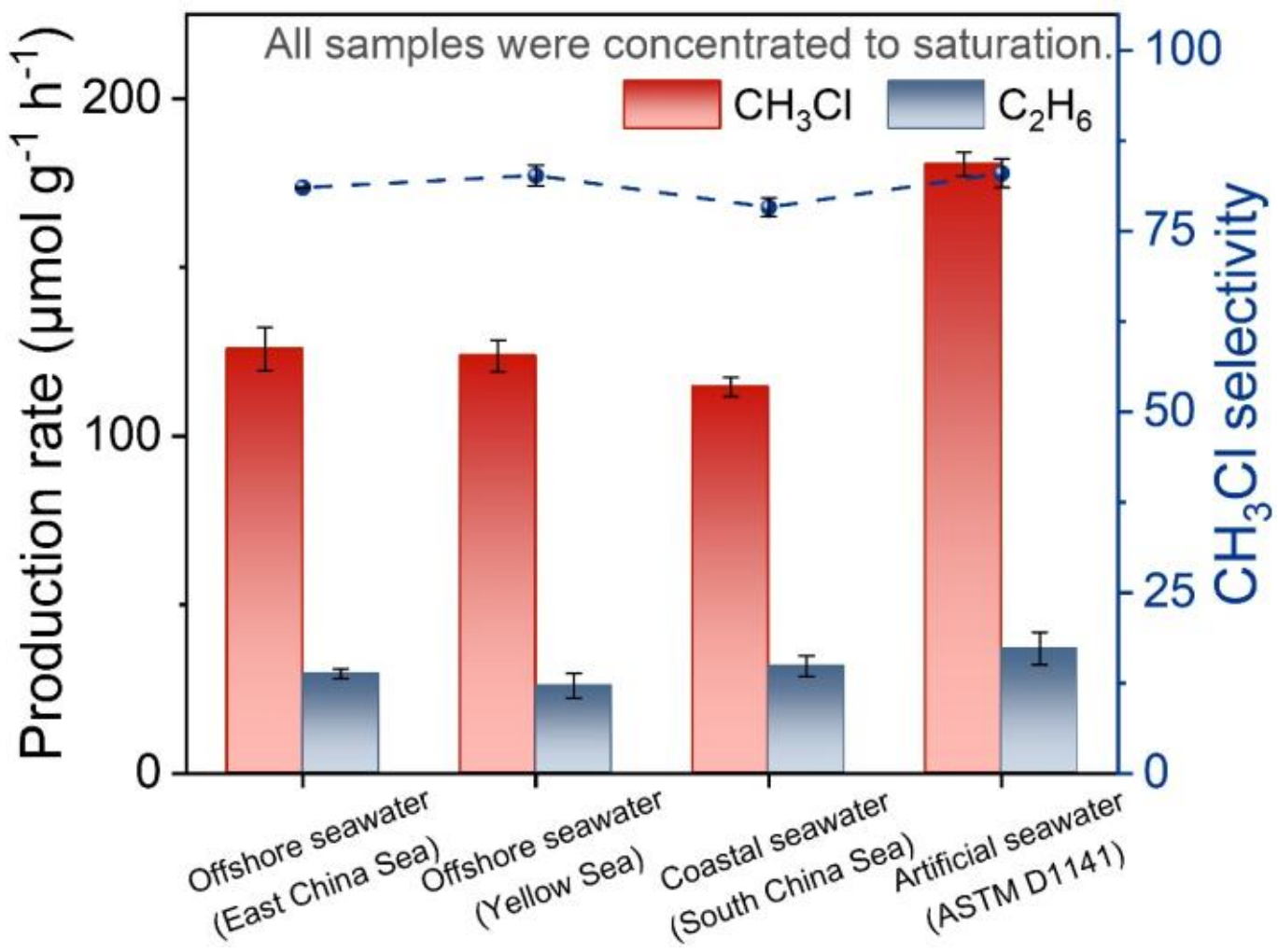

Fig. S41 | Product yields and CH_3Cl selectivity over Pt(0.13)/TiO_2 photocatalyst using different seawater samples.

All seawater samples were concentrated to saturation prior to photocatalytic testing. Seawater collected from the East China Sea, Yellow Sea, and South China Sea was compared with ASTM D1141 artificial seawater under identical reaction conditions. Despite differences in geographic origin and composition, the natural seawater samples exhibited highly consistent CH_3Cl production rates and selectivities, indicating that the photocatalytic system is relatively tolerant toward natural seawater variability. In contrast, the artificial seawater, which primarily contains defined inorganic salts without naturally occurring organic species, afforded noticeably higher CH_3Cl production. This result suggests that trace impurities or reductive species present in real seawater may partially consume reactive radical intermediates and modestly suppress CH_4 chlorination efficiency, although the overall selectivity toward CH_3Cl remained largely preserved across different seawater sources. Unless otherwise specified, offshore seawater collected from the East China Sea was used in subsequent flow-reactor experiments.

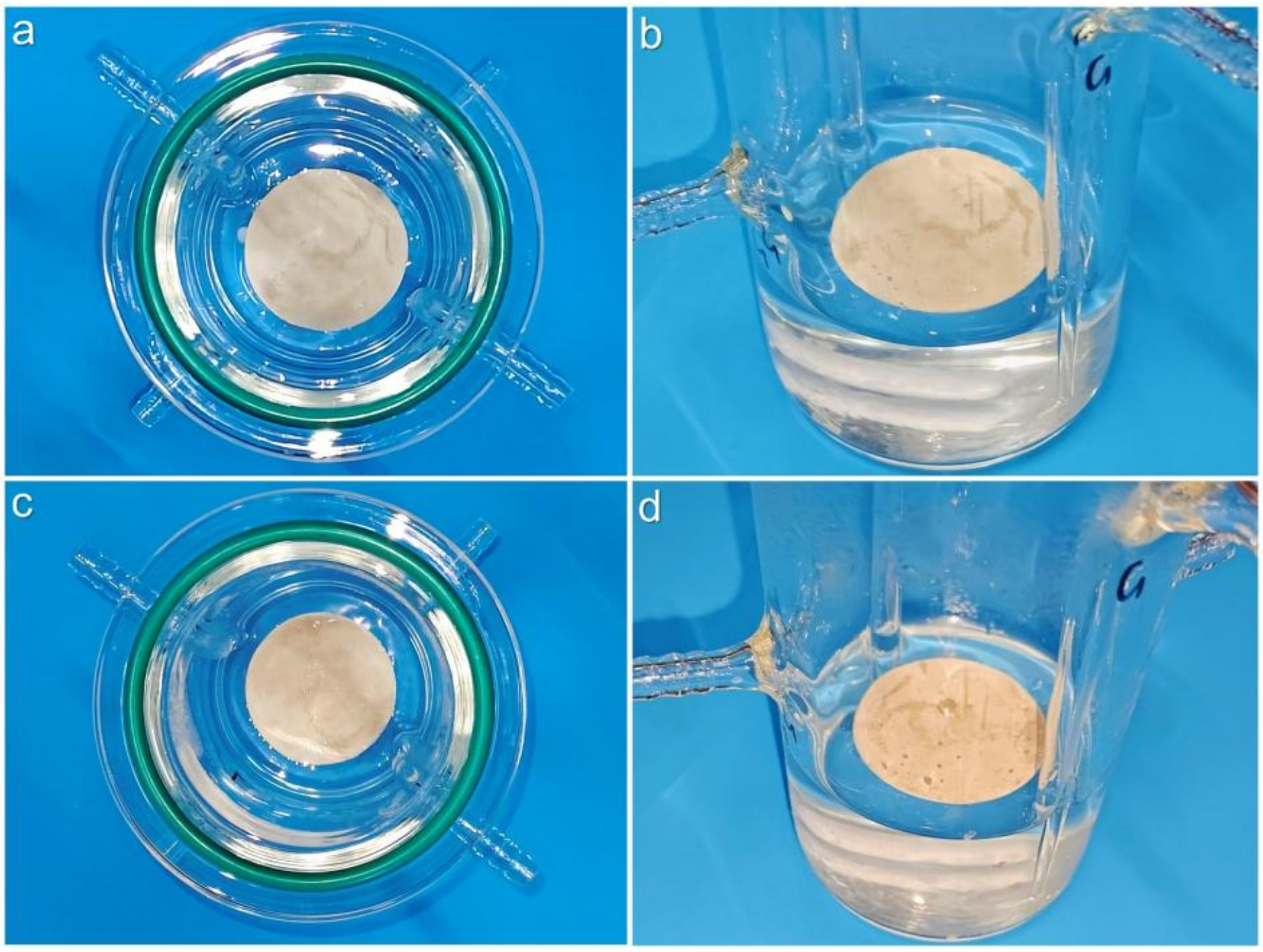

Fig. S42 | Photographs of the Pt(0.13)/TiO_2@fiberglass floatable plates (**a, b**) before and (**c, d**) after continuous flow reactions.

After long-term reactions, no visible salt crystallization was observed either on the surface of the Pt(0.13)/TiO_2@fiberglass floatable plates or within the interior of the photocatalytic reactor. This result demonstrates the excellent stability of the constructed gas-liquid-solid catalytic interface and the continuous flow system.

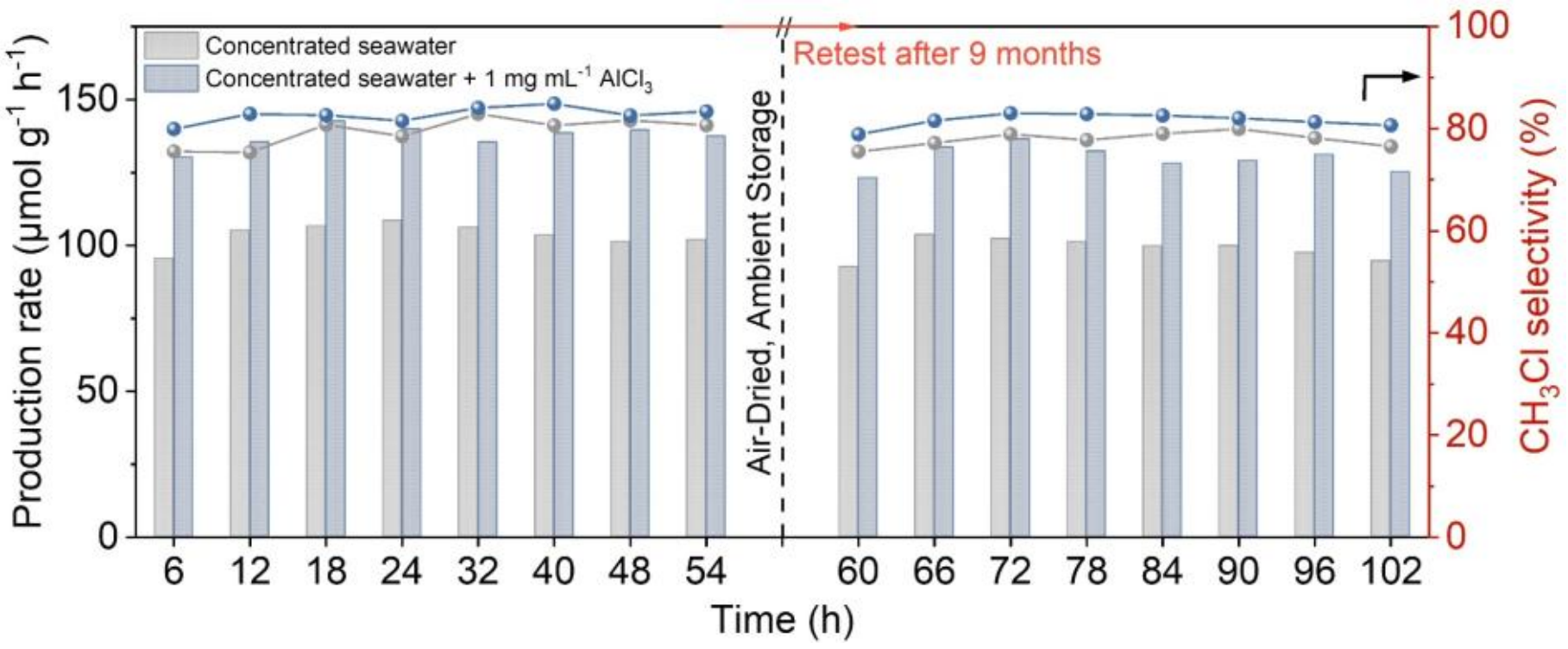

Fig. S43 | Product yields and CH_3Cl selectivity over Pt(0.13)/TiO_2@fiberglass floatable plate in the flow reactor, with CH_4 flow rate at 1.0 mL min^{-1} and brine flow rate at 2.0 mL min^{-1}, respectively. To ensure stable reactor operation, a 24 h conditioning period was performed before data collection. The volume of the gas chamber is ~350 mL, corresponding to a 6-hour reaction cycle. The diameter of the floatable plate is 47 mm, corresponding to a reaction area of 17.35 cm^2, loaded with 10 mg of Pt(0.13)/TiO_2 photocatalyst.

To comprehensively assess the stability of the Pt(0.13)/TiO_2@fiberglass floating plate, we rinsed and dried the plate after a period of continuous-flow operation, followed by ambient storage for 9 months before conducting a retest. The retesting results demonstrated over 95% retention of its performance, validating the floating plate's robust stability in both continuous and intermittent operation modes. This highlights its significant potential for practical application.

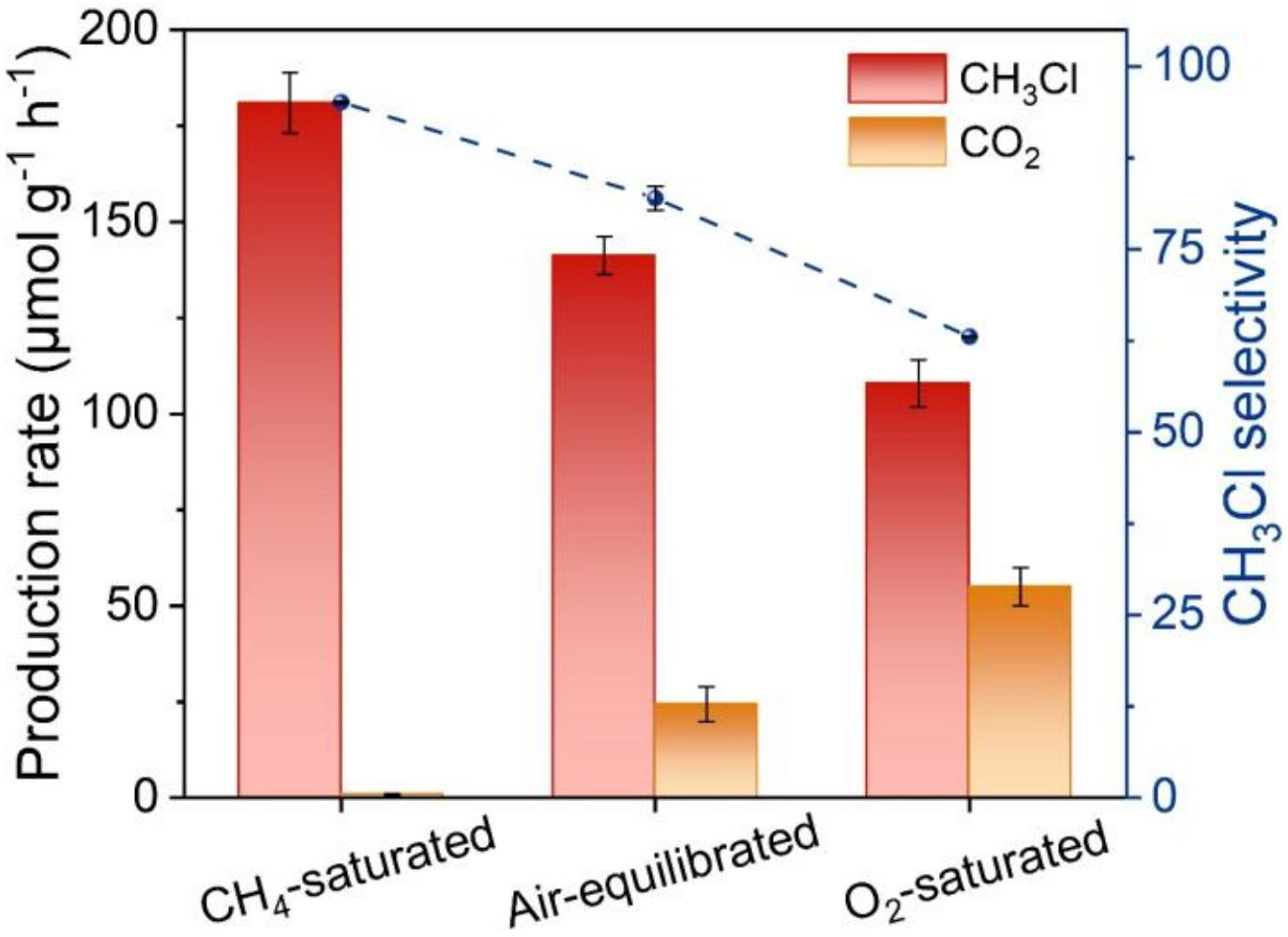

Fig. S44 | Product yields and CH_3Cl selectivity over Pt(0.13)/TiO_2@fiberglass floatable plate using CH_4-saturated, air-equilibrated, and O_2-saturated concentrated seawater containing 1 mg mL^{-1} Al^{3+} in the flow reactor,

To minimize additional oxygen introduction from external reservoirs, the reaction solution was circulated using a single peristaltic pump within the reactor under internally recirculated operation, and each measurement was conducted for 2 h to evaluate the influence of dissolved oxygen on product distribution. Under CH_4-saturated conditions, the CH_3Cl production rate reached 181 μmol g^{-1} h^{-1} with 95.1% selectivity, while CO_2 formation was largely suppressed to 0.87 μmol g^{-1} h^{-1}. In comparison, the air-equilibrated reaction solution yielded 141 μmol g^{-1} h^{-1} CH_3Cl with 82.0% selectivity and 24.4 μmol g^{-1} h^{-1} CO_2. Further enrichment with dissolved O_2 decreased the CH_3Cl production rate to 108 μmol g^{-1} h^{-1} with 63.1% selectivity, accompanied by increased CO_2 generation (55 μmol g^{-1} h^{-1}), confirming that dissolved oxygen promotes competing overoxidation pathways during continuous-flow CH_4 chlorination. Such oxygen-induced overoxidation is expected to be readily mitigated in future industrial reactor systems through closed-loop operation and continuous gas protection.

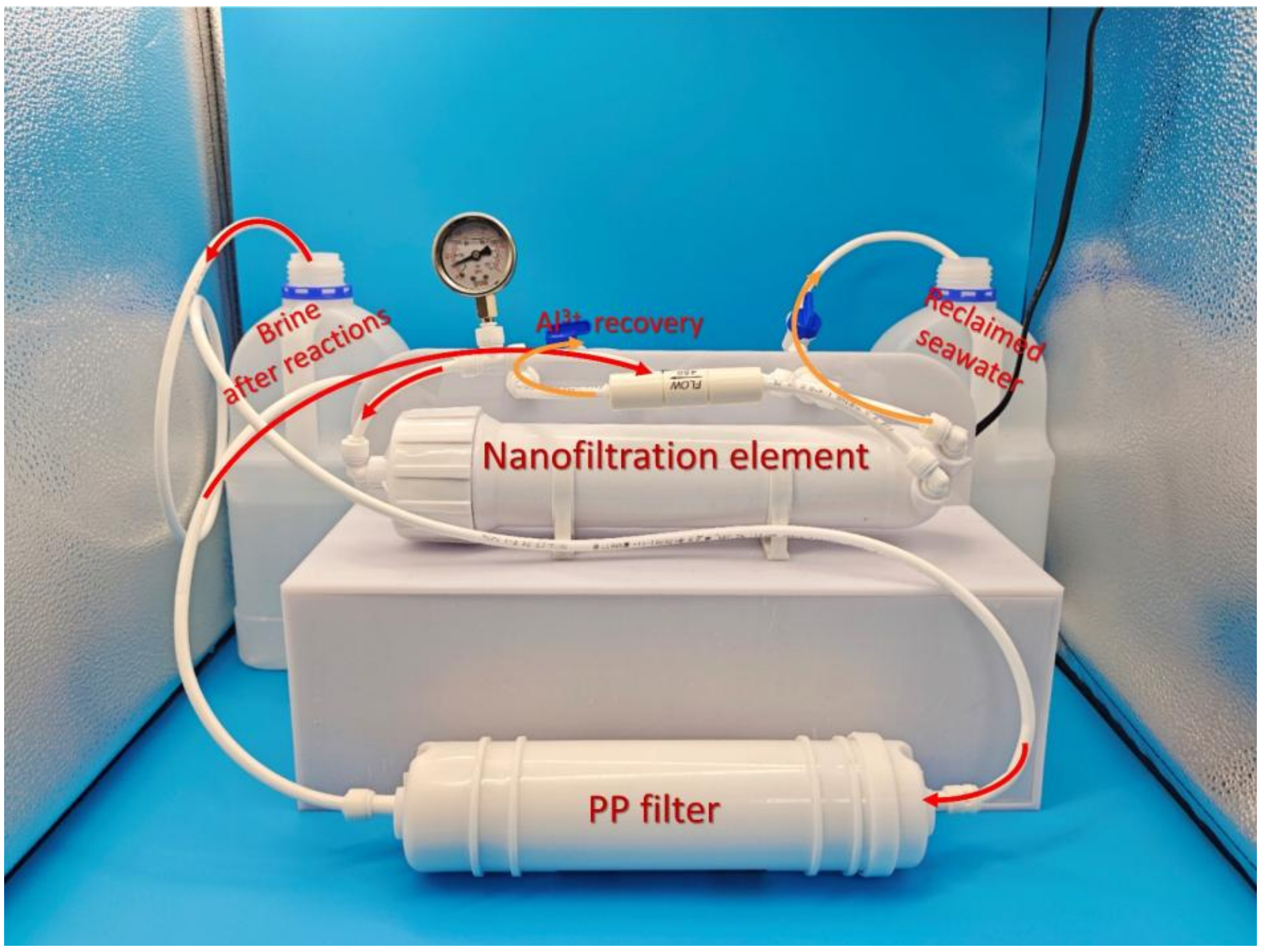

Fig. S45 | Photograph of tail water disposal plant. The vital filter is nanofiltration element.

The brine that passed through the photocatalytic flow reactor was collected as the input stream. This stream was then subjected to two-stage filtration using a PP filter and a nanofiltration element. This process produces two separate outputs: one containing a concentrated $AlCl_3$ solution for aluminum recovery, and the other consisting of Al^{3+}-depleted seawater that can be safely discharged into the ocean, supporting a sustainable cycle.

Supplementary Tables

Table S1 | Comparison of CH_3Cl production performance in this work with reported photocatalysts for methane chlorination.

Catalyst	Chlorine Source	Yields	Selectivity	Ref.
Pt(0.13)/TiO_2	$AlCl_3$ solution	2910 μmol g^{-1} h^{-1}	98.1%	This work
	NaCl solution	303 μmol g^{-1} h^{-1}	82.6%	
Pt(0.13)/TiO_2@fiberglass floatable plate	Seawater	59.7 mmol m^{-2} h^{-1} (103 μmol g^{-1} h^{-1})	79.2%	
	Seawater with 1 mg mL^{-1} $AlCl_3$	79.5 mmol m^{-2} h^{-1} (137 μmol g^{-1} h^{-1})	82.8%	
Ag_2O/NTO	NaCl solution	152 μmol g^{-1} h^{-1}	56.5%	8
	$BaCl_2$ solution	180 μmol g^{-1} h^{-1}	/	
TaOx/NTO-200	NaCl solution	233 μmol g^{-1} h^{-1}	83%	9
	HCl solution	366 μmol g^{-1} h^{-1}	92.3%	
Cu-TiO_2	NaCl with trace water	0.61 mmol m^{-2} h^{-1} (17.3 μmol g^{-1} h^{-1})*	83.7%	10
	Seawater	0.11 mmol m^{-2} h^{-1} (3.10 μmol g^{-1} h^{-1})*	/	
Pt_1/*t*-BiOCl	NaCl with trace water	53.4 μmol g^{-1} h^{-1}	96%	11

* The data is not given in the article, but is calculated based on the experimental details.

Table S2 | The contents for Pt of Pt(x)/TiO_2 photocatalyst measured by inductively coupled plasma atomic emission spectroscopy (ICP-AES).

**Samples**	**Contents for Pt**
Pt(0.03)/TiO_2	0.023 wt%
Pt(0.10)/TiO_2	0.088 wt%
Pt(0.13)/TiO_2	0.115 wt%
Pt(0.16)/TiO_2	0.150 wt%
Pt(0.13)/TiO_2 after five reaction cycles	0.111 wt%

The content for Al of Pt(0.13)/TiO_2 after five reaction cycles is 0.356 wt%.

Table S3 | Band structure of as-prepared pristine TiO_2 and Pt(x)/TiO_2.

Samples	E_g^{indir} (eV)	W_f (eV)	E_F (eV vs. NHE)	VB (eV to E_F / vs. NHE)	CB (eV vs. NHE)
Pristine TiO_2	3.04	4.00	-0.44	2.90/2.46	-0.58
Pt(0)/TiO_2	3.02	4.00	-0.44	2.89/2.45	-0.57
Pt(0.03)/TiO_2	2.61	3.96	-0.48	2.51/2.03	-0.58
Pt(0.10)/TiO_2	2.60	3.94	-0.50	2.52/2.02	-0.58
Pt(0.13)/TiO_2	2.59	3.93	-0.51	2.53/2.02	-0.57
Pt(0.16)/TiO_2	2.59	3.91	-0.53	2.56/2.03	-0.56

Note: E_g^{indir}, indirect band gap energy; W_f, work function; E_F, Fermi energy; VB, valence band; CB, conduction band; NHE, normal hydrogen electrode, is equivalent to E_V - 4.44 eV.

Table S4 | Quantitative results of Bronsted acid and Lewis acid for Pt(0.13)/TiO_2 calculated by FTIR spectra with pyridine as a probe molecule.

Desorption Temperature	Bronsted Acid Content	Lewis Acid Content
50°C	0.023 mmol g^{-1}	0.224 mmol g^{-1}
150°C	0.021 mmol g^{-1}	0.177 mmol g^{-1}
250°C	0.013 mmol g^{-1}	0.126 mmol g^{-1}
350°C	0.010 mmol g^{-1}	0.092 mmol g^{-1}

Table S5 | The ions concentration of waste water from flow tandem system before and after nanofiltration membrane filtration.

Ions Species	Before Filtration	After Once Filtration
Al^{3+}	110 ppm	1.8 ppm
Na^{+}	19635 ppm	17828 ppm
K^{+}	814 ppm	771 ppm
Mg^{2+}	2670 ppm	187 ppm
Ca^{2+}	794 ppm	171 ppm
Cl^{-}	39405 ppm	31513 ppm

Table S6 | Comparison of CH_3Cl production performance in this work with reported photocatalysts for methane chlorination.

**Approach (Catalyst)**	**Yields**	**Selectivity**	**Duration**	**Ref.**
Photocatalysis (Pt/TiO_2)	2.9 mmol g^{-1} h^{-1}<br>79.5 mmol m^{-2} h^{-1}	98.1%<br>82.8%	>15 d	This work
Electrocatalysis ($CoNi_2O_x$)	364 mmol g^{-1} h^{-1} (19.5 mA cm^{-2})<br>86.5 mmol g^{-1} h^{-1} (4.6 mA cm^{-2})	CH_3Cl/CO_2 > 400<br>CH_3Cl/CO_2 > 7	~7 h	12
Electrocatalysis (IrO_2)	~1 mmol cm^{-2} h^{-1} (240 mA cm^{-2})	Faradaic Efficiency = 34%	>8 h	13
Electrocatalysis (RuO_2)	470.9 mmol cm^{-2} h^{-1} (25.23 mA cm^{-2}, GDE)<br>104.3 mmol cm^{-2} h^{-1} (40 mA mg^{-1},GCE)	99.9%<br>>90%	15 h	14
Photoelectrocatalysis (WO_3–O_v)	104.6 mmol g^{-1} h^{-1}	71%	20 h	15
Mechanochemical (CeO_2)	2.88 mmol g^{-1} h^{-1}	95%	>3.3 h	16
Chemical (none)	~0.5 µmol g^{-1} h^{-1}	96%	4 h	17

References

1. Bzheumikhova K, Vinson J, Unterumsberger R *et al.* Titanium and titanium oxides at the K- and L-edges: Comparing theoretical calculations to X-ray absorption and X-ray emission measurements. *J Anal At Spectrom* 2023; **38**: 1885-94.

2. White MA, Johnson JA, Koberstein JT, Turro NJ. Toward the syntheses of universal ligands for metal oxide surfaces: Controlling surface functionality through click chemistry. *J Am Chem Soc* 2006; **128**: 11356-7.

3. Zhang W, Xi D, Chen Y *et al.* Light-driven flow synthesis of acetic acid from methane with chemical looping. *Nat Commun* 2023; **14**: 3047.

4. Chen F, Zhou H, Liu D *et al.* Defective zno nanoplates supported aupd nanoparticles for efficient photocatalytic methane oxidation to oxygenates. *Adv Energy Mater* 2024; **14**: 2303642.

5. Xiao Y, Feng C, Fu J *et al.* Band structure engineering and defect control of Ta_3N_5 for efficient photoelectrochemical water oxidation. *Nat Catal* 2020; **3**: 932-40.

6. Zhang C, Li H, Guo X *et al.* Experimental and theoretical studies on the effect of $Al(OH)_3$ on the fire-extinguishing performance of superfine ABC dry powder. *Powder Technol* 2021; **393**: 280-90.

7. Li X, Liu L, Liu T *et al.* An active electro-fenton PVDF/SS/PPy cathode membrane can remove contaminant by filtration and mitigate fouling by pairing with sacrificial iron anode. *J Membr Sci* 2020; **605**: 118100.

8. Li D, Shen J, Zhang J *et al.* Photocatalytic chlorination of methane using alkali chloride solution. *ACS Catal* 2022; **12**: 7004-13.

9. Li D, Lin M, Zhang J *et al.* Hydrophobic TaO_x species overlayer tuning light-driven methane chlorination with inorganic chlorine. *Small* 2024; **20**: e2402427.

10. Ma J, Zhu C, Mao K *et al.* Sustainable methane utilization technology via photocatalytic halogenation with alkali halides. *Nat Commun* 2023; **14**: 1410.

11. Yu L, Liu X, Zhang H *et al.* Twisted biocl moire superlattices for photocatalytic chloride reforming of methane. *J Am Chem Soc* 2024; **146**: 32816-25.

12. Wang Q, Li T, Yang C *et al.* Electrocatalytic methane oxidation greatly promoted by chlorine intermediates. *Angew Chem Int Ed* 2021; **60**: 17398-403.

13. Zhang Q, Chen Y, Yan S *et al.* Coupling of electrocatalytic CO_2 reduction and CH_4 oxidation for efficient methyl formate electrosynthesis. *Energ Environ Sci* 2024; **17**: 2309-14.

14. Fu Z, Zhou Y, Cao Z *et al.* Enhanced methane chlorination via RuO_2-gas convection electrode with *in-situ* generated dynamical three-phase boundaries. *Nat Commun* 2026; **17**: 2221.

15. Ma J, Mao K, Li J *et al.* Modulating chloride adsorption for efficient chloride-mediated methane conversion over tungsten oxide photoanode. *ACS Catal* 2025; **15**: 6058-66.

16. Bilke M, Losch P, Vozniuk O *et al.* Methane to chloromethane by mechanochemical activation: A selective radical pathway. *J Am Chem Soc* 2019; **141**: 11212-8.

17. Mohan V, Dutta B, Ripani R, Jain PK. Room-temperature catalyst-free methane chlorination. *Cell Rep Phys Sci* 2021; **2**: 100545.
